# Loss of TRIM31 promotes breast cancer progression through regulating K48- and K63-linked ubiquitination of p53

**DOI:** 10.1038/s41419-021-04208-3

**Published:** 2021-10-14

**Authors:** Yafei Guo, Qin Li, Gang Zhao, Jie Zhang, Hang Yuan, Tianyu Feng, Deqiong Ou, Rui Gu, Siqi Li, Kai Li, Ping Lin

**Affiliations:** 1 Lab of Experimental Oncology, State Key Laboratory of Biotherapy and Cancer Center, West China Hospital, Sichuan University, Chengdu, China; 2 Present Address: Hi-Tech Development, Keyuan 4 Road, Gaopeng Avenue, Chengdu, Sichuan 610041 P. R. China

**Keywords:** Breast cancer, Ubiquitylation

## Abstract

Breast cancer is the most common cancer in the world. Relapse and metastasis are important factors endangering the life of breast cancer patients, but the mechanism is still unclear. The stabilization of p53 is essential for preventing carcinogenesis, and ubiquitination is one of the main ways to regulate the stability of p53. Tripartite motif-containing 31 (TRIM31) is a new member of the TRIM family and functions as an E3 ubiquitin ligase. It acts as a cancer promoter or suppressor in the malignant processes of multiple cancers. However, the function of TRIM31 in breast cancer progression remains unknown. In this study, we showed that TRIM31 is downregulated in breast cancer tissues and negatively correlated with breast cancer progression. Both gain- and loss-of-function assays indicated that TRIM31 inhibits the proliferation, colony formation, migration, and invasion of breast cancer cells. Further investigation demonstrated that TRIM31 directly interacts with p53, and inducing the K63-linked ubiquitination of p53 via its RING domain, Meanwhile, TRIM31 suppresses the MDM2-mediated K48-linked ubiquitination of p53 through competitive inhibiting the interaction of MDM2 and p53, leading to the p53 stabilization and activation. Knockdown of p53 reversed the inhibitory effects of TRIM31 on the growth and metastasis of breast cancer cells. Moreover, we found that the RING and coiled-coil (C–C) domains of TRIM31 were essential for its tumor suppressor function. Taken together, our findings reveal a novel mechanism by which TRIM31 suppresses breast cancer development through the stabilization and activation of p53 and define a promising therapeutic strategy for restoring TRIM31 to treat breast cancer.

## Introduction

Breast cancer is the most commonly diagnosed cancer among women worldwide, and the latest global cancer burden data released by the World Health Organization in 2020 show that the global number of new cases of breast cancer is as high as 2.26 million, surpassing lung cancer to become the world’s most common cancer; it also ranks among the top five cancers worldwide in terms of mortality rate. In recent years, the 5-year survival period of breast cancer patients has been significantly improved, but recurrence and metastasis are still the most common causes of death in women with breast cancer [1, 2]. The recurrence and metastasis of breast cancer are related to the growth, migration, and invasion of tumor cells and are regulated by a variety of molecules. Further clarification of the molecular regulatory mechanism in breast cancer progression could provide new treatment strategies for the treatment of breast cancer.

P53 is one of the most important tumor suppressor proteins and plays a key role in regulating cell homeostasis [3–5]. The abundance and activation of p53 are mostly regulated by posttranslational modifications such as phosphorylation, acetylation, and ubiquitination [6–8]. As the key posttranslational modification event of p53, ubiquitination has become the focus of many studies [9, 10]. Several studies have reported that MDM2, which can induce K48-linked ubiquitination modification and the subsequent degradation of p53, is the most important E3 ubiquitin ligase of p53 [11, 12]. The lack of FBW7α stabilizes p53 levels and enhances the tumor suppressor function of p53 [13]. The E3 ligase RING1 promotes cancer cell proliferation and survival by targeting p53 for ubiquitination and degradation [14]. All these results indicate that P53 ubiquitination plays an important role in the occurrence and development of tumors. However, the regulatory mechanisms of p53 ubiquitination need to be further clarified.

Tripartite motif-containing 31 (TRIM31) is a newly identified member of the TRIM family. Recent studies have reported that TRIM31 acts as an E3 ubiquitin ligase and is involved in a wide range of biological processes, including autophagy, inflammation, antiviral immunity, and cancer [15–18]. Studies in cancer have indicated that TRIM31 acts as an oncogene in the progression of several tumors. TRIM31 is upregulated in hepatocellular carcinoma and induces K48-linked ubiquitination of the TSC1–TSC2 complex to promote tumorigenesis [19]. TRIM31 is overexpressed in colorectal cancer and facilitates the migration and invasion of colorectal cancer cells via the NF-κB signaling pathway [20]. TRIM31 activates the Akt signaling pathway to enhance the proliferation and metastasis of glioma cells [21]. Apart from its tumor-promoting function, TRIM31 can also exert a tumor suppressor function. For example, TRIM31 is downregulated in non-small-cell lung cancer and inhibits the growth of lung cancer cells by reducing the expression of the cell cycle regulators cyclin D1 and cyclin E [22]. However, the role and mechanism of TRIM31 in breast cancer progression are still not clear.

In this study, we showed that the expression of TRIM31 was decreased in breast cancer tissues and that its overexpression suppressed the proliferation, migration, and invasion of breast cancer cells in vitro and in vivo. Mechanistically, TRIM31 directly interacted with p53 and subsequently stabilized and activated p53 by inducing K63-linked ubiquitination as well as inhibiting MDM2-mediated K48-linked ubiquitination of p53. Moreover, we demonstrated that the RING domain and the coiled-coil (C–C) motif of TRIM31 were critical for the suppressor role of TRIM31. Overall, this study reveals a novel mechanism by which TRIM31 inhibits breast cancer development through the modulation of p53.

## Results

### TRIM31 is downregulated in human breast cancer tissues and is correlated with poor clinical outcomes

**Fig. 1:**
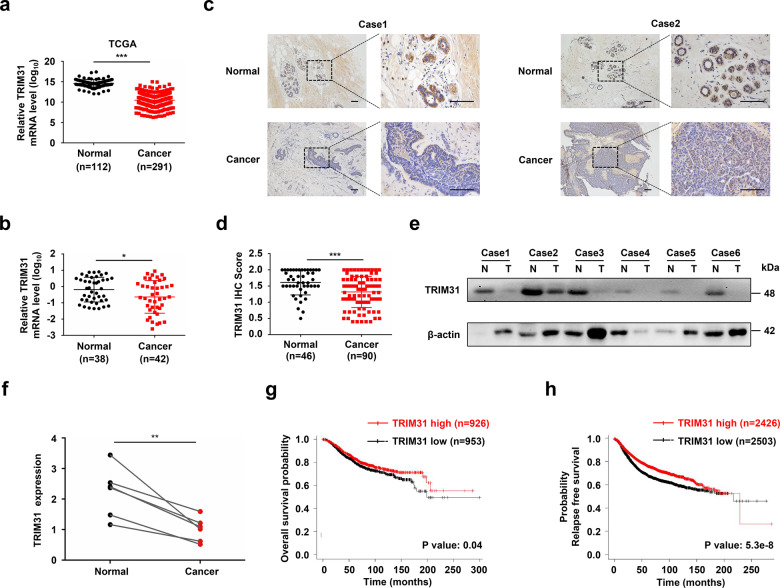
TRIM31 was reduced in breast cancer tissues. **a** The real-time PCR assay the mRNA expression of TRIM31 in breast cancer tissues and normal tissues based on the data from the TCGA database. **b** The real-time PCR assay the mRNA expression of TRIM31 in breast cancer tissues and normal breast tissues, and the data were quantified by log_10_-transformed of 2^−Δct^ values. **c** Immunohistochemistry (IHC) assays the expression of TRIM31 in 90 human breast cancer tissues and 46 normal tissues. Representative images were shown in the figure. Bar length: 100 μm. **d** The quantitative analysis of TRIM31 expression in breast cancer and normal breast tissues. **e** The TRIM31 protein expression in six paired breast cancer tissues was detected by western blot. **f** Statistical analysis of the TRIM31 expression in breast cancer tissues and their paired adjacent normal tissues. **g**, **h** The Kaplan–Meier plot of overall and relapse-free survival by the expression of TRIM31 in the breast cancer patients, the data was carried out from the Kaplan–Meier Plotter. Data are shown as the mean ± SD of at least three independent experiments, and the significant level was identified by *P < 0.05, **P < 0.01, and ***P < 0.001.

To explore the function and clinical relevance of TRIM31 in human breast cancer, we first analyzed the mRNA expression of TRIM31 in breast cancer by using RNA-seq data from The Cancer Genome Atlas (TCGA). The results showed that TRIM31 mRNA expression was markedly decreased in breast cancer tissues compared with normal breast tissues (Fig. 1a). Next, we examined the mRNA levels of TRIM31 in breast cancer patients by quantitative real-time polymerase chain reaction (PCR). We found that TRIM31 expression was markedly lower in cancer tissues than in normal breast tissues (Fig. 1b). Furthermore, we analyzed TRIM31 expression in breast cancer patients by immunohistochemical staining with a TRIM31 antibody. The data indicated that the expression of TRIM31 was significantly reduced in breast cancer tissues (Fig. 1c, d). A similar result was confirmed by western blotting using an anti-TRIM31 antibody (Fig. 1e, f). Subsequently, we evaluated the correlation of TRIM31 expression with the clinicopathological characteristics of breast cancer patients. Our analysis showed that decreased expression of TRIM31 was associated with larger tumor size, higher Ki67 expression, advanced TNM stage, advanced histological grade, and lymph node invasion (Table 1). More importantly, Kaplan–Meier analysis revealed that breast cancer patients with low TRIM31 levels had worse survival outcomes than those with high levels in the cancer dataset from the Kaplan–Meier Plotter (Fig. 1g, h). Altogether, these results indicate that TRIM31 expression is downregulated in breast cancer and that it may be a valuable prognostic biomarker in breast cancer patients.

**Table 1:** The correlation of TRIM31 expression and clinicopathological characteristics of breast cancer patients.

Parameters	Cases	TRIM31 expression		*P* values
		Low	High	
	90	41	49	
*Age(years)*				0.572
<60	70	33	37	
≥60	20	8	12	
*ER status*				0.277
Negative	34	13	21	
Positive	56	28	28	
*PR status*				0.372
Negative	35	18	17	
Positive	55	23	32	
*HER2 status*				0.447
Negative	65	28	37	
Positive	25	13	12	
*P53*				0.942
Wide type	47	22	25	
Mutant	28	12	16	
Deletion	15	7	8	
*Ki-67 status*				**0.003**
Negative	25	5	20	
Positive	65	36	29	
*CK5/6 status*				0.199
Negative	69	34	35	
Positive	21	7	14	
*E-cad status*				0.314
Negative	26	14	12	
Positive	64	27	37	
*P63 status*				0.497
Negative	73	32	41	
Positive	17	9	8	
*Tumor size (cm)*				**0.020**
≤2	36	11	25	
>2	54	30	24	
*TNM stage*				**0.006**
I	21	5	16	
II	50	22	28	
III–IV	19	14	5	
*Histological grade*				**<0.001**
I–II	38	8	30	
III	52	33	19	
*Lymph node invasion*				**0.008**
Negative	51	17	34	
Positive	39	24	15	

### TRIM31 suppresses the proliferation, migration, and invasion of breast cancer cells

**Fig. 2:**
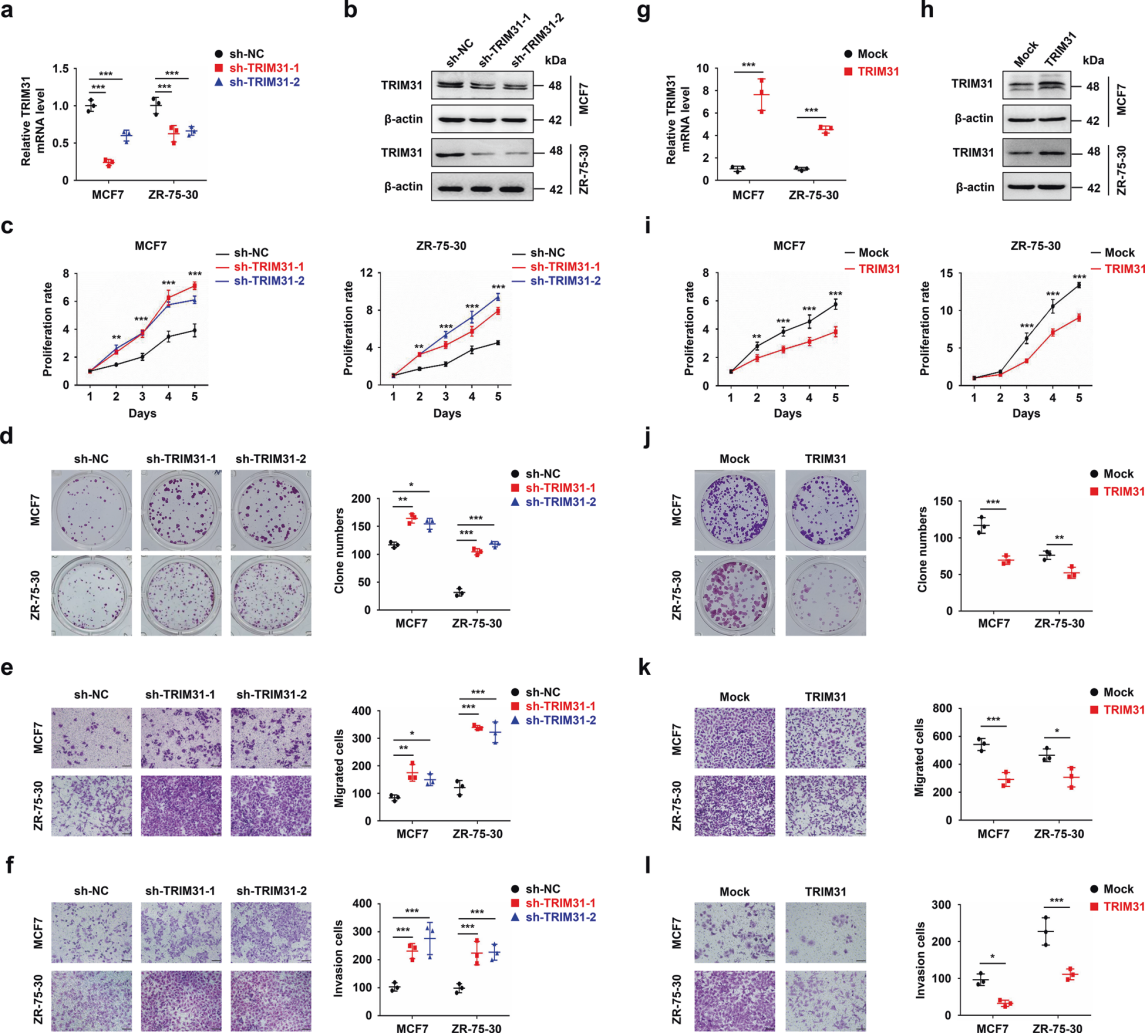
TRIM31 suppressed the proliferation, migration and invasion of breast cancer cell lines. **a**, **b** The real-time PCR (**a**) and western blot (**b**) assay of TRIM31 interfering in MCF7 and ZR-75-30 cells. **c**, **d** CCK8 (**c**) and colony formation (**d**) assay were performed to detect the proliferation of MCF7 and ZR-75-30 cells stably interfere with TRIM31 or control. **e**, **f** The migration (**e**) and invasion (**f**) assay of TRIM31-interference and control of MCF7 and ZR-75-30 cells, bar length: 100 μm. **g**, **h** The real-time PCR (**g**) and western blot (**h**) assay of TRIM31 overexpression in MCF7 and ZR-75-30 cells. **i**, **j** The CCK8 (**i**) and colony formation (**j**) assay of MCF7 and ZR-75-30 cells stably overexpress TRIM31 or control. **k**, **l** The migration (**k**) and invasion (**l**) assay of MCF7 and ZR-75-30 cells stably overexpress TRIM31 or control, Bar length: 100 μm. Data are shown as the mean ± SD of at least three independent experiments, and the significant level was identified by *P < 0.05, **P < 0.01, and ***P < 0.001.

To investigate the potential function of TRIM31 in breast cancer progression, we silenced TRIM31 expression in MCF7 and ZR-75-30 cells using lentivirus-delivered shRNAs (the sequences of sh-TRIM31 were shown in Table 2), and the level of TRIM31 was detected by real-time PCR and western blotting (Fig. 2a, b). The Cell Counting Kit-8 (CCK-8) assay showed that TRIM31 suppression increased the viability of breast cancer cells (Fig. 2c). Consistently, the colony numbers were significantly increased in TRIM31-interfering cells compared with mock cells (Fig. 2d). In addition, downregulation of TRIM31 increased the migratory and invasive capacities of MCF7 and ZR-75-30 cells (Fig. 2e, f). We next used a lentivirus-mediated expression system to stably overexpress TRIM31 in MCF7 and ZR-75-30 breast cancer cell lines. Real-time PCR and western blot assays indicated that TRIM31 was successfully up-regulated in MCF7 and ZR-75-30 cells (Fig. 2g, h). TRIM31 overexpression significantly suppressed breast cancer cell proliferation and colony formation, as determined by CCK-8 and colony formation assays, respectively (Fig. 2i, j). Moreover, upregulation of TRIM31 inhibited the migration and invasion of breast cancer cells (Fig. 2k, l). These data indicate that TRIM31 inhibits the growth and metastasis of breast cancer cells, suggesting a tumor-suppressive role for TRIM31 in breast cancer.

### TRIM31 directly binds to the p53 protein

**Fig. 3:**
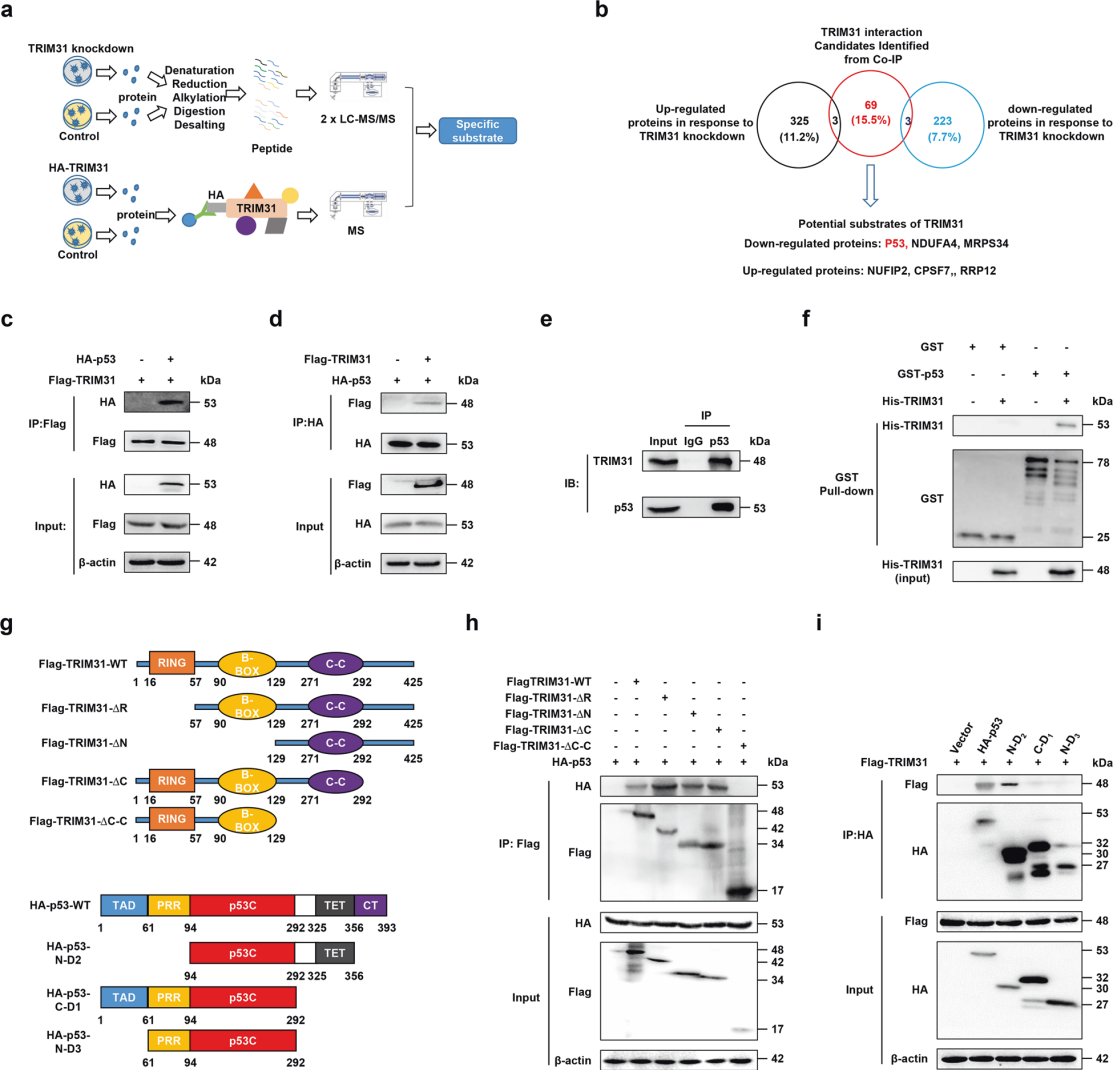
TRIM31 interacted with P53. **a** The diagram showed the process for the quantitative proteomics and CO-IP-MS. **b** The Venn diagram showed the number of significant upregulated (black) and downregulated (blue) proteins identified in response to TRIM31 knockdown, and the protein expression fold change is MCF7-1/MCF7-NC > 1.5 or <0.67, and unique peptide ≧ 2 is defined as a significantly different protein. TRIM31 interaction candidates were identified in the Co-IP process (red). The below showed potential substrates of the TRIM31. **c**, **d** The HEK293 cells were co-transfected with Flag-TRIM31 and HA-P53 plasmids for 48 h, and then the cells were treated with MG132 (20 μM) for 4 h. The lysates of cells were immunoprecipitated with Flag-tag antibody and then western blot assay with HA-tag antibody (**c**); immunoprecipitated with HA-tag antibody and immunoblotted with Flag-tag antibody (**d**). **e** The MCF7 cell lysates were immunoprecipitated with Ig G and anti-p53 antibody and the expression of TRIM31 and p53 were detected by western blot. **f** GST-pull down was performed to verify the direct interaction of TRIM31 and P53 and the components were detected by the western blot assay with anti-GST and anti-His antibody. **g** The schematic diagram showed structural domains of TRIM31 and p53 protein. **h** HEK293 cells were transfected with HA-P53 and Falg-TRIM31 or several TRIM31 deletion mutants. The whole-cell lysates were immunoprecipitated with anti-Flag beads and immunoblotted with anti-Flag and anti-HA antibodies. **i** HEK293 cells were co-transfected with Falg-TRIM31 and HA-P53 or several P53 deletion mutants. The whole-cell lysates were immunoprecipitated with anti-HA antibodies and immunoblotted with anti-Flag and anti-HA antibodies.

To elucidate the molecular mechanism of TRIM31-mediated anti-tumor function in breast cancer, we used proteomics and coimmunoprecipitation-mass spectrometry (MS) to characterize the specific substrate proteins of TRIM31 (Fig. 3a). We identified 223 downregulated proteins and 325 upregulated proteins in TRIM31-deficient MCF7 cells (Supplementary Table S1). Moreover, we obtained 69 out of 444 proteins that potentially interacted with TRIM31 (Supplementary Table S2). We overlapped the above results and found six proteins predicted to be substrates of TRIM31 (Fig. 3b). Considering that p53 regulates many target genes that contribute to the progression of many cancers, we chose p53 for further investigation.

To verify the association between TRIM31 and p53, HEK293 cells were co-transfected with Flag-TRIM31 and HA-p53 and then subjected to a reciprocal co-immunoprecipitation (co-IP) assay. The results indicated that TRIM31 interacted with p53 in HEK293 cells (Fig. 3c, d). A similar result was observed in MCF7 cells under physiological conditions (Fig. 3e). To further define whether TRIM31 directly interacts with p53, we expressed and purified recombinant GST-p53 and His-TRIM31 proteins in vitro. The elution of GST-p53 and His-TRIM31 protein was validated by sodium dodecyl-sulfate polyacrylamide gel electrophoresis (SDS-PAGE) (Supplementary Fig. S1). GST pull-down assays showed that TRIM31 is directly bound to p53 (Fig. 3f). TRIM31 consists of three domains: a RING-finger domain (amino acids 16–57), a B-box domain (amino acids 90–129) and a C–C domain (amino acids 126–307). To identify which domain of TRIM31 is essential for its binding with p53, we generated several truncating mutations of TRIM31 (Fig. 3g). The co-IP results showed that the TRIM31-WT, TRIM31-∆R, TRIM31-∆N, and TRIM31-∆C mutants retained their association with p53, whereas the Flag-TRIM31-∆C–C mutant did not interact with p53 (Fig. 3h). P53 is composed of an N-terminal transactivation domain (TAD, amino acids 1–61), a proline-rich domain (amino acids 62–94), a DNA-binding domain (p53C, amino acids 95–292), a tetramerization domain (amino acids 325-356) and an extreme C-terminus (CT, amino acids 357–393), and we also generated three p53 truncating mutants (N-D2, C-D1, N-D3) to test their ability to bind with TRIM31 (Fig. 3g). We observed decreased interaction of p53 with the TRIM31 in C-D1 and N-D3 p53 mutants but not the N-D2 p53 mutant (Fig. 3i). These results suggest that the C–C domain of TRIM31 and the CT domain of p53 are crucial for the association between TRIM31 and p53.

### TRIM31 stabilizes and activates the p53 protein by inducing K63-linked ubiquitination and inhibiting K48-linked ubiquitination in breast cancer cells

**Fig. 4:**
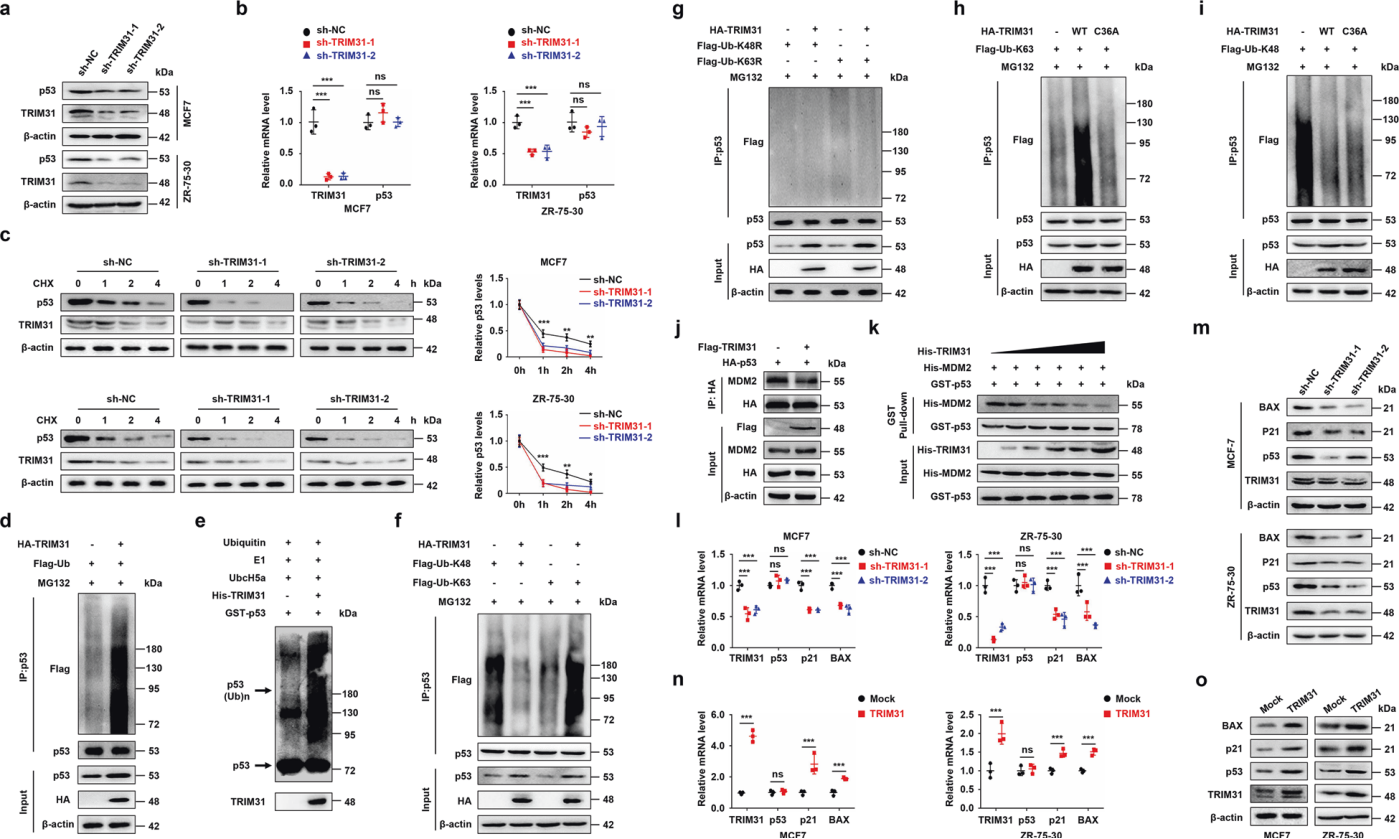
TRIM31 stabilized and activated p53 by inducing the K63 ubiquitination of p53 and inhibiting its K48 ubiquitination by disrupting the MDM2-P53 complex. **a** The MCF7 and ZR-75-30 cells stably interfere with TRIM31 or control, the cell lysates were immunoblotted with anti-TRIM31, anti-p53, and anti-β-actin antibodies. **b** The qPCR was used to detect the mRNA expression of TRIM31 and P53 in MCF7 and ZR-75-30 cells. **c** The MCF7 and ZR-75-30 cells stably interfere with TRIM31 or control were treated with cyclohexamide (CHX) for 0, 1, 2 and 4 h, the expression of TRIM31 and P53 were detected by western blot. The line chart was further quantitatively analyzed TRIM31 expression. **d** The MCF7 cells were co-transfected with HA-TRIM31 and Falg-ub plasmids. The cell lysates were immunoprecipitated by anti-P53 antibody, and then western blot assay with anti-Falg, anti-P53, and anti-HA antibody. **e** The vitro ubiquitination assay was performed and then the western blot assay with the anti-p53 antibody. **f** The MCF7 cells were co-transfected with HA-TRIM31, Flag-Ub-K48, and Flag-Ub-K63 plasmids, then the cells lysates were pulled down with P53 antibody and analyzed by western blot. **g** The MCF7 cells were transfected with HA-TRIM31, Flag-Ub-k48R, and Flag-Ub-K63R plasmids. The cell lysates were immunoprecipitated with anti-P53 antibody and then western blot assay with Flag-tag antibody. **h**, **i** The MCF7 cells was transfected with HA-TRIM31 or HA-TRIM31-36A together with Flag-Ub-K63 (**h**) and Flag-Ub-K48 (**i**) plasmids. The cell lysates were immunoprecipitated with anti-P53 antibody and then western blot assay with anti-Flag antibody. **j** The MCF7 cells were co-transfected with HA-P53 and Flag-TRIM31 plasmids, the lysates of cells were immunoprecipitated with anti-HA beads and then western blot assay with anti-Flag, anti-HA, and anti-MDM2 antibody. **k** Pull-down assay was performed to detect the interaction of TRIM31 and p53 and the proteins were immunoblotted with anti-His, GST antibody. **l**, **m** The MCF7 and ZR-75-30 cells stably interfere with TRIM31 or control, the real-time PCR assay of the mRNA expression of TRIM31, P53, P21, and BAX in MCF7 and ZR-75-30 cells (**l**). Western blot assay of the expression of TRIM31, P53, P21, and BAX protein in MCF7 and ZR-75-30 cells (**m**). **n**, **o** The MCF7 and ZR-75-30 cells stably overexpress TRIM31 or control, the real-time PCR assay of the mRNA expression of TRIM31, P53, P21 and BAX in MCF7 and ZR-75-30 cells (**n**). Western blot assay of the expression of TRIM31, P53, P21 and BAX protein in MCF7 and ZR-75-30 cells (**o**). Data are shown as the mean ± SD of at least three independent experiments, and the significant level was identified by *P < 0.05, **P < 0.01, and ***P < 0.001.

We next determined the effect of TRIM31 on p53 expression in breast cancer cells. TRIM31 knockdown significantly reduced the protein expression of p53 in MCF7 and ZR-75-30 cells, while TRIM31 overexpression increased the protein expression of p53 (Fig. 4a and Supplementary Fig. S2a). Notably, TRIM31 did not alter the mRNA level of p53 (Fig. 4b and Supplementary Fig. S2b). These results suggested that TRIM31 regulates p53 protein expression at the posttranslational level. We further verified whether TRIM31 impacted the stability of p53. A cyclohexamide chase assay showed that knockdown of TRIM31 accelerated the degradation of p53 protein in MCF7 and ZR-75-30 cells (Fig. 4c). As TRIM31 is an E3 ubiquitin ligase, we next explored the effect of TRIM31 on p53 ubiquitination. We found that overexpression of TRIM31 increased the polyubiquitination of p53 in MCF7 cells (Fig. 4d). The in vitro ubiquitination assay also confirmed that p53 was a direct substrate of TRIM31 (Fig. 4e). K48- and K63-linked polyubiquitin chains are the main types of ubiquitin linkages: K48-linked polyubiquitination induces degradation of target proteins, and K63-linked polyubiquitination regulates the activation of target proteins [23, 24]. We further characterized which type of ubiquitination of p53 was regulated by TRIM31. MCF7 cells were cotransfected with HA-TRIM31 and K48 or K63 ubiquitin plasmids (containing only K48 or K63, respectively). The cells were subjected to an in vivo ubiquitination assay. As shown in Fig. 4f, TRIM31 promoted the K63-linked ubiquitination of p53 and inhibited the K48-linked ubiquitination of p53. However, this effect was abolished by the K48R and K63R ubiquitin plasmids (Fig. 4g). Moreover, the TRIM31 C36A mutant (without E3 ligase activity [19]) failed to potentiate the K63-linked ubiquitination of p53, but it also could inhibit the K48-linked ubiquitination of p53 (Fig. 4h, i). These results indicated that TRIM31 induced the K63 ubiquitination of p53 via its E3 ubiquitin ligase function. However, the inhibitory effect of TRIM31 on the K48 ubiquitination of p53 does not depend on its E3 ubiquitin ligase function. MDM2 has been reported to facilitate the degradation of p53 by K48-linked polyubiquitination [11]. We next detected whether TRIM31 suppressed the association of p53 and MDM2 to inhibit the K48 ubiquitination of p53. The coimmunoprecipitation results showed that TRIM31 significantly weakened p53 and MDM2 complex formation (Fig. 4j). Moreover, we found that TRIM31 had no effect on the MDM2 protein level, and TRIM31 failed to interact with MDM2 (Supplementary Fig. S3). Thus, we speculated that TRIM31 and MDM2 are competitively bound to p53. To test this hypothesis, we purified recombinant GST-P53, His-TRIM31, and His-MDM2 protein and then carried out pull-down assays in vitro (Supplementary Fig. S4). The results showed that the binding affinity of P53 with MDM2 was gradually decreased by increasing amounts of His-TRIM31 (Fig. 4k). We further performed the bimolecular fluorescence complementation (BiFC) assay, which enables the visualization of protein–protein interactions in living cells, to prove whether TRIM31 competitively inhibited the interaction of MDM2 and p53. We observed declined fluorescence intensity of p53 and MDM2 complex in TRIM31 overexpressing ZR-75-30 cells compared to control cells (Supplementary Fig. S5). In addition, we examined the dose effects of MDM2 overexpression on the interaction of TRIM31 and endogenous p53. Our results showed that upregulated MDM2 promoted the disassociation of TRIM31 and p53 complex in a dose-dependent manner (Supplementary Fig. S6). We next assessed the effect of TRIM31 on the expression of molecules downstream of p53 signaling. Our results showed reduced transcription of the p53 target genes p21 and BAX in TRIM31-deficient breast cancer cells (Fig. 4l). A similar result was confirmed by western blotting (Fig. 4m). In contrast, overexpression of TRIM31 significantly altered both the mRNA and protein levels of p21 and BAX (Fig. 4n-o). Taken together, these data indicate that TRIM31 enhances the K63-linked ubiquitination of p53 and suppresses the MDM2-mediated K48-linked ubiquitination of p53 by disrupting the MDM2–p53 complex, which stabilizes and activates the p53 protein.

### P53 is indispensable for the tumor-suppressive function of TRIM31 in breast cancer

**Fig. 5:**
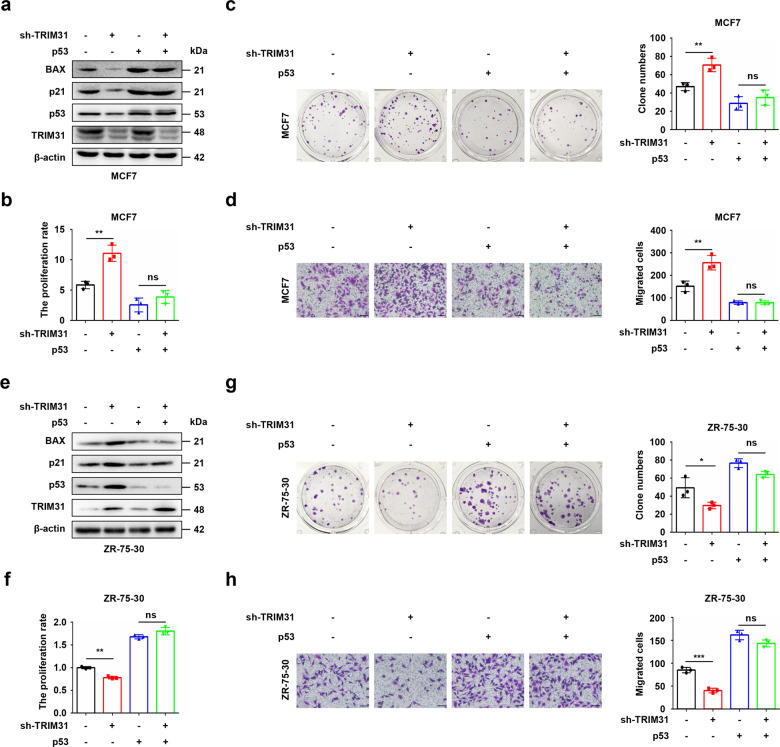
TRIM31 exerts the tumor-suppressive function via p53. **a** The MCF7 cells stably interfere with TRIM31 were transfected with Flag-p53 plasmid, and the expression of TRIM31 and p53 were detected by western blot. **b** The cell survival of MCF7 at 72 h was detected by CCK8 assay. **c** The colony formation assay was performed to detect the cell proliferation of MCF7 cells. **d** The transwell migration assay was performed to detect the migration capability of MCF7 cells. **e** The ZR-75-30 cells stably overexpress TRIM31 were transfected with p53 special shRNA, and the TRIM31 and p53 expression were detected by western blot. **f** The CCK8 analyzed the cell survival of ZR-75-30 at 72 h. **g** The colony formation was performed to detect the cell proliferation of ZR-75-30 cells. **h** The transwell migration assay was performed to detect the migration capability of ZR-75-30, Bar length: 100 μm. Data are shown as the mean ± SD of at least three independent experiments, and the significant level was identified by *P < 0.05, **P < 0.01 and ***P < 0.001.

To further determine whether p53 was required for the TRIM31-mediated tumor suppression function in breast cancer, the Flag-p53 plasmid was transfected into TRIM31-deficient MCF7 cells. Western blot assays showed that TRIM31 was successfully knocked down and p53 was successfully overexpressed. Our data further indicated that overexpression of p53 could rescue knockdown of TRIM31-regulated suppression of the p53 signaling pathway (Fig. 5a). Further CCK-8, colony formation, and Transwell assays showed that p53 rescued the anti-tumor function of TRIM31 in MCF7 cells (Fig. 5b–d). Moreover, we further investigated the effect of MDM2 on TRIM31-mediated tumor growth, TRIM31-deficient MCF7 cells were introduced sgRNA targeting MDM2. Our results showed that the knockout of MDM2 restored the inactivation of the p53 signaling pathway caused by TRIM31 interference (Supplementary Fig. S7a). In addition, TRIM31 deficiency-mediated increased tumor growth was abolished by the knockout of MDM2 (Supplementary Fig. S7b, c). Next, ZR-75-30 cells were cotransfected with HA-TRIM31 and p53-specific shRNA plasmids, and the western blotting results suggested that TRIM31 was successfully overexpressed and p53 was silenced. Moreover, the knockdown of p53 reversed TRIM31 overexpression-activated p53 signaling (Fig. 5e). Furthermore, p53 knockdown restored the ability of TRIM31 to suppress the proliferation, colony formation, and migration of ZR-75-30 cells (Fig. 5f–h). To further confirm that p53 significantly reversed the tumor suppressor function of TRIM31, TRIM31-deficient MCF7 cells or TRIM31-overexpressing ZR-75-30 cells were treated with the p53 activator nutlin-3 or the p53 inhibitor pifithrin-α. Activation of p53 reversed the knockdown of TRIM31-mediated suppression of the p53 signaling pathway (Supplementary Fig. S8a). Furthermore, CCK-8, colony formation, and Transwell assays showed that activation of p53 rescued the anti-tumor function of TRIM31 in MCF7 cells (Supplementary Fig. S8b–d). Moreover, inhibition of p53 reversed TRIM31 overexpression-induced promotion of the p53 signaling pathway (Supplementary Fig. S9a). Furthermore, inhibition of p53 rescued the inhibitory effects of TRIM31 on the proliferation, colony formation, and migration of ZR-75-30 cells (Supplementary Fig. S9b–d). These data further revealed that TRIM31 suppresses breast cancer progression via the p53 protein.

### TRIM31 acts as a tumor suppressor in breast cancer through its functional domains

**Fig. 6:**
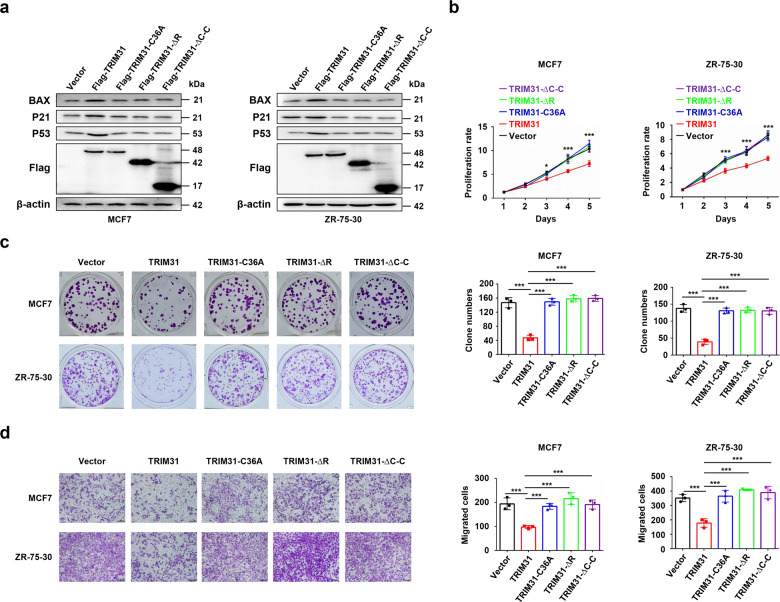
The coiled-coil domain and the RING domain of TRIM31 were critical for the suppressor role of TRIM31. The MCF7 and ZR-75-30 cells were transfected with Flag-TRIM31 plasmid or TRIM31 truncation mutant (TRIM31-ΔRING or TRIM31-ΔC–C) plasmids or Flag-TRIM31-C36A plasmid, and the expression of TRIM31 and its truncation mutants were detected by western blot (**b**–**d**). The proliferation (**b**), colony formation (**c**), and migration (**d**) of breast cancer cells were further detected and analyzed, Bar length: 100 μm. Data are shown as the mean ± SD of at least three independent experiments, and the significant level was identified by *P < 0.05, **P < 0.01, and ***P < 0.001.

We have demonstrated that the C–C domain of TRIM31 is crucial for the interaction of TRIM31 and p53 and that the RING domain is necessary for the E3 ubiquitin ligase function of TRIM31. Therefore, we further investigated whether these two domains are essential for the tumor suppressor function of TRIM31 in breast cancer. Our results suggested that either deletion of the C–C domain (∆C–C) or deletion (∆R) or mutation (C36A) of the RING domain in TRIM31 efficiently rescued the TRIM31-mediated suppression of p53 signaling in MCF7 and ZR-75-30 cells (Fig. 6a), which demonstrated that TRIM31 inhibits p53 signaling through these two domains. Further study showed that overexpression of the ∆C–C, ∆R, and C36A mutants of TRIM31 could significantly reduce TRIM31-induced suppression of proliferation (Fig. 6b), colony formation (Fig. 6c), and migration (Fig. 6d) in MCF7 and ZR-75-30 cells. Collectively, these results suggest that the C–C and RING domains were essential for the tumor suppressor function of TRIM31 in breast cancer.

### TRIM31 suppresses the growth and metastasis of breast cancer cells in vivo

**Fig. 7:**
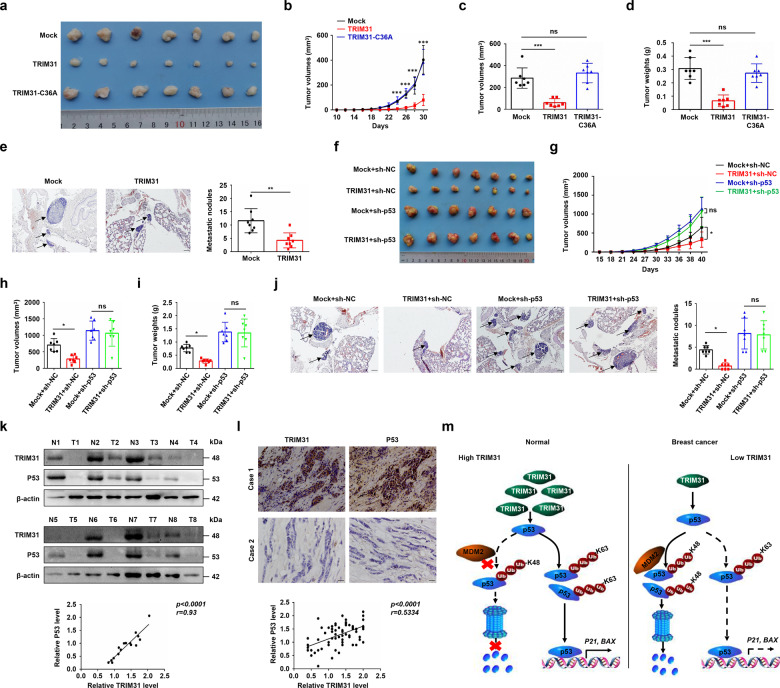
Overexpression of TRIM31 suppresses the tumorigenicity in nude mice models and correlation analysis of TRIM31 and p53 in breast cancer tissues. **a**–**d** About 5 × 10^6^ of ZR-75-30 cells stably overexpressing TRIM31 and control were injected into both backs of mice. The images showed the excised tumors from TRIM31-overexpression ZR-75-30 cells and control cells (**a**). The growth curve (**b**), tumor volume (**c**), and tumor weight (**d**) of the TRIM31-overexpression group compared with the control group were statistically analyzed at 30 days after injection. **e** About 5 × 10^6^ of the ZR-75-30 cells stably overexpress TRIM31 or control were injected into nude mice by tail-vein injection. Representative pictures of HE staining of lung tissue (left), the arrows indicated the metastatic nodules on the surface of lung tissues. Bar length: 100 μm. The number of nodules on lung tissues was quantified at 45 days after tail–vein injection (right). **f**–**i** About 3 × 10^6^ of ZR-75-30 cells stably express HA-TRIM31, sh-p53, and control (Mock+sh-NC, TRIM31 + sh-NC, Mock + sh-p53, TRIM31 + sh-p53) were injected into mammary fat pads of 6-week old female nude mice. The images showed the tumors from these four groups (**f**). The growth curve (**g**), tumor volume (**h**), and tumor weight (**i**) of these four groups were statistically analyzed at 40 days after injection. **j** Representative pictures of HE staining of lung tissues (left), the arrows indicated the metastatic nodules on the surface of lung tissues. Bar length: 100 μm. The number of nodules on the lungs was quantified at 40 days after injection (right). **k** The expression of TRIM31 and p53 in eight paired breast cancer patient tissues were analyzed by western blot (up). Pearson correlation analysis was used to measure the correlation of TRIM31 and p53 (down). **l** Representative images of IHC staining of TRIM31 and p53 in normal and breast cancer tissues (up). Bar length: 100 μm. Correlations analysis of the expression of TRIM31 and p53 protein (down). **m** The model of the antitumor function of TRIM31 in breast cancer progression. In normal cells, the expression of TRIM31 was upregulated. On the one hand, TRIM31 can directly bind to p53 and induce the K63 ubiquitination of p53 to stabilize and activate the p53 signal, on the other hand, TRIM31 can suppress the K48-linked ubiquitination and proteasome degradation of p53 by disrupting the binding of MDM2 and p53. In breast cancer cells, the expression of TRIM31 was downregulated, the p53 was mainly controlled by MDM2 that mediate the K48 polyubiquitination of p53 for degradation.

To verify whether TRIM31 was involved in the malignant progression of breast cancer cells in vivo, we first established a xenograft tumor model by subcutaneous injection of TRIM31 or TRIM31-C36A-overexpressing ZR-75-30 cells and control cells into nude mice, respectively. We found that TRIM31 up-regulation significantly delayed tumor formation, while TRIM31-C36A mutant failed to suppress tumor growth (Fig. 7a, b). Similar results were obtained in tumor volumes and weights assays (Fig. 7c, d). To further research the effects of TRIM31 on breast cancer cell metastasis, nude mice were implanted with the indicated ZR-75-30 cells by tail vein injection. The results showed that a decreased number of lung metastasis nodules was observed in TRIM31-overexpressing ZR-75-30 cells compared with mock cells (Fig. 7e).

To further identify whether p53 contributed to the tumor-suppressive function of TRIM31 in vivo, the indicated ZR-75-30 cells were injected into mammary fat pads of 7-week-old female nude mice. TRIM31 overexpression suppressed the growth of breast cancer cells, and this effect was reversed by p53 knockdown (Fig. 7f). Similar results were confirmed by growth curve, tumor volume, and tumor weight analysis (Fig. 7g–i). Consistently, p53 knockdown efficiently compromised the inhibitory effect of TRIM31 on the motility of ZR-75-30 cells (Fig. 7j). In addition, we also analyzed the effect of MDM2 overexpression on Trim31-mediated tumor growth suppression. The data indicated that overexpression of TRIM31 inhibited the growth of breast cancer cells, and this effect was rescued by MDM2 overexpression (Supplementary Fig. S10). In general, p53 was required for TRIM31-mediated suppression of the growth and metastasis of breast cancer cells in vivo.

### TRIM31 and P53 are positively correlated in breast cancer patients

To detect the correlation between the expression of TRIM31 and p53 in breast cancer patient tissues, we extracted protein from cancer tissue and cancer-adjacent tissue from 8 breast cancer patients, and the expression of TRIM31 and p53 was analyzed by western blotting. Pearson correlation analysis indicated that the expression of TRIM31 protein was positively correlated with that of p53 protein in breast cancer tissues (Fig. 7k). Immunohistochemistry (IHC) staining of cancer tissues from breast cancer patients showed that both TRIM31 protein and p53 protein were highly expressed or expressed at low levels in breast cancer tissues. Correlation analysis indicated that the protein expression of TRIM31 was significantly positively correlated with the protein expression p53 (Fig. 7l). In addition, we analyzed the correlation of TRIM31 expression and p53 status in breast cancer, and the result showed that TRIM31 expression was not associated with p53 status (Table 1). Similar results were obtained in breast cancer patients from the Kaplan–Meier Plotter database (Supplementary Table S3). Together, these results showed that the expression of TRIM31 and P53 is positively correlated in breast cancer tissues.

In summary, our data suggest that TRIM31 acts as a positive regulator of p53 and plays a tumor-suppressive role in breast cancer by stabilizing and activating the p53 signaling pathway (Fig. 7m).

## Discussion

In this study, we verified that TRIM31 expression was downregulated in breast cancer and that low TRIM31 levels were associated with worse survival outcomes than high levels in breast cancer patients. Moreover, low TRIM31 expression was associated with larger tumor size, higher Ki67 expression, advanced TNM stage, advanced histological grade, and lymph node invasion. Further cell experiments showed that overexpression of TRIM31 significantly inhibited cell proliferation, colony formation, migration, and invasion. All these data suggest that TRIM31 acts as a tumor suppressor in breast cancer and may be a valuable prognostic biomarker in breast cancer patients.

TRIM31 is a member of the TRIM family, and the proteins of this family have been reported to play important roles in cancer progression. Several TRIM family proteins have been reported to be involved in the regulation of p53. For example, TRIM67 directly interacts with p53 and inhibits p53 degradation via its ubiquitin ligase MDM2 to suppress colorectal cancer initiation and progression [25]. TRIM32 can decrease p53 activity by inducing ubiquitination and proteasomal degradation of p53 [26]. TRIM45 stabilizes p53 through K63-linked ubiquitination to suppress glioma progression [27]. In this study, we identified that TRIM31 stabilizes and activates p53 to suppress breast cancer progression. Further study showed that TRIM31 directly binds to p53 and induces the K63-linked ubiquitination of p53, while it disrupts the MDM2-mediated K48-linked ubiquitination of p53. MDM2 is the main E3 ubiquitin ligase of p53. MDM2 interacts with the p53 N-terminal TAD domain, central DNA binding domain, and CT, and each domain is necessary for MDM2-mediated regulation of p53 [28, 29]. We found that TRIM31 binds to the CT of p53 and may inhibit the effect of MDM2 by occupying the CT of p53; this may occur as a result of the destruction of the p53–MDM2 complex, which prevents MDM2-mediated p53 ubiquitination and proteasomal degradation. Altogether, these data indicate that TRIM31 is a new member of the TRIM family that ubiquitinates p53 and highlight a new regulatory mechanism of P53.

P53 is one of the most important tumor suppressor proteins and triggers cell cycle arrest, apoptosis, and metastasis inhibition in cancer cells. Due to its almost universal changes in cancer, p53 is an attractive target for developing new targeted therapies for cancers [30]. However, until now, there has been no clinical treatment targeting p53, so p53 was widely regarded as “undruggable”. Nevertheless, this situation has changed, and it has been demonstrated that the recovery and stabilization of wild-type p53 can lead to tumor suppression in many different animal models. For example, MDM2 is the main E3 ubiquitin ligase that mediates the K48-linked ubiquitination and degradation of p53. Alteration of the nutlin proteins, which inhibit MDM2–p53 interactions, could enhance apoptosis and decrease tumorigenicity in several cell line and animal model studies [31–33]. In our data, we verified that TRIM31 stabilized the p53 protein by inducing the K63-linked ubiquitination, meanwhile, it is also suppressed the MDM2-mediated K48-linked ubiquitination of p53 by competitive inhibiting the interaction of MDM2 and p53 in breast cancer. These findings suggested TRIM31 was important for stabilizing p53 in breast cancer and it may provide a new therapeutic target for restoring and stabilizing p53 to treat cancer.

In summary, the loss of TRIM31 destabilized p53 protein to promote breast cancer progression. TRIM31 was low expressed in breast cancer and the low expression of TRIM31 was negatively correlated with the patient’s survival prognosis. Loss of TRIM31 facilitated proliferation, colony formation, migration, and invasion. Further studies showed that TRIM31 directly interacted with p53 and then induced the K63-linked ubiquitination and inhibited the K48-linked ubiquitination of p53 to stabilize and activate p53. Knockdown of p53 reversed the inhibitory effects of TRIM31 on the growth and metastasis of breast cancer cells in vitro and in vivo. Altogether, our work revealed that TRIM31 was downregulated in breast cancer, and restoring TRIM31 expression suppressed the breast cancer progression via stabilizing and activating p53. This founding indicated that restoring the expression of TRIM31 may be a new therapeutic strategy for breast cancer.

## Materials and methods

### Patient samples

Forty-six normal tissues and ninety breast cancer tissues were collected from Huaxi Biobank of West China Hospital. The histological features of all the breast cancer samples were identified by pathologists. IHC was performed on tissues fixed with 4% formaldehyde. The obtained tissues were frozen and stored in liquid nitrogen. The research was approved by the Human Experimental Ethics Committee of Huaxi Hospital, and informed consent was obtained from all patients. All samples were used for experimental research. The method follows the approved guidelines.

### Cell culture and Lentivirus transfection

The HEK293T, MCF-7, and ZR-75-30 cell lines were purchased from the American Type Culture Collection (ATCC). These cell lines have recently been authenticated by STR profiling and also were tested for mycoplasma contamination every six months and confirmed to be negative. The cells were cultured in complete DMEM or RPMI 1640 medium containing 10% fetal bovine serum (FBS) (Gibco, USA) in a humidified atmosphere of 5% CO_2_ at 37 °C. Lentivirus of shTRIM31, TRIM31-overexpression, shp53, p53-overexpression, and each negative control was obtained from Genepharm (Shanghai, China), and the shRNA sequences were listed in Table 2. The operating procedure completely followed the manufacturer’s instructions. The cell lines were treated with puromycin for 2 weeks.

**Table 2:** The sequences of primer and shRNA.

Name	Sequence
TRIM31 Forward	5′-AACCTGTCACCATCGACTGTG-3′
TRIM31 Reverse	5′-TGATTGCGTTCTTCCTTACGG-3′
P53 Forward	5′-GCTTTCCACGACGGTGAC-3′
P53 Reverse	5′-GCTCGACGCTAGGATCTGAC-3′
P21 Forward	5′-TACCCTTGTGCCTCGCTCAG-3′
P21 Reverse	5′-GAGAAGATCAGCCGGCGTTT-3′
BAX Forward	5′-TGACATGTTTTCTGACGGCAAC-3′
BAX Reverse	5′-GGAGGCTTGAGGAGTCTCACC-3′
18S Forward	5′-GATATGCTCATGTGGTGTTG-3′
18S Reverse	5′-AATCTTCTTCAGTCGCTCCA-3′
Sh-TRIM31 #1	5′-CCCAUAAUGUCAGCUUGAUCGAAGA-3′
Sh-TRIM31 #2	5′-GAGCAGAUCCAAGUCUUGCAGCAAA-3′
ShP53	5′-AAGCGAGCACTGTCCAACAAC-3′

### Plasmid and reagents

The pCMV-Flag-TRIM31, TRIM31 domain deletion mutant plasmids, pLVX-HA-p53, and the p53 domain deletion mutant plasmids were generated using standard molecular techniques. Flag-Ub, Flag-Ub-K48, Flag-Ub-K63, Flag-Ub-K48R, and Flag-Ub-K63R were kindly provided by Hongbo Hu (Sichuan University, Chengdu, China). The primary antibodies against TRIM31 (#12543–1-AP) and MDM2 (19058-1-AP) were obtained from Proteintech (Chicago, IL, USA). The primary antibodies against HA-Tag (C29F4), Flag-tag (D6W5B), p53 (1C12), p21 (12D1), bax (D2E11) were from Cell Signaling Technology (Danvers, MA, USA). The primary antibodies against ubiquitin and β-actin were purchased from Huabio (Hang Zhou, Ze Jiang, China). The Nutlin3 (548472-68-0) and PFTα (63208-82-2) were purchased from TargetMol (Shanghai, China).

### Immunohistochemistry

Breast cancer and adjacent tissues were cut at 4 μm and deparaffinization and rehydration were performed with a solution of xylene and a series of ethanol solutions. Antigens were recovered by boiling the slides in a 0.01 M sodium citrate buffer at 100 °C for 5 min. Subsequently, the slides were sealed with 3% hydrogen peroxide for 10 min and then sealed with 5% horse serum for 1 h at room temperature (RT). Immunohistochemistry staining of specimens was carried out using the primary antibodies anti-TRIM31, anti-p53, and the slides were incubated at 4 °C overnight. The secondary antibodies (Gene Tech, Shanghai, China) were used at the appropriate concentrations. Staining was performed with hematoxylin was used for counterstaining. IHC localization is scored in a semi-quantitative manner incorporating a specific stain of intensity and distribution. Moreover, IHC analysis for breast cancer tissue was performed without any information about patients. The investigators were blinded to group allocation during collection and/or analysis.

### Cell proliferation, colony formation, and transwell assay

The cell proliferation was detected by the CCK-8, the cells (1 × 10^4^) were seeded into 96-wells plates and three wells in each group (*n* = 3), then cultured overnight. The cell viability was detected by a CCK-8 (Topscience, Shanghai, China) assay. The optical density was measured at 450 nm using the ELISA plate reader (Model 550; Bio-Rad). The colony formation assay was performed to detect the cell proliferation, and the cells were plated in 24-wells plates in a number of 400 and 3-wells in each group (*n* = 3), further cultured for 2 weeks. Cells were fixed by ice-cold 4% formaldehyde solution and stained with 0.05% crystal violet solution. Numbers of visible colonies were counted using Image J. The migration and invasion abilities of breast cancer cells were evaluated by coating with or without Matrigel Transwell (Corning, USA). The 6 × 10^4^ cells in 200 μl serum-free medium were moved into the upper chamber, and containing 10% FBS medium was moved into the lower chamber, the cells on the upper surface of the membrane were erased carefully after incubation for 48 h, and the cells adhered to the lower membrane were stained with 0.05% crystal violet solution. Counted the number of migrated or invaded cells in three random fields under a microscope at ×200 magnification. The sample size was determined based on published papers and previous experience. The sample size of *n* = 3 would allow for adequate analysis to reach meaningful conclusions of the data.

### Real-time PCR assay

Total RNA from tissues or cells was isolated by using RNAiso plus reagent (Takara, Dalian China). Reverse transcription was used in the All-In-One cDNA Synthesis Kit (Bimake, USA) following the manufacturer’s protocol. The real-time PCR was performed by using 2× SYBR Green qPCR Master Mix (Bimake, USA). The 2^−ΔΔCt^ method for relative quantification of the gene expression. The sequences of primer were listed in Table 2.

### Western blot and co-IP assay

Western blotting was performed according to the standard protocol. Cell lysates were prepared in RIPA buffer (150 mM sodium chloride, 1.0% NP-40, 50 mM Tris, pH 8.0) supplemented with 1% protease inhibitors (MCE, USA). The proteins in the lysate were separated by SDS-PAGE and then transferred to a polyvinyl difluoride membrane (Millipore). Incubate the primary antibody overnight at 4 °C. The next day incubated with horseradish peroxidase-conjugated secondary antibodies (Sangon Biotech, Shanghai, China) for 1 h at RT. The membrane was developed using Immobilon^™^ Western Chemiluminescent HRP Substrate (Millipore). Proteins expression levels were quantified by Image J. For co-IP experiments, the cell lysates were prepared and incubated with suitable antibodies and Protein A/G beads (MCE, New Jersey, USA). Washing the beads with TBS buffer 3 times. The immunoprecipitates were added in 1× SDS Loading Buffer and further assay by western blot.

### In vitro binding assay

GST fusion proteins of p53 and GST protein were recombinant expressed in Escherichia coli and purified with GST-Sefinose(TM) 1 ml (Sangon Biotech, Shanghai, China) according to the manufacturer’s protocol. The His-TRIM31 and His-MDM2 protein were produced in *Escherichia coli* and purified with Ni-TED 1 ml Sefinose(TM) Column (Sangon Biotech, Shanghai, China) according to the manufacturer’s protocol. Mixing His-TRIM31 and GST-p53 protein together, followed by GST pull-down assay with GST beads and western blot assay with GST and His antibody.

### BiFC assay

P53 and MDM2 cDNA were cloned into the N- and C-terminal non-fluorescent fragments of the Venus fluorescent plasmid (pBiFC-Flag-VN173 and pBiFC-HA-VC155), respectively. PBiFC-Flag-VN173-p53 and pBiFC-HA-VC155-MDM2 plasmids were co-transfected into TRIM31 overexpressing ZR-75-30 by using Lipofectamine 3000 (Invitrogen, L3000015) according to the manufacturer’s protocol. After 48 h, the fluorescence was imaged using an Olympus fluorescence microscope.

### In vitro and in vivo ubiquitination assays

In vitro ubiquitination assays using a ubiquitination kit (Boston Biochem, MA, USA) following the manufacturer’s protocol. Briefly, preparing a mix in 1.5 ml tubes and following the volumes: 7 μl dH_2_O, 2 µl 10× reaction buffer, 2 µl 10× ubiquitin, 1 µl 20× E1 Enzyme, 2 μl 10× E2 conjugating enzyme, 0.5 μg His-TRIM31, 1 μg GST-p53. Adding 2 µl of 10× Mg^2+^-ATP solution in a final volume of 50 μL to initiate a conjugation reaction. Mixed by pipetting or gently flicking tube, and incubate for 3 h in 37 °C water bath. The reaction was terminated by 1× SDS-PAGE loading buffer. The ubiquitination of GST-p53 was detected by western blot with an anti-p53 antibody.

In vivo ubiquitination assays the ubiquitination of endogenous p53, after transfection with various plasmids for 48 h, the MCF7 cells were treated with MG132 (20 μM) for 4 h before harvest. The cell lysates were prepared and incubated with 1 μg of anti-p53 antibody overnight at 4 °C. The next day, added in 20 μl proteinA/G beads (MCE, New Jersey, USA) at 4 °C for 4 h. Washing the beads with TBS buffer 3 times. The immunoprecipitates were added in 1× SDS Loading Buffer and further assay by western blot.

### CRISPR/Cas9-mediated MDM2 knockout in breast cancer cells

Lenti-CRISPR vectors containing sgRNA targeting MDM2 were constructed and verified by DNA sequencing. The sequence of sgRNA-MDM2 was 5′-GTTGGGCCCTTCGTGAGAAT-3′. The Lenti-CRISPR vectors were transiently transfected into breast cancer cells, then the cells were treated with puromycin for 48 h. The puromycin-resistant cells were cultured in a fresh culture medium. The expression of MDM2 protein was detected by Western blotting. The cleavage of MDM2 at the target loci in breast cancer cells was detected by the T7EN1 assay using the following primers: MDM2-clev-forward, 5′-TGCTAGCATTCCTGTGACTGAG-3′; MDM2-clev-reverse: 5′-AAAGCCCTCTTCAGCTTGTGT-3′.

### Animal study

Six-week-old BALB/c nude female mice were purchased from HFK Bioscience (Beijing, China). Then randomly divided into 3 groups, 7 mice in each group (*n* = 7). The sample size was determined based on published papers and previous experience. The sample size of *n* = 7 was used to compensate for higher natural variance in vivo. To generate a xenograft tumor model, a total of 5 × 10^6^ ZR-75-30 cells stably overexpressing TRIM31, TRIM31-C36A, or empty vector were injected into nude mice. Tumor sizes were detected by Vernier calipers every 2 days. Tumor volume was calculated using the following formula: *V* (mm^3^) = 1/2*length*width^2^. On the 30th day after injection, the mice were sacrificed, and the tumors were completely isolated and measured the volume and weight of tumors. No data were excluded from our analyses. Then fixed with 4% paraformaldehyde for further study. For tumor metastasis experiments, ZR-75-30 cells stably overexpressing TRIM31 or the control vector were injected into nude mice via the tail vein. The mice were sacrificed 50 days after the injection, the number of nodules on the lung surface was counted, and the lungs were fixed with 4% paraformaldehyde for further hematoxylin and eosin (HE) staining assays.

To verify that p53 rescues the antitumor effect of TRIM31 in vivo, ZR-75-30 cells stably overexpressing TRIM31, empty vector, TRIM31 + sh-p53, and empty vector + sh-p53 were digested and resuspended in PBS. The mice were randomly divided into four groups, 7 mice in each group (*n* = 7). Mice were anesthetized by intraperitoneal injection of 4% chlorine hydrate (Sigma-Aldrich), and the dose was 0.2 mL/20 g weight. A small incision was made between the fourth nipple and the midline to reveal the mammary gland. A total of 5 × 10^6^ cells were completely injected into the fat pad of the mammary gland in the groin without leakage. The incisions were sutured, and the mice were observed until they regained consciousness. The volumes of the tumors were measured for 3 days with a Vernier caliper. Finally, the mice were sacrificed, and the xenografts and lung tissues were harvested, measured, and fixed with 4% paraformaldehyde for further HE staining. All experimental protocols were approved by the Teaching and Research Living Objects Committee of Sichuan University.

### Statistical analysis

The data were statistically analyzed with the SPSS software package (SPSS, IL, USA) and GraphPad Prism software (GraphPad, CA, USA). Differences between the two groups were assessed by Student’s *t* test, and differences between multiple groups were assessed by one-way ANOVA. The correlations were assessed according to the Pearson coefficient. The data are shown as the mean ± SD of at least three independent experiments. * indicates *P* < 0.05, ** indicates *P* < 0.01, and *** indicates *P* < 0.001.

## Supplementary information

Supplementary Figures and Tables

Supplementary Figure Legends

## Data Availability

The datasets used and/or analyzed during the current study are available from the corresponding author on reasonable request.
