# Supplementary material for: Loss of TRIM31 promotes breast cancer progression through regulating K48- and K63-linked ubiquitination of p53
Source: Cell Death Dis. 2021 Oct 14;12(10):945. doi: 10.1038/s41419-021-04208-3 (PMC8516922; doi:10.1038/s41419-021-04208-3)
Supplement: Supplementary file 1 — Supplementary Figures and Tables [file 41419_2021_4208_MOESM1_ESM.pdf]

# Supplementary Fig. S1

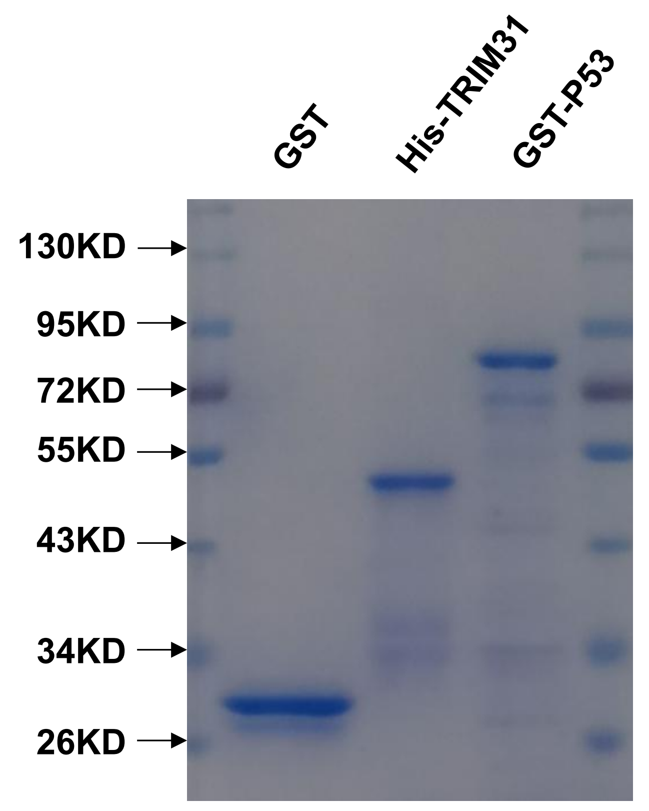


# Supplementary Fig. S2

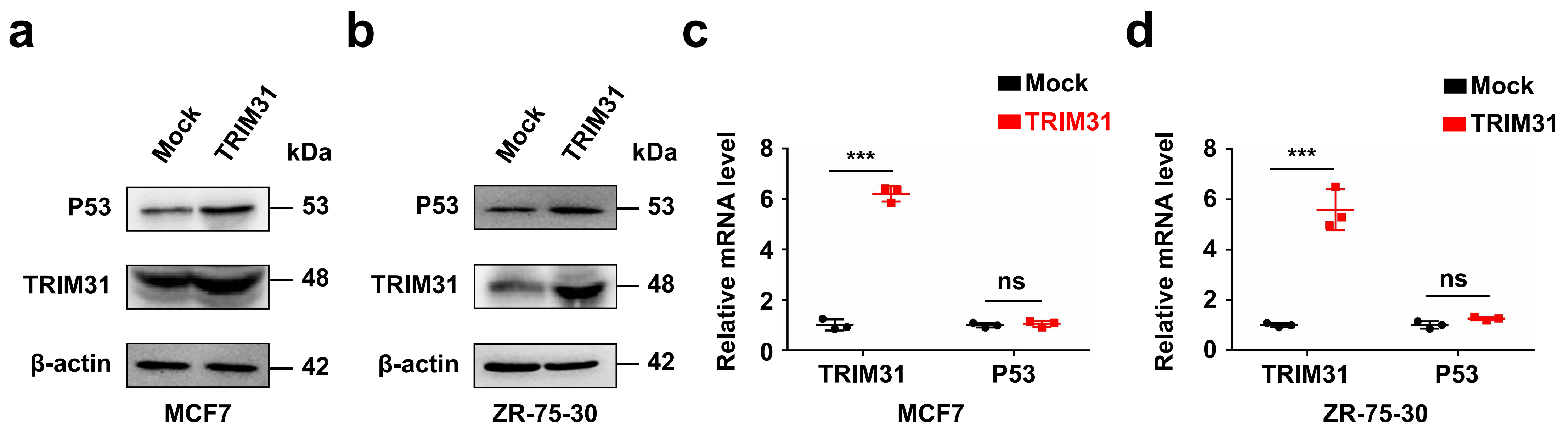


# Supplementary Fig. S3

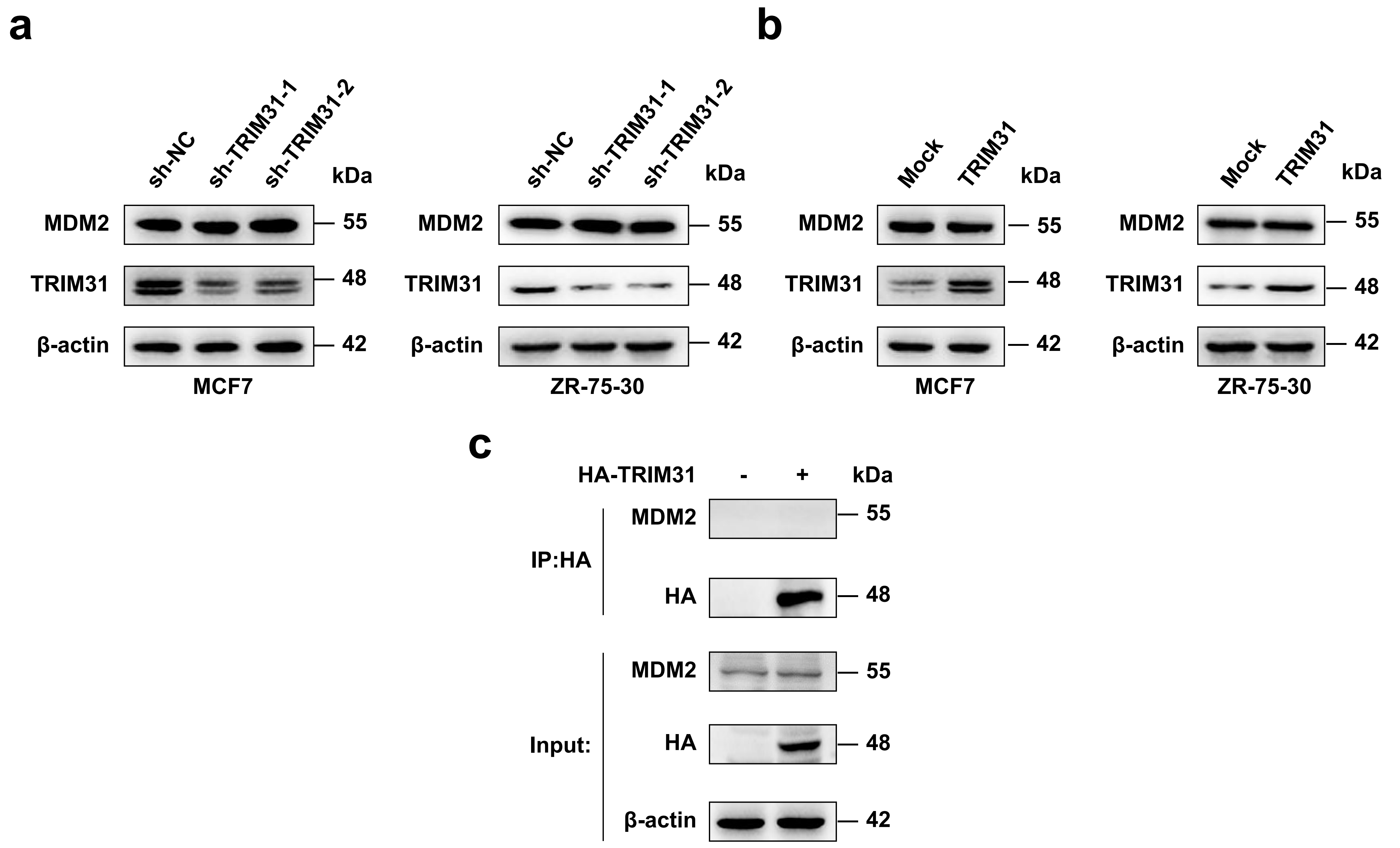


# Supplementary Fig. S4

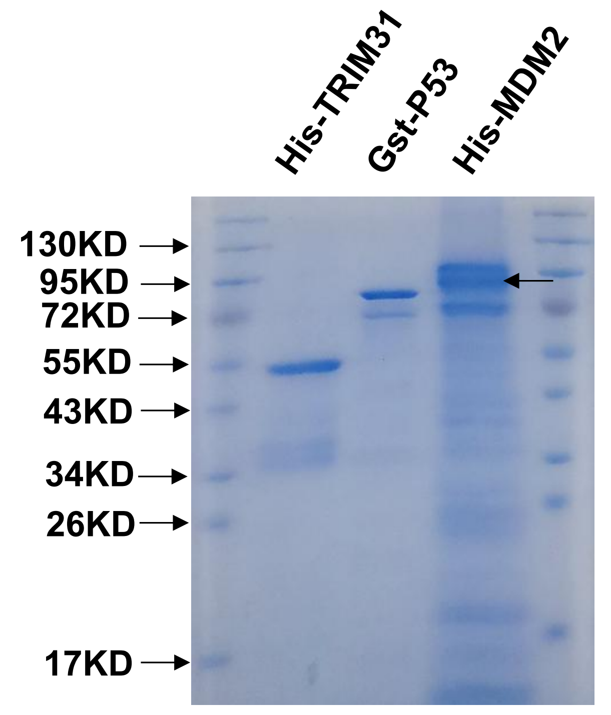

# Supplementary Fig. S5

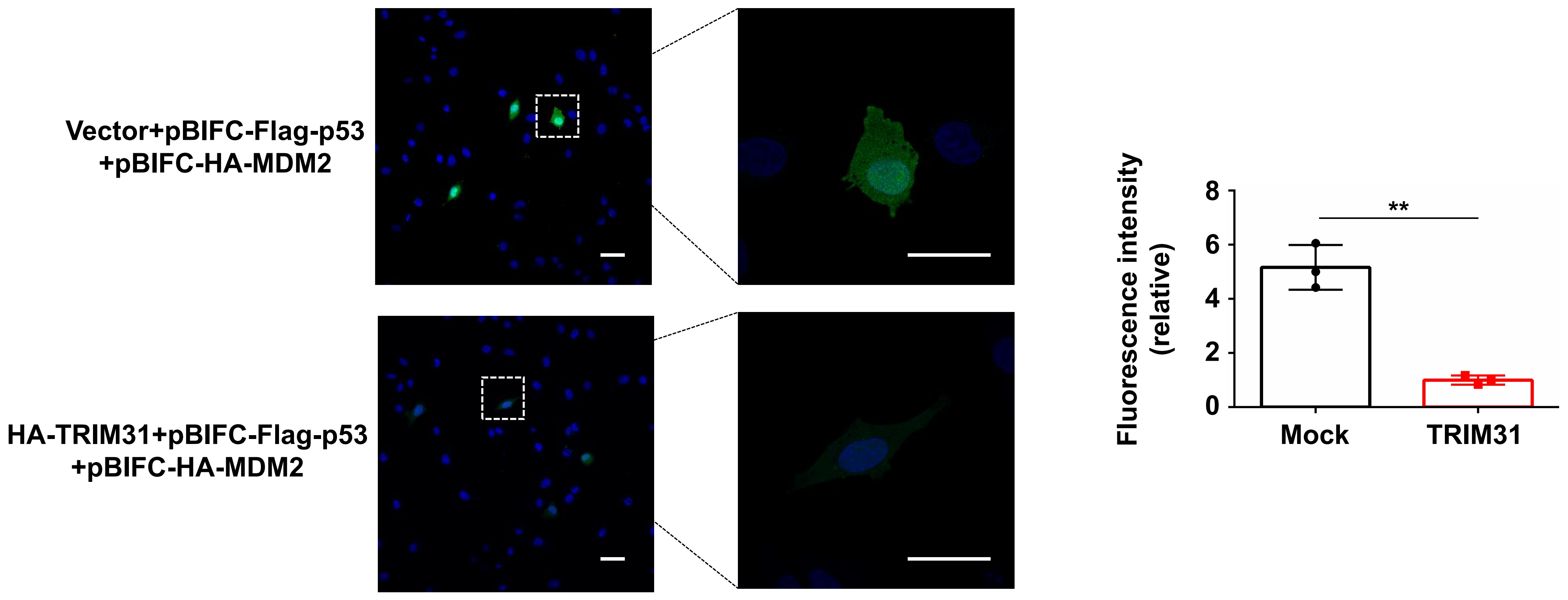


# Supplementary Fig. S6

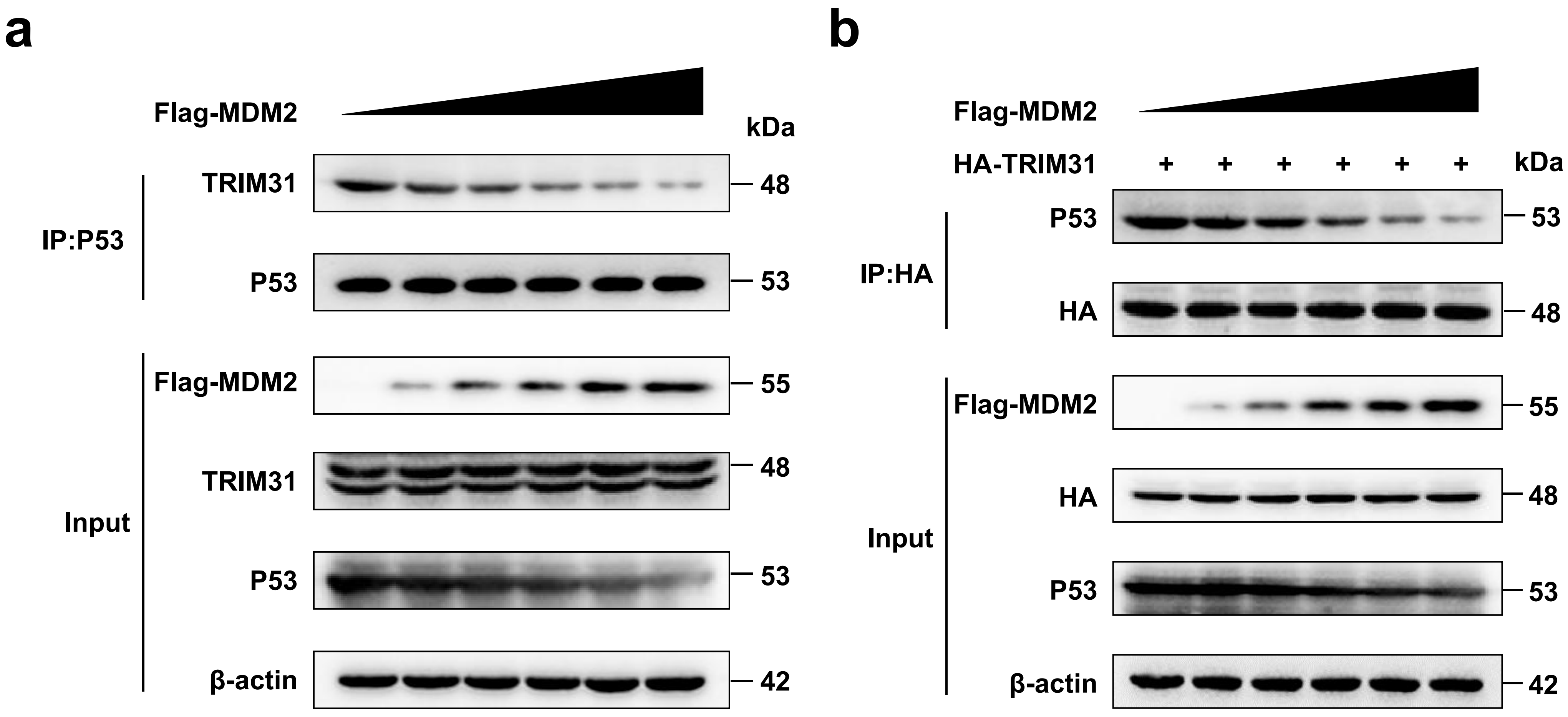


# Supplementary Fig. S7

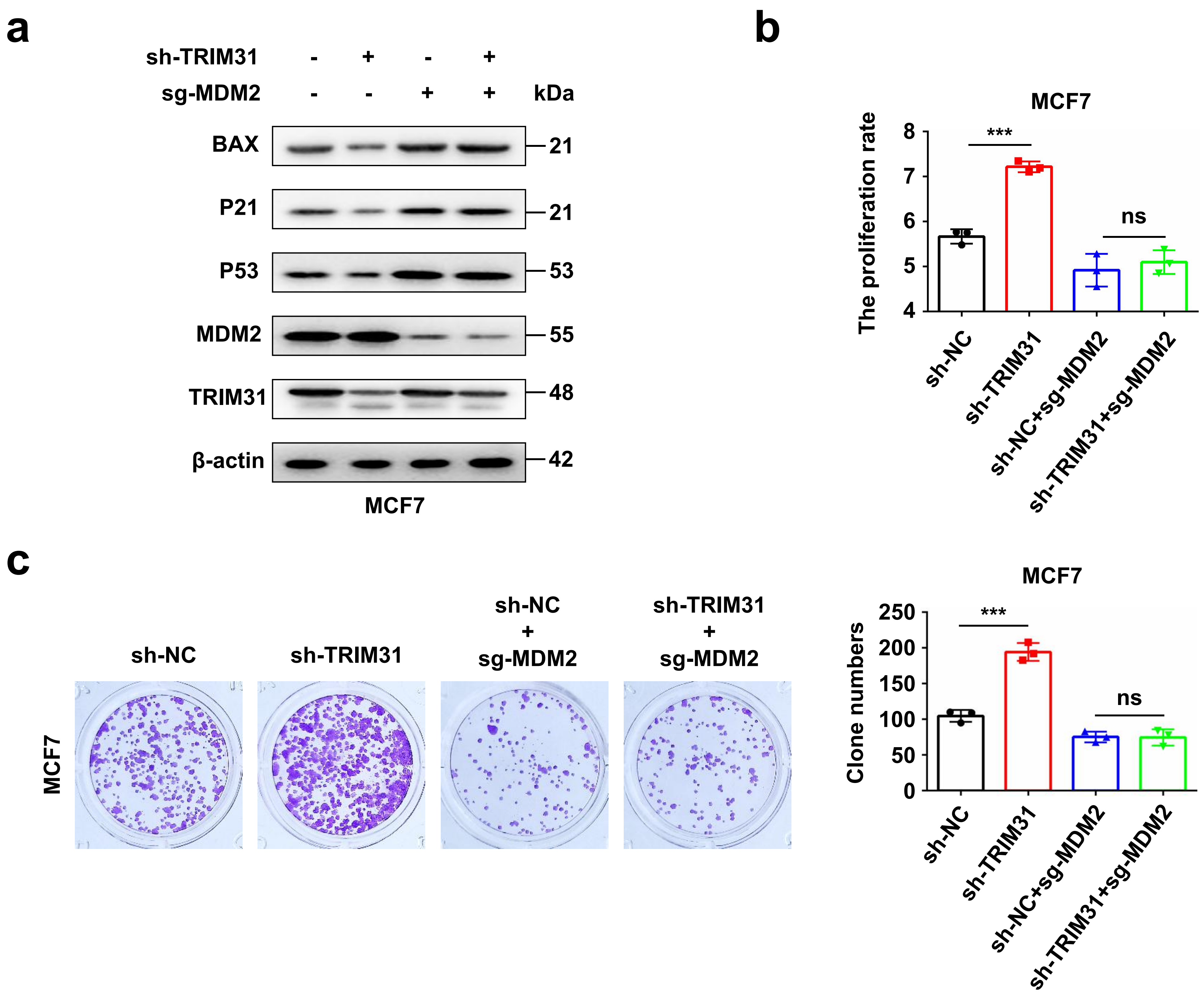

# Supplementary Fig. S8

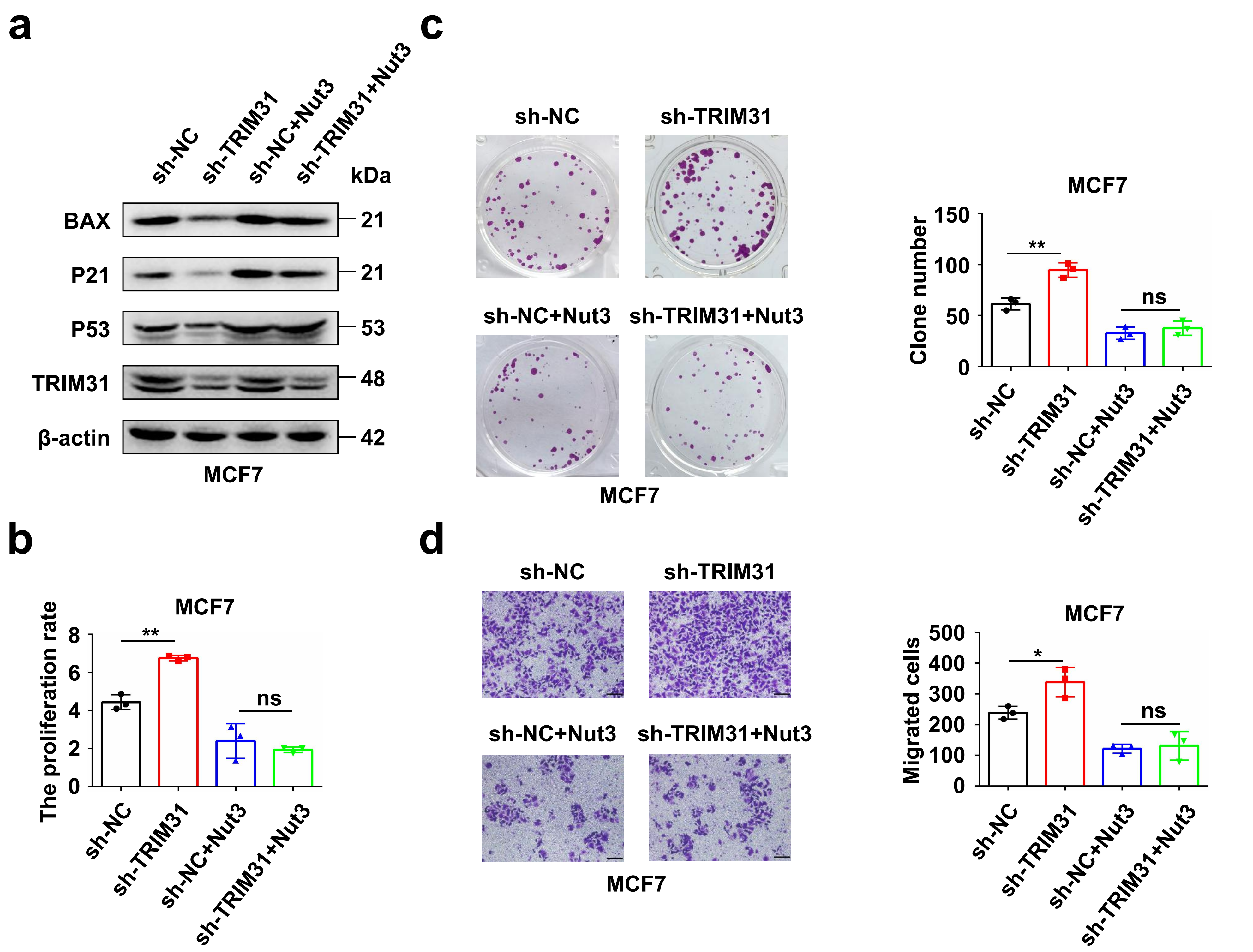


# Supplementary Fig. S9

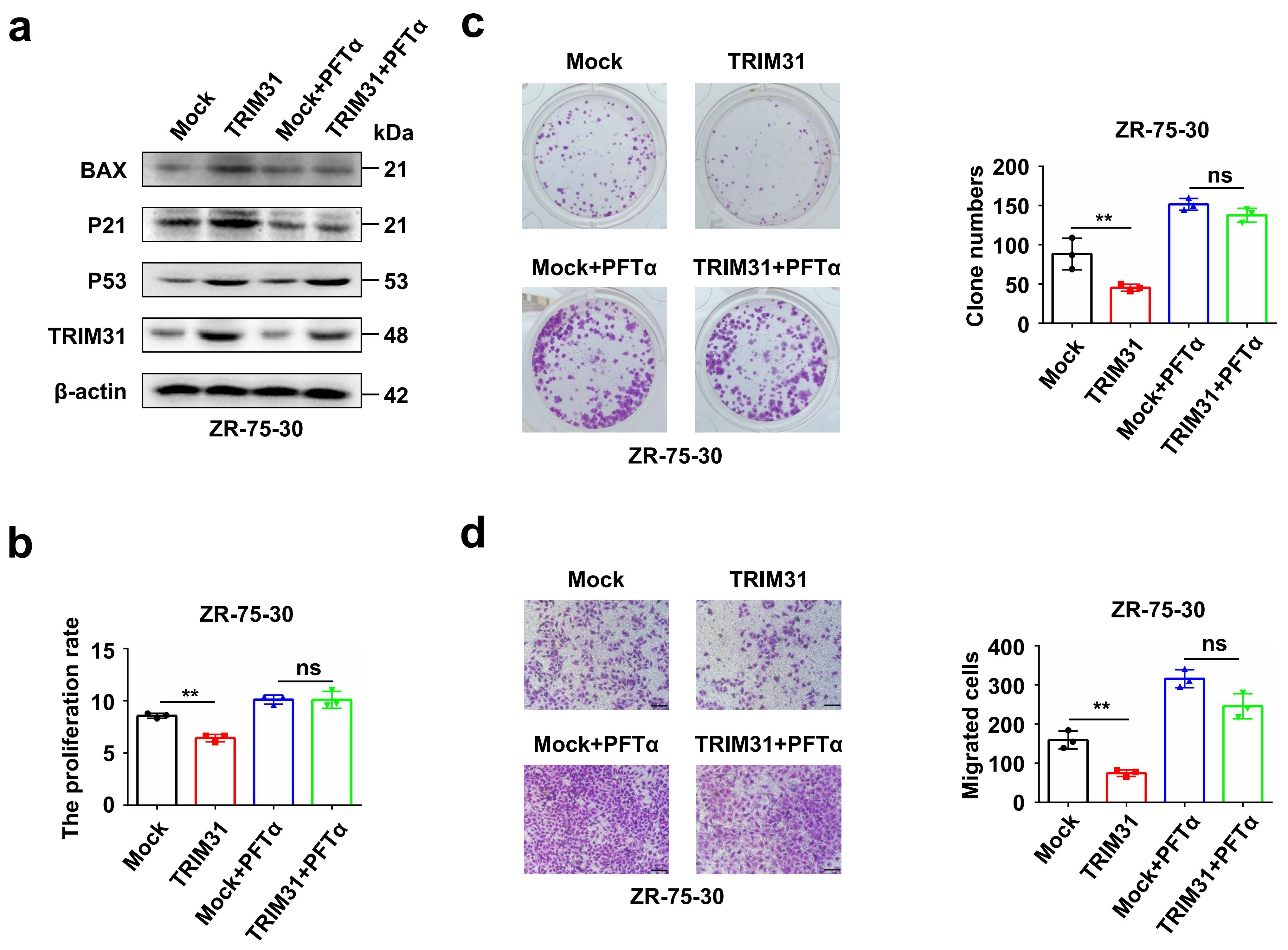


# Supplementary Fig. S10

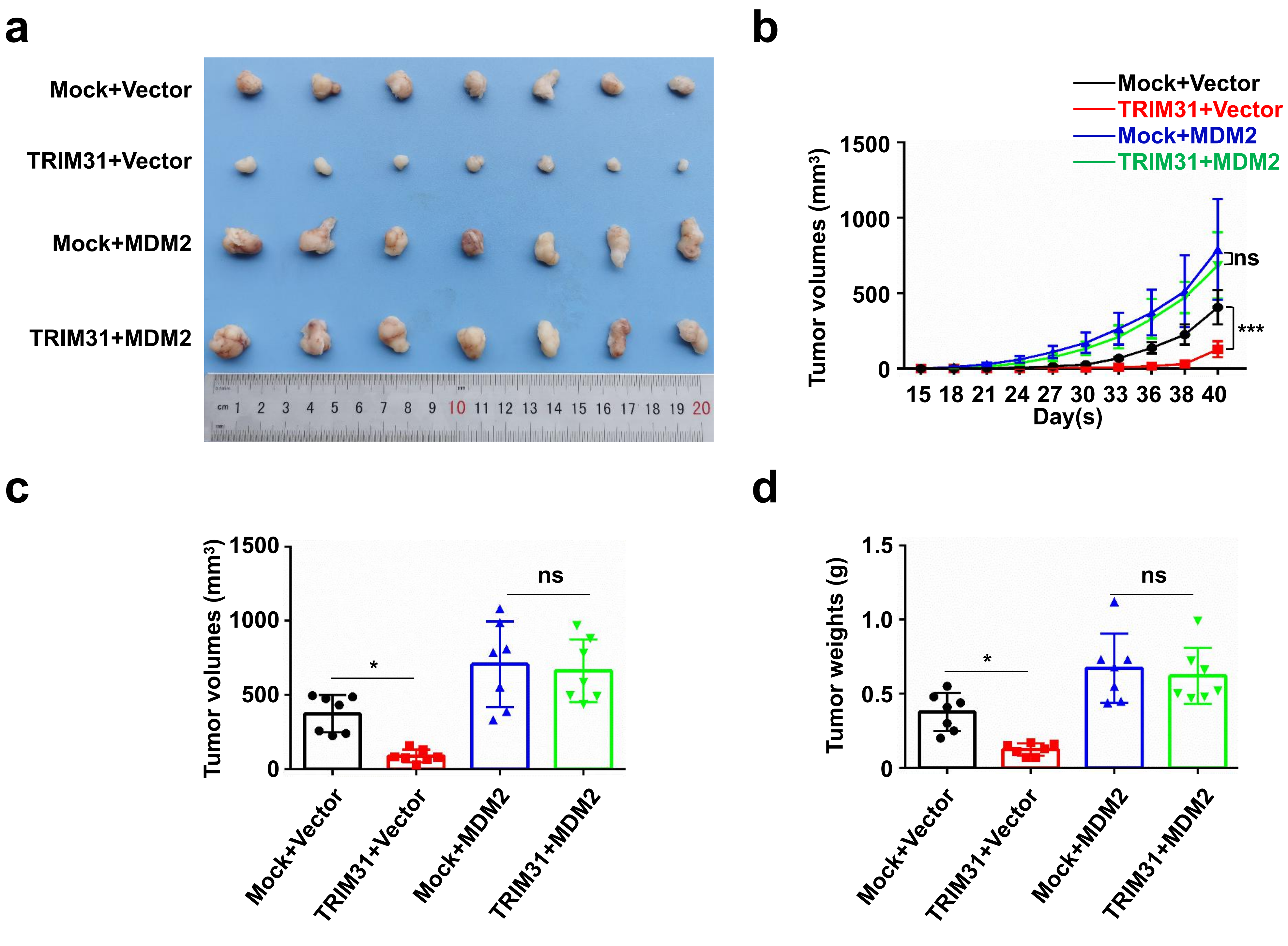

Supplementary Table S1:The up and down-regulated proteins in response to TRIM31 knockdown in MCF7 cells

| Gene.name | Number.of | Unique.pe | Sequence. | MS.MS.cou | Q.value | Score | LFQ.intensit | LFQ.intensit | LFQ.intensit | LFQ.intensit | iBAQ | Symbol | Rank | MCF7-1.1 | MCF7-NC.1 | im..MCF7- | im..MCF7- | MCF7-1 | MCF7-NC | fc..MCF7-1--MCF7-NC |
|---|---|---|---|---|---|---|---|---|---|---|---|---|---|---|---|---|---|---|---|---|
| PPP6R2 | 8 | 2 | 2.2 | 2 | 0.000435 | 3.4069 | 0 | 0 | 0 | 499330000 | 14938000 | PPP6R2 | 841 | 1870511.94 | 499330000 | TRUE | FALSE | 1870511.94 | 499330000 | 0.003746 |
| TMSB10 | 2 | 2 | 50 | 3 | 0 | 15.145 | 0 | 0 | 0 | 798450000 | 1.24E+09 | TMSB10 | 11 | 3504422.281 | 798450000 | TRUE | FALSE | 3504422.281 | 798450000 | 0.004389 |
| RPS24 | 9 | 5 | 40.5 | 5 | 0 | 13.344 | 90614000 | 0 | 0 | 458280000 | 98940000 | RPS24 | 230 | 3281493.782 | 458280000 | TRUE | FALSE | 3281493.782 | 458280000 | 0.00716 |
| ARFGEF2;A | 4 | 4 | 2.2 | 2 | 0.000472 | 4.2618 | 29897000 | 0 | 0 | 319450000 | 4512400 | ARFGEF2 | 1504 | 3335263.116 | 319450000 | TRUE | FALSE | 3335263.116 | 319450000 | 0.010441 |
| SPINT2;IM | 8 | 2 | 14.4 | 2 | 0.000797 | 2.6703 | 0 | 0 | 0 | 148880000 | 20276000 | SPINT2 | 707 | 1630307.222 | 148880000 | TRUE | FALSE | 1630307.222 | 148880000 | 0.01095 |
| FAU | 3 | 2 | 17.3 | 13 | 0 | 5.5918 | 71237000 | 70667000 | 0 | 246060000 | 92540000 | FAU | 244 | 3113324.751 | 246060000 | TRUE | FALSE | 3113324.751 | 246060000 | 0.012653 |
| RABGGTA | 2 | 2 | 4 | 2 | 0.001148 | 2.3356 | 0 | 0 | 0 | 228920000 | 4625400 | RABGGTA | 1479 | 2944677.72 | 228920000 | TRUE | FALSE | 2944677.72 | 228920000 | 0.012863 |
| TMED4 | 4 | 2 | 17.5 | 2 | 0.000807 | 2.7901 | 0 | 0 | 0 | 93645000 | 7203100 | TMED4 | 1237 | 2246483.689 | 93645000 | TRUE | FALSE | 2246483.689 | 93645000 | 0.023989 |
| TRIM31 | 1 | 2 | 27.2 | 5 | 0.000469 | 4.2145 | 0 | 0 | 0 | 32518000 | 35901000 | NDUFA4 | 477 | 1335919.992 | 32518000 | TRUE | FALSE | 1335919.992 | 32518000 | 0.041082 |
| SYNGR2 | 6 | 3 | 13.4 | 4 | 0 | 4.6909 | 7974600 | 0 | 0 | 58569000 | 14331000 | SYNGR2 | 861 | 2803956.332 | 58569000 | TRUE | FALSE | 2803956.332 | 58569000 | 0.047874 |
| TUBB8 | 3 | 2 | 24.5 | 6 | 0 | 61.623 | 85742000 | 98145000 | 0 | 62898000 | 13568000 | TUBB8 | 893 | 3038424.98 | 62898000 | TRUE | FALSE | 3038424.98 | 62898000 | 0.048307 |
| TRANK1 | 3 | 2 | 0.5 | 2 | 0.006511 | 1.5407 | 0 | 0 | 0 | 160860000 | 1963400 | TRANK1 | 1974 | 7894958.145 | 160860000 | TRUE | FALSE | 7894958.145 | 160860000 | 0.04908 |
| SAP18 | 4 | 2 | 23.7 | 4 | 0.004963 | 1.7604 | 28670000 | 26202000 | 0 | 37116000 | 22817000 | SAP18 | 648 | 1865819.295 | 37116000 | TRUE | FALSE | 1865819.295 | 37116000 | 0.05027 |
| SEC13 | 6 | 5 | 26.4 | 8 | 0 | 59.784 | 35216000 | 39838000 | 0 | 33154000 | 7670900 | SEC13 | 1196 | 1936689.34 | 33154000 | TRUE | FALSE | 1936689.34 | 33154000 | 0.058415 |
| SNRPG;SNR | 5 | 2 | 29.7 | 8 | 0 | 7.6473 | 110400000 | 125800000 | 0 | 92634000 | 165640000 | SNRPG | 148 | 5431764.415 | 92634000 | TRUE | FALSE | 5431764.415 | 92634000 | 0.058637 |
| UFM1 | 2 | 2 | 28.4 | 5 | 0.000465 | 4.1298 | 0 | 0 | 0 | 64013000 | 27029000 | UFM1 | 583 | 3912627.004 | 64013000 | TRUE | FALSE | 3912627.004 | 64013000 | 0.061122 |
| SEC63 | 1 | 5 | 7.2 | 8 | 0 | 10.508 | 68769000 | 86865000 | 0 | 58577000 | 5681200 | SEC63 | 1355 | 3688238.288 | 58577000 | TRUE | FALSE | 3688238.288 | 58577000 | 0.062964 |
| GPRC5A | 3 | 2 | 4.5 | 2 | 0.003663 | 1.9788 | 0 | 0 | 0 | 34526000 | 13442000 | GPRC5A | 898 | 2455790.355 | 34526000 | TRUE | FALSE | 2455790.355 | 34526000 | 0.071129 |
| HKDC1 | 3 | 3 | 5.8 | 3 | 0.000441 | 3.5184 | 0 | 0 | 0 | 37898000 | 581290 | HKDC1 | 2532 | 2793830.925 | 37898000 | TRUE | FALSE | 2793830.925 | 37898000 | 0.07372 |
| GUK1 | 9 | 3 | 24.1 | 3 | 0.000463 | 4.0576 | 0 | 0 | 0 | 29360000 | 3013900 | GUK1 | 1749 | 2424555.148 | 29360000 | TRUE | FALSE | 2424555.148 | 29360000 | 0.08258 |
| UBE2D3;UE | 17 | 2 | 12.2 | 2 | 0.000818 | 2.8969 | 54741000 | 0 | 0 | 56740000 | 41491000 | UBE2D3 | 437 | 4774745.73 | 56740000 | TRUE | FALSE | 4774745.73 | 56740000 | 0.084151 |
| PSMF1 | 5 | 2 | 10.3 | 6 | 0 | 6.0217 | 13351000 | 14477000 | 0 | 26606000 | 5358000 | PSMF1 | 1380 | 2272814.416 | 26606000 | TRUE | FALSE | 2272814.416 | 26606000 | 0.085425 |
| POFUT1 | 2 | 8 | 30.7 | 13 | 0 | 20.555 | 67984000 | 101800000 | 0 | 35112000 | 10643000 | POFUT1 | 1013.5 | 3219208.887 | 35112000 | TRUE | FALSE | 3219208.887 | 35112000 | 0.091684 |
| RPP30 | 4 | 2 | 11.9 | 4 | 0.004945 | 1.7468 | 0 | 0 | 0 | 37969000 | 11434000 | RPP30 | 977 | 3532253.071 | 37969000 | TRUE | FALSE | 3532253.071 | 37969000 | 0.09303 |
| UBE2I | 4 | 2 | 32.9 | 7 | 0 | 9.5079 | 31538000 | 29766000 | 0 | 59920000 | 32505000 | UBE2I | 509 | 5708025.329 | 59920000 | TRUE | FALSE | 5708025.329 | 59920000 | 0.095261 |
| GNG5 | 1 | 2 | 27.9 | 2 | 0 | 4.7259 | 0 | 0 | 0 | 40027000 | 17880000 | GNG5 | 754 | 3844380.953 | 40027000 | TRUE | FALSE | 3844380.953 | 40027000 | 0.096045 |
| ERC1 | 12 | 2 | 3 | 3 | 0.000433 | 3.3705 | 0 | 20740000 | 0 | 36962000 | 1819500 | ERC1 | 2013 | 3658407.888 | 36962000 | TRUE | FALSE | 3658407.888 | 36962000 | 0.098978 |
| FMR1 | 18 | 4 | 7.3 | 3 | 0 | 4.7469 | 22233000 | 23449000 | 0 | 23459000 | 2315700 | FMR1 | 1887 | 2428592.262 | 23459000 | TRUE | FALSE | 2428592.262 | 23459000 | 0.103525 |
| RTN1 | 5 | 2 | 8.3 | 8 | 0.002216 | 2.0678 | 0 | 0 | 0 | 121130000 | 27498000 | RTN1 | 577 | 13066881.25 | 121130000 | TRUE | FALSE | 13066881.25 | 121130000 | 0.107875 |
| DNAJC3 | 2 | 5 | 12.8 | 4 | 0 | 4.7295 | 25094000 | 22063000 | 0 | 25981000 | 4201200 | DNAJC3 | 1544 | 3010009.611 | 25981000 | TRUE | FALSE | 3010009.611 | 25981000 | 0.115854 |
| ARFGAP1 | 13 | 3 | 8.2 | 3 | 0.000448 | 3.6803 | 0 | 0 | 0 | 17763000 | 1901600 | ARFGAP1 | 1987 | 2132001.163 | 17763000 | TRUE | FALSE | 2132001.163 | 17763000 | 0.120025 |
| CD9 | 5 | 2 | 10.7 | 4 | 0.000436 | 3.4413 | 0 | 24618000 | 0 | 31074000 | 23552000 | CD9 | 631 | 3822533.712 | 31074000 | TRUE | FALSE | 3822533.712 | 31074000 | 0.123014 |
| CD63 | 7 | 2 | 13.5 | 2 | 0 | 6.9266 | 0 | 0 | 0 | 25703000 | 30951000 | CD63 | 523 | 3321682.491 | 25703000 | TRUE | FALSE | 3321682.491 | 25703000 | 0.129233 |
| ARPC5L | 1 | 2 | 16.3 | 5 | 0 | 5.0136 | 0 | 0 | 0 | 15289000 | 4592600 | ARPC5L | 1489 | 1988489.929 | 15289000 | TRUE | FALSE | 1988489.929 | 15289000 | 0.13006 |
| PTCD3 | 3 | 3 | 6 | 5 | 0 | 6.3198 | 0 | 0 | 0 | 15582000 | 1507000 | PTCD3 | 2113 | 2189150.67 | 15582000 | TRUE | FALSE | 2189150.67 | 15582000 | 0.140492 |
| BAG3 | 2 | 8 | 21 | 11 | 0 | 13.741 | 25405000 | 24292000 | 0 | 16842000 | 1955300 | BAG3 | 1975 | 2385041.977 | 16842000 | TRUE | FALSE | 2385041.977 | 16842000 | 0.141613 |
| GREB1L | 4 | 2 | 2.1 | 1 | 0.005889 | 1.6448 | 0 | 0 | 0 | 15096000 | 511060 | GREB1L | 2574 | 2138561.655 | 15096000 | TRUE | FALSE | 2138561.655 | 15096000 | 0.141664 |
| CLTB | 3 | 5 | 19 | 7 | 0 | 5.1822 | 27068000 | 19110000 | 0 | 14415000 | 6705800 | CLTB | 1273 | 2141460.879 | 14415000 | TRUE | FALSE | 2141460.879 | 14415000 | 0.148558 |
| HDAC2 | 11 | 3 | 17.5 | 3 | 0.000436 | 3.4435 | 17626000 | 14604000 | 0 | 18794000 | 3157400 | HDAC2 | 1722 | 2794211.708 | 18794000 | TRUE | FALSE | 2794211.708 | 18794000 | 0.148676 |
| PLXND1;PL | 9 | 2 | 1 | 4 | 0.00523 | 1.6907 | 0 | 0 | 0 | 22088000 | 689680 | PLXND1 | 2469 | 3300501.487 | 22088000 | TRUE | FALSE | 3300501.487 | 22088000 | 0.149425 |
| MMAB | 5 | 2 | 11.6 | 2 | 0.0008 | 2.7227 | 0 | 0 | 0 | 19695000 | 1761400 | MMAB | 2031 | 2960548.801 | 19695000 | TRUE | FALSE | 2960548.801 | 19695000 | 0.15032 |
| ARL6IP5 | 4 | 3 | 14.4 | 4 | 0 | 6.5396 | 22758000 | 35773000 | 0 | 18487000 | 16378000 | ARL6IP5 | 800 | 2816423.649 | 18487000 | TRUE | FALSE | 2816423.649 | 18487000 | 0.152346 |
| TBCD | 9 | 7 | 7.2 | 6 | 0 | 14.986 | 31676000 | 26651000 | 0 | 20848000 | 1440800 | TBCD | 2139 | 3219743.445 | 20848000 | TRUE | FALSE | 3219743.445 | 20848000 | 0.154439 |
| TIMM8A | 1 | 2 | 22.7 | 6 | 0 | 6.5789 | 36519000 | 46539000 | 0 | 17051000 | 20613000 | TIMM8A | 696.5 | 2711713.879 | 17051000 | TRUE | FALSE | 2711713.879 | 17051000 | 0.159035 |
| IGBP1 | 1 | 4 | 17.1 | 5 | 0 | 7.6713 | 8739000 | 10222000 | 0 | 14897000 | 1775900 | IGBP1 | 2024 | 2386204.858 | 14897000 | TRUE | FALSE | 2386204.858 | 14897000 | 0.16018 |
| SLC22A18 | 4 | 3 | 9.5 | 2 | 0.00045 | 3.756 | 0 | 0 | 0 | 20069000 | 2520000 | SLC22A18 | 1827 | 3240336.106 | 20069000 | TRUE | FALSE | 3240336.106 | 20069000 | 0.16146 |
| PKP2 | 3 | 2 | 2.3 | 2 | 0.00043 | 3.3067 | 0 | 0 | 0 | 17001000 | 252270 | PKP2 | 2760 | 2767692.866 | 17001000 | TRUE | FALSE | 2767692.866 | 17001000 | 0.162796 |
| DHFR | 3 | 2 | 19.3 | 6 | 0 | 7.3323 | 18504000 | 16442000 | 0 | 17313000 | 6203200 | DHFR | 1315 | 2989244.443 | 17313000 | TRUE | FALSE | 2989244.443 | 17313000 | 0.172659 |
| EIF2B4 | 7 | 4 | 9.2 | 6 | 0 | 7.1136 | 9988200 | 10204000 | 0 | 10334000 | 1454200 | EIF2B4 | 2132 | 1838011.926 | 10334000 | TRUE | FALSE | 1838011.926 | 10334000 | 0.177861 |
| SCAMP3 | 2 | 6 | 22.5 | 11 | 0 | 35.146 | 59207000 | 38155000 | 0 | 19712000 | 11253000 | SCAMP3 | 984.5 | 3525801.546 | 19712000 | TRUE | FALSE | 3525801.546 | 19712000 | 0.178866 |
| SEC62 | 3 | 2 | 24.7 | 3 | 0.000433 | 3.3492 | 19591000 | 16711000 | 0 | 20939000 | 9503700 | SEC62 | 1073 | 3895538.159 | 20939000 | TRUE | FALSE | 3895538.159 | 20939000 | 0.186042 |
| CSTF3 | 2 | 2 | 3.3 | 3 | 0.000463 | 4.0457 | 0 | 0 | 0 | 20213000 | 873940 | CSTF3 | 2364 | 3899896.784 | 20213000 | TRUE | FALSE | 3899896.784 | 20213000 | 0.19294 |
| AKAP8 | 1 | 2 | 4.2 | 4 | 0.000469 | 4.2308 | 6354500 | 6259800 | 0 | 6484000 | 709970 | AKAP8 | 2457 | 1268892.198 | 6484000 | TRUE | FALSE | 1268892.198 | 6484000 | 0.195696 |
| XPNPEP1 | 7 | 6 | 13.2 | 6 | 0 | 9.9242 | 7489900 | 7950900 | 0 | 2.40E+07 | 1076800 | XPNPEP1 | 2279 | 4742629.793 | 2.40E+07 | TRUE | FALSE | 4742629.793 | 2.40E+07 | 0.19761 |
| RBX1 | 1 | 2 | 24.1 | 1 | 0.000461 | 4.0248 | 0 | 0 | 0 | 11962000 | 9971000 | RBX1 | 1047 | 2373293.121 | 11962000 | TRUE | FALSE | 2373293.121 | 11962000 | 0.198403 |
| DDX24 | 6 | 3 | 4.5 | 7 | 0 | 7.688 | 19423000 | 17220000 | 0 | 19025000 | 1266600 | DDX24 | 2195 | 3843039.883 | 19025000 | TRUE | FALSE | 3843039.883 | 19025000 | 0.201999 |
| NUDT2 | 1 | 5 | 38.1 | 8 | 0 | 7.755 | 25799000 | 33359000 | 0 | 17642000 | 7649500 | NUDT2 | 1198 | 3640746.719 | 17642000 | TRUE | FALSE | 3640746.719 | 17642000 | 0.206368 |
| PPT1 | 10 | 2 | 7.9 | 2 | 0 | 5.72 | 0 | 0 | 0 | 18331000 | 2122900 | PPT1 | 1934 | 3818798.991 | 18331000 | TRUE | FALSE | 3818798.991 | 18331000 | 0.208325 |
| TGM2 | 6 | 23 | 35.7 | 42 | 0 | 75.459 | 733480000 | 29152000 | 0 | 9533600 | 22814000 | TGM2 | 649 | 2095842.042 | 9533600 | TRUE | FALSE | 2095842.042 | 9533600 | 0.219837 |
| NXN | 4 | 3 | 11.7 | 2 | 0 | 6.4226 | 0 | 0 | 0 | 16165000 | 870970 | NXN | 2368 | 3620747.596 | 16165000 | TRUE | FALSE | 3620747.596 | 16165000 | 0.223987 |
| MRPS18B | 2 | 3 | 10.5 | 3 | 0.00044 | 3.5099 | 11132000 | 0 | 0 | 18863000 | 3372300 | MRPS18B | 1680 | 4242119.039 | 18863000 | TRUE | FALSE | 4242119.039 | 18863000 | 0.224891 |
| FAR1 | 2 | 2 | 7.8 | 3 | 0 | 4.891 | 0 | 0 | 0 | 22593000 | 2127600 | FAR1 | 1932 | 5170175.909 | 22593000 | TRUE | FALSE | 5170175.909 | 22593000 | 0.22884 |
| CCDC124 | 2 | 5 | 30 | 6 | 0 | 18.697 | 30283000 | 24944000 | 0 | 17115000 | 6386300 | CCDC124 | 1299 | 3959018.115 | 17115000 | TRUE | FALSE | 3959018.115 | 17115000 | 0.231319 |
| TPM1 | 9 | 2 | 20 | 3 | 0 | 7.1318 | 0 | 0 | 0 | 9138100 | 957020 | TPM1 | 2326 | 2122684.245 | 9138100 | TRUE | FALSE | 2122684.245 | 9138100 | 0.232289 |
| XAB2 | 1 | 2 | 2.5 | 4 | 0.000433 | 3.35 | 0 | 4761500 | 0 | 8117900 | 285540 | XAB2 | 2735 | 1917589.677 | 8117900 | TRUE | FALSE | 1917589.677 | 8117900 | 0.236217 |
| TSPO | 2 | 2 | 15 | 0 | 0.009735 | 1.3715 | 0 | 0 | 0 | 38194000 | 22859000 | TSPO | 646 | 9095815.202 | 38194000 | TRUE | FALSE | 9095815.202 | 38194000 | 0.238148 |
| PPIC | 1 | 2 | 9.9 | 6 | 0.000452 | 3.8022 | 0 | 0 | 0 | 20998000 | 4293000 | PPIC | 1529.5 | 5068913.619 | 20998000 | TRUE | FALSE | 5068913.619 | 20998000 | 0.2414 |
| DNAJB12 | 4 | 3 | 9.3 | 3 | 0 | 6.1336 | 6865100 | 5508700 | 0 | 5948100 | 886070 | DNAJB12 | 2360 | 1443915.581 | 5948100 | TRUE | FALSE | 1443915.581 | 5948100 | 0.242752 |
| CNN3 | 4 | 6 | 27.6 | 15 | 0 | 22.979 | 67810000 | 65239000 | 0 | 13395000 | 10695000 | CNN3 | 1010 | 3367232.191 | 13395000 | TRUE | FALSE | 3367232.191 | 13395000 | 0.25138 |
| ENSA | 16 | 3 | 28.6 | 10 | 0 | 10.106 | 25387000 | 22236000 | 0 | 14850000 | 7569700 | ENSA | 1207 | 3751760.258 | 14850000 | TRUE | FALSE | 3751760.258 | 14850000 | 0.252644 |
| LSM8 | 3 | 2 | 29.9 | 1 | 0.000428 | 3.283 | 0 | 0 | 0 | 10275000 | 6211100 | LSM8 | 1313 | 2600301.979 | 10275000 | TRUE | FALSE | 2600301.979 | 10275000 | 0.253071 |
| GIPC1 | 4 | 2 | 8.7 | 6 | 0 | 8.669 | 5444500 | 6158200 | 0 | 6431000 | 1207900 | GIPC1 | 2223.5 | 1658281.071 | 6431000 | TRUE | FALSE | 1658281.071 | 6431000 | 0.257857 |
| GTF2F1 | 4 | 6 | 17.6 | 8 | 0 | 13.776 | 9345600 | 9214000 | 0 | 12697000 | 1477900 | GTF2F1 | 2122 | 3299374.71 | 12697000 | TRUE | FALSE | 3299374.71 | 12697000 | 0.259855 |
| SH3GLB2 | 8 | 2 | 8 | 3 | 0 | 4.3985 | 9187100 | 8908700 | 0 | 12091000 | 2162200 | SH3GLB2 | 1924 | 3145368.041 | 12091000 | TRUE | FALSE | 3145368.041 | 12091000 | 0.260141 |
| TMA7;hCG_ | 2 | 2 | 29.7 | 3 | 0.000805 | 2.7638 | 21265000 | 20297000 | 0 | 18289000 | 31379000 | TMA7 | 518 | 4900677.388 | 18289000 | TRUE | FALSE | 4900677.388 | 18289000 | 0.267958 |
| GSTT1;Em: | 15 | 2 | 11.5 | 3 | 0 | 4.6394 | 10620000 | 10295000 | 0 | 25589000 | 5256400 | GSTT1 | 1393 | 6924439.76 | 25589000 | TRUE | FALSE | 6924439.76 | 25589000 | 0.270602 |
| NUDCD2 | 2 | 3 | 29.5 | 6 | 0 | 7.2643 | 11973000 | 15661000 | 0 | 12876000 | 6425500 | NUDCD2 | 1295 | 3500070.423 | 12876000 | TRUE | FALSE | 3500070.423 | 12876000 | 0.271829 |
| LRPAP1 | 1 | 3 | 7 | 2 | 0.00045 | 3.7566 | 0 | 0 | 10424000 | 38068000 | 2368700 | LRPAP1 | 1872 | 10424000 | 38068000 | FALSE | FALSE | 10424000 | 38068000 | 0.273826 |
| GPX4 | 10 | 2 | 16.8 | 3 | 0 | 5.6111 | 0 | 0 | 0 | 16553000 | 5342800 | GPX4 | 1383 | 4580369.41 | 16553000 | TRUE | FALSE | 4580369.41 | 16553000 | 0.276709 |
| TMED5 | 4 | 2 | 8.3 | 3 | 0 | 8.9955 | 0 | 0 | 0 | 14148000 | 4430000 | TMED5 | 1512 | 3929098.35 | 14148000 | TRUE | FALSE | 3929098.35 | 14148000 | 0.277714 |
| FAH | 3 | 2 | 6.9 | 3 | 0.000435 | 3.4226 | 3636800 | 0 | 0 | 12733000 | 844230 | FAH | 2377 | 3540570.656 | 12733000 | TRUE | FALSE | 3540570.656 | 12733000 | 0.278063 |
| STK26;STK | 14 | 2 | 8.9 | 5 | 0 | 5.0804 | 0 | 0 | 0 | 12919000 | 688370 | STK26 | 2471 | 3617638.011 | 12919000 | TRUE | FALSE | 3617638.011 | 12919000 | 0.280025 |
| PPP1R12A | 11 | 3 | 4.7 | 7 | 0 | 10.61 | 5810200 | 7801900 | 0 | 10362000 | 525790 | PPP1R12A | 2561 | 2909808.847 | 10362000 | TRUE | FALSE | 2909808.847 | 10362000 | 0.280815 |
| DNTTIP2 | 3 | 3 | 6.2 | 2 | 0.000797 | 2.6898 | 10425000 | 0 | 0 | 14273000 | 1933500 | DNTTIP2 | 1980 | 4022246.143 | 14273000 | TRUE | FALSE | 4022246.143 | 14273000 | 0.281808 |
| KIAA0368; | 3 | 3 | 1.7 | 5 | 0 | 4.8249 | 11457000 | 15211000 | 0 | 17291000 | 463440 | KIAA0368 | 2608 | 4934877.435 | 17291000 | TRUE | FALSE | 4934877.435 | 17291000 | 0.285402 |
| TACO1 | 1 | 2 | 10.1 | 2 | 0.000816 | 2.87 | 0 | 9668000 | 0 | 13297000 | 2461200 | TACO1 | 1838 | 3818867.198 | 13297000 | TRUE | FALSE | 3818867.198 | 13297000 | 0.287198 |
| MPDU1 | 14 | 3 | 33.7 | 0 | 0 | 4.5591 | 0 | 0 | 0 | 29204000 | 27344000 | MPDU1 | 579 | 8548433.586 | 29204000 | TRUE | FALSE | 8548433.586 | 29204000 | 0.292714 |
| SDHD | 8 | 2 | 14.2 | 5 | 0.000805 | 2.7695 | 17070000 | 17984000 | 0 | 8005400 | 10858000 | SDHD | 1001 | 2396234.793 | 8005400 | TRUE | FALSE | 2396234.793 | 8005400 | 0.299327 |
| MRPS9 | 1 | 2 | 5.8 | 1 | 0 | 4.3229 | 0 | 0 | 0 | 12369000 | 1565800 | MRPS9 | 2096 | 3755741.903 | 12369000 | TRUE | FALSE | 3755741.903 | 12369000 | 0.303642 |
| RPRD1B | 3 | 4 | 13.2 | 6 | 0 | 8.2806 | 0 | 0 | 0 | 15602000 | 5044900 | RPRD1B | 1415 | 4767089.449 | 15602000 | TRUE | FALSE | 4767089.449 | 15602000 | 0.305543 |
| COPS6 | 2 | 4 | 14.4 | 5 | 0 | 10.825 | 20322000 | 15397000 | 0 | 14923000 | 4594600 | COPS6 | 1488 | 4595290.367 | 14923000 | TRUE | FALSE | 4595290.367 | 14923000 | 0.307933 |
| ADCK3 | 4 | 2 | 14.1 | 1 | 0.00493 | 1.7313 | 0 | 0 | 0 | 7192500 | 1727700 | ADCK3 | 2041 | 2221058.338 | 7192500 | TRUE | FALSE | 2221058.338 | 7192500 | 0.308802 |
| RQCD1 | 3 | 2 | 7.7 | 5 | 0.000451 | 3.7653 | 6761300 | 5945400 | 0 | 10121000 | 1662200 | RQCD1 | 2060 | 3132196.824 | 10121000 | TRUE | FALSE | 3132196.824 | 10121000 | 0.309475 |
| ATP2B1 | 29 | 4 | 11.1 | 19 | 0 | 32.949 | 92226000 | 42205000 | 7559800 | 24037000 | 3184300 | ATP2B1 | 1716 | 7559800 | 24037000 | FALSE | FALSE | 7559800 | 24037000 | 0.314507 |
| MRPS34 | 2 | 2 | 6.7 | 4 | 0.001154 | 2.3927 | 0 | 13953000 | 0 | 6060100 | 2660600 | MRPS34 | 1801 | 1910002.549 | 6060100 | TRUE | FALSE | 1910002.549 | 6060100 | 0.315177 |
| MAP2K3 | 6 | 3 | 13.2 | 5 | 0 | 18.154 | 12066000 | 19616000 | 0 | 9083700 | 1900600 | MAP2K3 | 1989 | 2877469.01 | 9083700 | TRUE | FALSE | 2877469.01 | 9083700 | 0.316773 |
| NTMT1 | 4 | 3 | 24.8 | 12 | 0 | 14.562 | 17437000 | 20063000 | 0 | 11771000 | 9427700 | NTMT1 | 1078 | 3743892.013 | 11771000 | TRUE | FALSE | 3743892.013 | 11771000 | 0.318061 |
| MORF4L1;M | 9 | 2 | 6.6 | 2 | 0.001159 | 2.4201 | 0 | 0 | 0 | 5761300 | 592670 | MORF4L1 | 2525 | 1852091.51 | 5761300 | TRUE | FALSE | 1852091.51 | 5761300 | 0.321471 |
| USP10 | 4 | 2 | 20 | 5 | 0 | 5.3774 | 0 | 0 | 0 | 7678200 | 1749800 | USP10 | 2035 | 2514078.723 | 7678200 | TRUE | FALSE | 2514078.723 | 7678200 | 0.327431 |
| HYPK | 3 | 2 | 21.7 | 2 | 0 | 8.2655 | 14016000 | 8917300 | 0 | 8027800 | 5524100 | HYPK | 1364 | 2657337.769 | 8027800 | TRUE | FALSE | 2657337.769 | 8027800 | 0.331017 |
| WDR43 | 2 | 2 | 2.8 | 2 | 0.000787 | 2.5637 | 0 | 8199200 | 0 | 12609000 | 934030 | WDR43 | 2335 | 4505354.249 | 12609000 | TRUE | FALSE | 4505354.249 | 12609000 | 0.357313 |
| CPSF1 | 1 | 3 | 3 | 6 | 0 | 9.1271 | 7450600 | 6016100 | 0 | 9085900 | 272940 | CPSF1 | 2739 | 3319806.482 | 9085900 | TRUE | FALSE | 3319806.482 | 9085900 | 0.36538 |
| VAMP8 | 2 | 2 | 18.9 | 2 | 0.000457 | 3.9 | 0 | 0 | 0 | 12470000 | 4576100 | VAMP8 | 1492 | 4563073.051 | 12470000 | TRUE | FALSE | 4563073.051 | 12470000 | 0.365924 |
| ATP6AP2 | 20 | 3 | 15.7 | 5 | 0 | 15.011 | 17395000 | 9103800 | 0 | 7122400 | 3373000 | ATP6AP2 | 1678.5 | 2625323.153 | 7122400 | TRUE | FALSE | 2625323.153 | 7122400 | 0.368601 |
| NKRF | 2 | 2 | 2.8 | 2 | 0.004323 | 1.8797 | 0 | 0 | 0 | 7683200 | 366190 | NKRF | 2673 | 2841847.606 | 7683200 | TRUE | FALSE | 2841847.606 | 7683200 | 0.369878 |
| RAVER1 | 3 | 2 | 4.1 | 3 | 0 | 4.5762 | 0 | 0 | 0 | 9007600 | 673780 | RAVER1 | 2478 | 3380308.629 | 9007600 | TRUE | FALSE | 3380308.629 | 9007600 | 0.375273 |
| UAP1 | 4 | 17 | 41.5 | 62 | 0 | 136.29 | 336980000 | 610040000 | 5278000 | 14034000 | 31116000 | UAP1 | 522 | 5278000 | 14034000 | FALSE | FALSE | 5278000 | 14034000 | 0.376087 |
| GNB1L | 3 | 3 | 30.1 | 3 | 0.000816 | 2.8706 | 0 | 0 | 0 | 11406000 | 3169600 | GNB1L | 1720 | 4298463.331 | 11406000 | TRUE | FALSE | 4298463.331 | 11406000 | 0.37686 |
| ZFPL1 | 8 | 2 | 21.1 | 3 | 0.004324 | 1.8815 | 0 | 0 | 0 | 9907000 | 3212400 | ZFPL1 | 1713 | 3752034.693 | 9907000 | TRUE | FALSE | 3752034.693 | 9907000 | 0.378726 |
| TWF1 | 5 | 5 | 20 | 10 | 0 | 37.438 | 7572200 | 5938700 | 10934000 | 28791000 | 2036300 | TWF1 | 1954 | 10934000 | 28791000 | FALSE | FALSE | 10934000 | 28791000 | 0.379771 |
| MRPL41 | 1 | 2 | 14.6 | 3 | 0 | 5.5413 | 0 | 0 | 0 | 14027000 | 5131100 | MRPL41 | 1406 | 5327868.187 | 14027000 | TRUE | FALSE | 5327868.187 | 14027000 | 0.379829 |
| PTDSS1 | 2 | 2 | 4.7 | 4 | 0.000434 | 3.3727 | 7836600 | 0 | 0 | 0 | 554840 | PTDSS1 | 2546 | 2249225.235 | 5890483.835 | TRUE | TRUE | 2249225.235 | 5890483.835 | 0.38184 |
| TP53 | 19 | 3 | 7.9 | 6 | 0 | 4.3755 | 8055400 | 8135000 | 11604000 | 30332000 | 3144200 | TP53 | 1723 | 11604000 | 30332000 | FALSE | FALSE | 11604000 | 30332000 | 0.382566 |
| APP | 15 | 7 | 13.3 | 14 | 0 | 19.504 | 48519000 | 13887000 | 0 | 13106000 | 2232000 | APP | 1910 | 5044034.175 | 13106000 | TRUE | FALSE | 5044034.175 | 13106000 | 0.384865 |
| C19orf52 | 1 | 2 | 6.5 | 1 | 0.004307 | 1.8656 | 0 | 0 | 0 | 13290000 | 2603500 | C19orf52 | 1812 | 5224295.546 | 13290000 | TRUE | FALSE | 5224295.546 | 13290000 | 0.3931 |
| TTC1 | 2 | 4 | 15.1 | 3 | 0.000466 | 4.1809 | 16062000 | 23517000 | 0 | 12116000 | 3330500 | TTC1 | 1687.5 | 4767225.427 | 12116000 | TRUE | FALSE | 4767225.427 | 12116000 | 0.393465 |
| ENAH | 6 | 4 | 5.5 | 8 | 0 | 11.508 | 33199000 | 30077000 | 11308000 | 28732000 | 4004100 | ENAH | 1564 | 11308000 | 28732000 | FALSE | FALSE | 11308000 | 28732000 | 0.393568 |
| ZC3H14 | 13 | 2 | 14.2 | 4 | 0.000433 | 3.3581 | 5428000 | 7089300 | 0 | 6194600 | 2248400 | ZC3H14 | 1906 | 2445609.809 | 6194600 | TRUE | FALSE | 2445609.809 | 6194600 | 0.394797 |
| NUP107 | 4 | 3 | 3 | 6 | 0 | 4.9408 | 7247700 | 6930900 | 0 | 8304200 | 433520 | NUP107 | 2628 | 3326096.62 | 8304200 | TRUE | FALSE | 3326096.62 | 8304200 | 0.400532 |
| SLC35A4 | 1 | 5 | 49.5 | 9 | 0 | 10.443 | 37764000 | 23301000 | 0 | 0 | 13216000 | SLC35A4 | 909 | 1615477.249 | 4022876.896 | TRUE | TRUE | 1615477.249 | 4022876.896 | 0.401573 |
| RRAS | 1 | 2 | 14.7 | 1 | 0.00047 | 4.243 | 0 | 0 | 0 | 9837600 | 2553900 | RRAS | 1818 | 4008161.278 | 9837600 | TRUE | FALSE | 4008161.278 | 9837600 | 0.407433 |
| AHCYL2;AH | 7 | 5 | 10 | 8 | 0 | 10.907 | 16777000 | 18386000 | 0 | 11954000 | 1872500 | AHCYL2 | 1996 | 4901962.119 | 11954000 | TRUE | FALSE | 4901962.119 | 11954000 | 0.410069 |
| VPS45 | 8 | 2 | 5.4 | 5 | 0 | 4.321 | 0 | 8069000 | 0 | 6326200 | 748110 | VPS45 | 2436 | 2598197.498 | 6326200 | TRUE | FALSE | 2598197.498 | 6326200 | 0.410704 |
| MRPS10 | 1 | 2 | 13.4 | 3 | 0.000449 | 3.7265 | 6051500 | 6554700 | 0 | 7475500 | 1442700 | MRPS10 | 2137 | 3088401.018 | 7475500 | TRUE | FALSE | 3088401.018 | 7475500 | 0.413136 |
| ABCC3 | 4 | 2 | 1.9 | 2 | 0 | 4.7562 | 0 | 0 | 0 | 7211300 | 193230 | ABCC3 | 2800 | 3001561.088 | 7211300 | TRUE | FALSE | 3001561.088 | 7211300 | 0.41623 |
| HMGCS1 | 2 | 7 | 17.5 | 17 | 0 | 16.718 | 17929000 | 14329000 | 34519000 | 82373000 | 3965000 | HMGCS1 | 1569 | 34519000 | 82373000 | FALSE | FALSE | 34519000 | 82373000 | 0.419057 |
| MIA3 | 5 | 3 | 2.1 | 3 | 0 | 4.892 | 3495900 | 0 | 0 | 8449500 | 84475 | MIA3 | 2895 | 3546685.038 | 8449500 | TRUE | FALSE | 3546685.038 | 8449500 | 0.419751 |
| NUTF2 | 2 | 2 | 23.6 | 3 | 0 | 15.595 | 29666000 | 32948000 | 0 | 17802000 | 14575000 | NUTF2 | 853 | 7485885.827 | 17802000 | TRUE | FALSE | 7485885.827 | 17802000 | 0.420508 |
| ROCK2 | 4 | 10 | 10.4 | 12 | 0 | 17.76 | 17772000 | 7871500 | 22487000 | 53109000 | 1087600 | ROCK2 | 2273 | 22487000 | 53109000 | FALSE | FALSE | 22487000 | 53109000 | 0.423412 |
| SMARCA4;S | 28 | 2 | 1.8 | 3 | 0.000807 | 2.7971 | 6330300 | 8885800 | 0 | 9875400 | 526790 | SMARCA4 | 2559 | 4225986.012 | 9875400 | TRUE | FALSE | 4225986.012 | 9875400 | 0.427931 |
| ATP6V1C1 | 5 | 6 | 14.9 | 10 | 0 | 7.8797 | 32490000 | 28802000 | 0 | 9055500 | 2635400 | ATP6V1C1 | 1804 | 3882390.133 | 9055500 | TRUE | FALSE | 3882390.133 | 9055500 | 0.428733 |
| CLPTM1 | 5 | 3 | 8.5 | 2 | 0 | 5.5422 | 0 | 0 | 0 | 7826000 | 535550 | CLPTM1 | 2553 | 3383909.968 | 7826000 | TRUE | FALSE | 3383909.968 | 7826000 | 0.432393 |
| NUP160 | 6 | 2 | 1.9 | 10 | 0.000425 | 3.2203 | 5721700 | 6118200 | 0 | 7799600 | 504560 | NUP160 | 2578 | 3376078.602 | 7799600 | TRUE | FALSE | 3376078.602 | 7799600 | 0.432853 |
| PPP6R3 | 12 | 4 | 6.6 | 6 | 0 | 7.4166 | 0 | 8253300 | 0 | 7788500 | 787010 | PPP6R3 | 2410 | 3386202.483 | 7788500 | TRUE | FALSE | 3386202.483 | 7788500 | 0.43477 |
| MRPL46 | 1 | 2 | 9.3 | 3 | 0 | 6.6752 | 0 | 0 | 0 | 7278000 | 1242800 | MRPL46 | 2206 | 3173285.201 | 7278000 | TRUE | FALSE | 3173285.201 | 7278000 | 0.436011 |
| CGN | 1 | 2 | 1.8 | 2 | 0.000796 | 2.6622 | 0 | 0 | 0 | 4470700 | 58097 | CGN | 2910 | 1951940.607 | 4470700 | TRUE | FALSE | 1951940.607 | 4470700 | 0.436607 |
| CAV1 | 2 | 3 | 51.7 | 33 | 0 | 56.426 | 645730000 | 294100000 | 0 | 0 | 94491000 | CAV1 | 241 | 2392927.397 | 5405398.678 | TRUE | TRUE | 2392927.397 | 5405398.678 | 0.442692 |

| | | | | | | | | | | | | | | | | | | | | |
|---|---|---|---|---|---|---|---|---|---|---|---|---|---|---|---|---|---|---|---|---|
| CALD1 | 12 | 16 | 32.7 | 29 | 0 | 66.315 | 150100000 | 168860000 | 4524800 | 10200000 | 13795000 | CALD1 | 884 | 4524800 | 10200000 | FALSE | FALSE | 4524800 | 10200000 | 0.443608 |
| CMBL | 1 | 5 | 19.2 | 5 | 0 | 9.9542 | 0 | 9702500 | 25409000 | 57262000 | 4460600 | CMBL | 1508 | 25409000 | 57262000 | FALSE | FALSE | 25409000 | 57262000 | 0.443732 |
| PDLIM7 | 2 | 2 | 5.7 | 2 | 0.000825 | 2.9513 | 0 | 0 | 0 | 6143500 | 878440 | PDLIM7 | 2361 | 2728972.294 | 6143500 | TRUE | FALSE | 2728972.294 | 6143500 | 0.444205 |
| APOO | 4 | 3 | 20.8 | 4 | 0 | 4.8205 | 10856000 | 0 | 0 | 3849600 | 2225500 | APOO | 1912 | 1714632.831 | 3849600 | TRUE | FALSE | 1714632.831 | 3849600 | 0.445405 |
| HDHD3 | 1 | 2 | 10.8 | 0 | 0.00222 | 2.0892 | 0 | 0 | 0 | 3396400 | 395960 | HDHD3 | 2647 | 1516333.085 | 3396400 | TRUE | FALSE | 1516333.085 | 3396400 | 0.446453 |
| FAM98A | 2 | 2 | 9.3 | 3 | 0 | 5.3526 | 0 | 0 | 0 | 3907900 | 784280 | FAM98A | 2413 | 1747358.046 | 3907900 | TRUE | FALSE | 1747358.046 | 3907900 | 0.447135 |
| LSM1 | 3 | 3 | 30.1 | 6 | 0 | 5.2371 | 11661000 | 15951000 | 0 | 7682600 | 6393000 | LSM1 | 1298 | 3468484.21 | 7682600 | TRUE | FALSE | 3468484.21 | 7682600 | 0.451473 |
| CIAO1 | 1 | 2 | 7.4 | 3 | 0 | 5.589 | 6043500 | 5533600 | 6119400 | 13420000 | 1588900 | CIAO1 | 2086 | 6119400 | 13420000 | FALSE | FALSE | 6119400 | 13420000 | 0.455991 |
| RSU1 | 2 | 3 | 10.3 | 4 | 0.000812 | 2.8293 | 11909000 | 0 | 0 | 0 | 1604800 | RSU1 | 2078 | 2378016.659 | 5171429.994 | TRUE | TRUE | 2378016.659 | 5171429.994 | 0.459837 |
| MRPS35 | 3 | 2 | 8 | 6 | 0 | 6.8733 | 9026900 | 12039000 | 0 | 10398000 | 2247300 | MRPS35 | 1907 | 4786287.751 | 10398000 | TRUE | FALSE | 4786287.751 | 10398000 | 0.460308 |
| PXMP4 | 3 | 2 | 9.9 | 2 | 0.000804 | 2.7442 | 4775600 | 0 | 0 | 0 | 670610 | PXMP4 | 2482 | 1499695.913 | 3222805.671 | TRUE | TRUE | 1499695.913 | 3222805.671 | 0.465339 |
| CCAR1 | 4 | 4 | 3.9 | 5 | 0 | 6.939 | 14749000 | 12913000 | 0 | 11039000 | 984560 | CCAR1 | 2316 | 5203119.365 | 11039000 | TRUE | FALSE | 5203119.365 | 11039000 | 0.47134 |
| PRPF31 | 8 | 2 | 5.3 | 2 | 0 | 5.6257 | 0 | 0 | 0 | 3245600 | 423320 | PRPF31 | 2632 | 1560715.874 | 3245600 | TRUE | FALSE | 1560715.874 | 3245600 | 0.480871 |
| GCSH | 3 | 2 | 30 | 5 | 0 | 13.451 | 12973000 | 16574000 | 0 | 17146000 | 17867000 | GCSH | 755 | 8265592.738 | 17146000 | TRUE | FALSE | 8265592.738 | 17146000 | 0.482071 |
| PURB | 1 | 2 | 7.1 | 2 | 0.000455 | 3.8658 | 0 | 0 | 0 | 15697000 | 2446100 | PURB | 1841.5 | 7690658.683 | 15697000 | TRUE | FALSE | 7690658.683 | 15697000 | 0.489944 |
| ITM2B | 5 | 5 | 25.9 | 5 | 0 | 19.574 | 56414000 | 0 | 0 | 0 | 4972900 | ITM2B | 1425 | 832092.1134 | 1688291.144 | TRUE | TRUE | 832092.1134 | 1688291.144 | 0.492861 |
| RRAS2 | 5 | 2 | 14.5 | 3 | 0 | 5.3032 | 7839100 | 9452200 | 0 | 7823600 | 2683300 | RRAS2 | 1798 | 3887360.688 | 7823600 | TRUE | FALSE | 3887360.688 | 7823600 | 0.496876 |
| GADD45GIF | 1 | 2 | 10.8 | 2 | 0 | 4.3014 | 0 | 0 | 0 | 9497400 | 2452100 | GADD45GIF | 1839 | 4795232.652 | 9497400 | TRUE | FALSE | 4795232.652 | 9497400 | 0.5049 |
| DCTN4 | 6 | 2 | 6 | 0 | 0.00045 | 3.7547 | 0 | 0 | 0 | 3828400 | 908250 | DCTN4 | 2348 | 1944060.444 | 3828400 | TRUE | FALSE | 1944060.444 | 3828400 | 0.5078 |
| TRAPPC3 | 5 | 4 | 22.3 | 4 | 0 | 7.2578 | 0 | 19316000 | 9701100 | 18845000 | 4536500 | TRAPPC3 | 1501.5 | 9701100 | 18845000 | FALSE | FALSE | 9701100 | 18845000 | 0.514784 |
| UGT1A10;U | 20 | 2 | 5.2 | 2 | 0 | 4.5747 | 0 | 0 | 0 | 6740200 | 308310 | UGT1A10 | 2715 | 3492579.208 | 6740200 | TRUE | FALSE | 3492579.208 | 6740200 | 0.518171 |
| GHITM | 1 | 3 | 9.6 | 5 | 0 | 5.3608 | 3873400 | 2945400 | 11173000 | 21378000 | 2199000 | GHITM | 1916 | 11173000 | 21378000 | FALSE | FALSE | 11173000 | 21378000 | 0.52264 |
| RRP9 | 1 | 5 | 9.7 | 7 | 0 | 8.9049 | 36967000 | 40263000 | 14109000 | 26898000 | 4562700 | RRP9 | 1496 | 14109000 | 26898000 | FALSE | FALSE | 14109000 | 26898000 | 0.524537 |
| ME1 | 2 | 2 | 5.4 | 5 | 0 | 5.2203 | 5305700 | 4221400 | 0 | 10861000 | 890750 | ME1 | 2356 | 5772091.038 | 10861000 | TRUE | FALSE | 5772091.038 | 10861000 | 0.531451 |
| GEMIN4 | 2 | 2 | 2.1 | 4 | 0.000425 | 3.2335 | 7760800 | 7776000 | 0 | 7490100 | 377070 | GEMIN4 | 2664 | 3984697.284 | 7490100 | TRUE | FALSE | 3984697.284 | 7490100 | 0.531995 |
| MGEA5 | 5 | 3 | 3.5 | 4 | 0 | 4.4284 | 0 | 0 | 0 | 12659000 | 785060 | MGEA5 | 2412 | 6741996.842 | 12659000 | TRUE | FALSE | 6741996.842 | 12659000 | 0.532585 |
| EXOSC2 | 6 | 2 | 9 | 3 | 0.004 | 1.9237 | 0 | 0 | 0 | 3777500 | 1218000 | EXOSC2 | 2217 | 2045092.353 | 3777500 | TRUE | FALSE | 2045092.353 | 3777500 | 0.541388 |
| CYP51A1 | 4 | 3 | 7.7 | 6 | 0.000452 | 3.7824 | 4336200 | 4480600 | 5364600 | 9840200 | 829930 | CYP51A1 | 2384 | 5364600 | 9840200 | FALSE | FALSE | 5364600 | 9840200 | 0.545172 |
| NDUFS4 | 2 | 2 | 13.7 | 6 | 0.000463 | 4.0426 | 12094000 | 10287000 | 0 | 10577000 | 3949200 | NDUFS4 | 1576 | 5786973.211 | 10577000 | TRUE | FALSE | 5786973.211 | 10577000 | 0.547128 |
| TCEB1 | 4 | 4 | 62.9 | 18 | 0 | 24.836 | 63751000 | 60923000 | 39623000 | 72309000 | 28979000 | TCEB1 | 554 | 39623000 | 72309000 | FALSE | FALSE | 39623000 | 72309000 | 0.547968 |
| ATP6V1G1 | 1 | 4 | 28.8 | 13 | 0 | 21.854 | 57749000 | 42610000 | 17679000 | 32259000 | 20216000 | ATP6V1G1 | 710 | 17679000 | 32259000 | FALSE | FALSE | 17679000 | 32259000 | 0.548033 |
| WDR82 | 1 | 2 | 10.9 | 6 | 0 | 9.367 | 6266400 | 6416000 | 0 | 8136900 | 1036200 | WDR82 | 2294 | 4516418.823 | 8136900 | TRUE | FALSE | 4516418.823 | 8136900 | 0.555054 |
| THOC6 | 3 | 5 | 18.5 | 10 | 0 | 12.049 | 8352600 | 10191000 | 8603500 | 15462000 | 1944500 | THOC6 | 1977 | 8603500 | 15462000 | FALSE | FALSE | 8603500 | 15462000 | 0.556429 |
| ITGA2 | 4 | 3 | 4.2 | 1 | 0.000829 | 2.9941 | 0 | 0 | 0 | 16161000 | 908190 | ITGA2 | 2349 | 9056474.214 | 16161000 | TRUE | FALSE | 9056474.214 | 16161000 | 0.560391 |
| PIGS | 3 | 2 | 4.5 | 3 | 0 | 4.4067 | 8695400 | 8840000 | 0 | 7377800 | 1164800 | PIGS | 2241 | 4143711.509 | 7377800 | TRUE | FALSE | 4143711.509 | 7377800 | 0.561646 |
| SLC7A5 | 6 | 5 | 10.8 | 24 | 0 | 13.392 | 42841000 | 40043000 | 34845000 | 61990000 | 8340100 | SLC7A5 | 1149 | 34845000 | 61990000 | FALSE | FALSE | 34845000 | 61990000 | 0.562107 |
| EIF4B | 7 | 8 | 16.1 | 37 | 0 | 80.864 | 92345000 | 89166000 | 118050000 | 209180000 | 15666000 | EIF4B | 822 | 118050000 | 209180000 | FALSE | FALSE | 118050000 | 209180000 | 0.564346 |
| COMT | 6 | 10 | 60.2 | 34 | 0 | 172.78 | 16532000 | 11301000 | 268160000 | 468590000 | 44026000 | COMT | 418 | 268160000 | 468590000 | FALSE | FALSE | 268160000 | 468590000 | 0.57227 |
| GGCT | 7 | 5 | 31.4 | 10 | 0 | 18.733 | 0 | 34524000 | 69699000 | 119570000 | 12946000 | GGCT | 924 | 69699000 | 119570000 | FALSE | FALSE | 69699000 | 119570000 | 0.582914 |
| PGP | 2 | 3 | 11.8 | 6 | 0 | 6.3029 | 10902000 | 10340000 | 9688100 | 16618000 | 2197600 | PGP | 1917 | 9688100 | 16618000 | FALSE | FALSE | 9688100 | 16618000 | 0.582988 |
| SPTLC1 | 5 | 4 | 9.3 | 8 | 0 | 12.315 | 17692000 | 13207000 | 10566000 | 18106000 | 2546600 | SPTLC1 | 1822 | 10566000 | 18106000 | FALSE | FALSE | 10566000 | 18106000 | 0.583563 |
| SLC16A3 | 11 | 6 | 14 | 19 | 0 | 13.115 | 128960000 | 73748000 | 7887300 | 13467000 | 12216000 | SLC16A3 | 947 | 7887300 | 13467000 | FALSE | FALSE | 7887300 | 13467000 | 0.585676 |
| FTH1 | 6 | 2 | 15 | 4 | 0.000799 | 2.7064 | 23914000 | 12454000 | 0 | 0 | 5419500 | FTH1 | 1374 | 2197030.903 | 3731958.578 | TRUE | TRUE | 2197030.903 | 3731958.578 | 0.588707 |
| AP1M1 | 12 | 2 | 10.3 | 5 | 0 | 6.1252 | 9991100 | 8921300 | 0 | 8483500 | 1205700 | AP1M1 | 2225 | 5008857.607 | 8483500 | TRUE | FALSE | 5008857.607 | 8483500 | 0.590423 |
| DNAJC10 | 5 | 4 | 5.6 | 7 | 0 | 8.4097 | 9230900 | 0 | 11099000 | 18790000 | 1056300 | DNAJC10 | 2287 | 11099000 | 18790000 | FALSE | FALSE | 11099000 | 18790000 | 0.590687 |
| MT2A;MT1X | 8 | 2 | 67.2 | 14 | 0 | 25.474 | 353990000 | 185480000 | 6217900 | 10469000 | 184350000 | MT2A | 130 | 6217900 | 10469000 | FALSE | FALSE | 6217900 | 10469000 | 0.593934 |
| CCDC6 | 1 | 2 | 4.9 | 3 | 0.000819 | 2.9051 | 0 | 15635000 | 24860000 | 41625000 | 2954100 | CCDC6 | 1759 | 24860000 | 41625000 | FALSE | FALSE | 24860000 | 41625000 | 0.597237 |
| CASP3 | 3 | 2 | 18.8 | 2 | 0.002219 | 2.0838 | 0 | 0 | 0 | 13847000 | 6067200 | CASP3 | 1321 | 8273245.51 | 13847000 | TRUE | FALSE | 8273245.51 | 13847000 | 0.597476 |
| KDELR1;KD | 5 | 2 | 8.6 | 6 | 0.000781 | 2.4999 | 70766000 | 55455000 | 81466000 | 135910000 | 27238000 | KDELR1 | 580 | 81466000 | 135910000 | FALSE | FALSE | 81466000 | 135910000 | 0.599411 |
| AP2M1 | 10 | 9 | 23.3 | 16 | 0 | 14.87 | 63222000 | 59069000 | 28691000 | 47797000 | 6899400 | AP2M1 | 1256 | 28691000 | 47797000 | FALSE | FALSE | 28691000 | 47797000 | 0.600268 |
| CRABP2 | 4 | 8 | 45.7 | 28 | 0 | 25.786 | 0 | 0 | 202460000 | 335550000 | 56813000 | CRABP2 | 354 | 202460000 | 335550000 | FALSE | FALSE | 202460000 | 335550000 | 0.603368 |
| EIF4A3 | 2 | 9 | 25.3 | 16 | 0 | 19.042 | 57678000 | 51803000 | 55247000 | 91323000 | 9353800 | EIF4A3 | 1083 | 55247000 | 91323000 | FALSE | FALSE | 55247000 | 91323000 | 0.604963 |
| LARP4 | 13 | 2 | 6.9 | 3 | 0.000418 | 3.0799 | 8250300 | 6109000 | 0 | 5580500 | 1617900 | LARP4 | 2073 | 3377730.374 | 5580500 | TRUE | FALSE | 3377730.374 | 5580500 | 0.605274 |
| CRNKL1 | 4 | 2 | 2.9 | 2 | 0 | 7.1441 | 0 | 0 | 0 | 4385000 | 283360 | CRNKL1 | 2736 | 2667721.536 | 4385000 | TRUE | FALSE | 2667721.536 | 4385000 | 0.608374 |
| SART3 | 10 | 3 | 4.9 | 1 | 0 | 9.381 | 0 | 0 | 0 | 5020900 | 300810 | SART3 | 2723 | 3060908.901 | 5020900 | TRUE | FALSE | 3060908.901 | 5020900 | 0.609634 |
| FAM120A | 8 | 4 | 5.7 | 7 | 0 | 7.8066 | 8220700 | 7809900 | 0 | 10898000 | 433630 | FAM120A | 2627 | 6660589.223 | 10898000 | TRUE | FALSE | 6660589.223 | 10898000 | 0.611175 |
| SCRIB | 7 | 2 | 2.3 | 1 | 0 | 6.2307 | 0 | 0 | 0 | 5358000 | 313410 | SCRIB | 2707 | 3317619.371 | 5358000 | TRUE | FALSE | 3317619.371 | 5358000 | 0.61919 |
| ZFR | 2 | 3 | 3.5 | 8 | 0 | 5.3886 | 6252300 | 6845200 | 5676700 | 9151400 | 503230 | ZFR | 2581 | 5676700 | 9151400 | FALSE | FALSE | 5676700 | 9151400 | 0.620309 |
| RPS28 | 1 | 3 | 46.4 | 18 | 0 | 32.173 | 240830000 | 213260000 | 186660000 | 300140000 | 272350000 | RPS28 | 81 | 186660000 | 300140000 | FALSE | FALSE | 186660000 | 300140000 | 0.62191 |
| S100A16 | 1 | 4 | 44.7 | 11 | 0 | 22.933 | 0 | 0 | 94429000 | 151830000 | 28852000 | S100A16 | 558 | 94429000 | 151830000 | FALSE | FALSE | 94429000 | 151830000 | 0.621939 |
| TXNDC9 | 6 | 2 | 10.6 | 3 | 0.000422 | 3.1506 | 12185000 | 0 | 0 | 0 | 2876600 | TXNDC9 | 1767 | 1691209.829 | 2711326.82 | TRUE | TRUE | 1691209.829 | 2711326.82 | 0.623757 |
| NDUFA10 | 9 | 4 | 12.7 | 8 | 0 | 7.3207 | 10885000 | 13395000 | 12464000 | 19903000 | 2535300 | NDUFA10 | 1825 | 12464000 | 19903000 | FALSE | FALSE | 12464000 | 19903000 | 0.626237 |
| DDX42 | 3 | 5 | 8.8 | 9 | 0 | 8.0222 | 17565000 | 15455000 | 20153000 | 32147000 | 1784300 | DDX42 | 2021 | 20153000 | 32147000 | FALSE | FALSE | 20153000 | 32147000 | 0.626901 |
| ERAP1 | 2 | 2 | 2.3 | 2 | 0.000457 | 3.9006 | 9172900 | 0 | 0 | 0 | 321750 | ERAP1 | 2699 | 1921469.337 | 3052627.784 | TRUE | TRUE | 1921469.337 | 3052627.784 | 0.629448 |
| ADSL | 15 | 6 | 13.5 | 10 | 0 | 13.466 | 52298000 | 60167000 | 23805000 | 37741000 | 6284200 | ADSL | 1306 | 23805000 | 37741000 | FALSE | FALSE | 23805000 | 37741000 | 0.630746 |
| TXLNG | 2 | 2 | 4.8 | 3 | 0.003988 | 1.9126 | 5131500 | 4412600 | 0 | 6142000 | 853280 | TXLNG | 2375 | 3899871.938 | 6142000 | TRUE | FALSE | 3899871.938 | 6142000 | 0.634951 |
| RPS7 | 3 | 7 | 31.4 | 38 | 0 | 13.35 | 400330000 | 376530000 | 320700000 | 502950000 | 145220000 | RPS7 | 173 | 320700000 | 502950000 | FALSE | FALSE | 320700000 | 502950000 | 0.637638 |
| KRR1 | 2 | 2 | 6.5 | 0 | 0.009495 | 1.4319 | 0 | 0 | 0 | 4964300 | 995500 | KRR1 | 2314 | 3165580.566 | 4964300 | TRUE | FALSE | 3165580.566 | 4964300 | 0.637669 |
| PELO | 1 | 2 | 6.5 | 3 | 0.000461 | 4.0124 | 11656000 | 0 | 0 | 0 | 802490 | PELO | 2403 | 1815404.158 | 2846067.215 | TRUE | TRUE | 1815404.158 | 2846067.215 | 0.637864 |
| CTSB | 12 | 9 | 31.6 | 19 | 0 | 23.093 | 96389000 | 121970000 | 17811000 | 27855000 | 13227000 | CTSB | 908 | 17811000 | 27855000 | FALSE | FALSE | 17811000 | 27855000 | 0.639418 |
| SUCLA2 | 11 | 4 | 9.4 | 12 | 0 | 6.7526 | 22553000 | 24013000 | 25444000 | 39691000 | 3913500 | SUCLA2 | 1582 | 25444000 | 39691000 | FALSE | FALSE | 25444000 | 39691000 | 0.641052 |
| NAA10 | 8 | 5 | 27.3 | 15 | 0 | 19.361 | 30107000 | 30398000 | 13432000 | 20945000 | 5404000 | NAA10 | 1376 | 13432000 | 20945000 | FALSE | FALSE | 13432000 | 20945000 | 0.641299 |
| SECTM1 | 1 | 2 | 7.3 | 2 | 0.000468 | 4.2112 | 34040000 | 0 | 0 | 0 | 4875400 | SECTM1 | 1439 | 2013543.092 | 3136634.253 | TRUE | TRUE | 2013543.092 | 3136634.253 | 0.641944 |
| SLC9A3R2 | 6 | 2 | 5.5 | 2 | 0.000422 | 3.1517 | 0 | 0 | 0 | 6149600 | 561780 | SLC9A3R2 | 2542 | 3958053.016 | 6149600 | TRUE | FALSE | 3958053.016 | 6149600 | 0.643628 |
| TGOLN2 | 6 | 4 | 13.5 | 7 | 0 | 8.7531 | 7727700 | 5121600 | 6181900 | 9597000 | 1430000 | TGOLN2 | 2144.5 | 6181900 | 9597000 | FALSE | FALSE | 6181900 | 9597000 | 0.644149 |
| ACOT9 | 6 | 10 | 28 | 19 | 0 | 21.562 | 72144000 | 64137000 | 12468000 | 19353000 | 6861300 | ACOT9 | 1260 | 12468000 | 19353000 | FALSE | FALSE | 12468000 | 19353000 | 0.644241 |
| HNRNPH3 | 6 | 4 | 13.3 | 7 | 0 | 9.3836 | 53126000 | 49192000 | 40451000 | 62765000 | 11512000 | HNRNPH3 | 974 | 40451000 | 62765000 | FALSE | FALSE | 40451000 | 62765000 | 0.644483 |
| PSMA1 | 5 | 15 | 61.6 | 63 | 0 | 41.564 | 273140000 | 271700000 | 230320000 | 355950000 | 57974000 | PSMA1 | 347 | 230320000 | 355950000 | FALSE | FALSE | 230320000 | 355950000 | 0.647057 |
| HIST1H1E | 1 | 4 | 42.9 | 80 | 0 | 32.448 | 1.27E+09 | 1035200000 | 1844300000 | 2839900000 | 666090000 | HIST1H1E | 32 | 1844300000 | 2839900000 | FALSE | FALSE | 1844300000 | 2839900000 | 0.649424 |
| EIF1AX;EI | 4 | 5 | 33.3 | 25 | 0 | 12.417 | 105140000 | 84623000 | 125880000 | 193700000 | 61579000 | EIF1AX | 336 | 125880000 | 193700000 | FALSE | FALSE | 125880000 | 193700000 | 0.649871 |
| DNAJC19 | 4 | 2 | 28.4 | 1 | 0 | 9.1232 | 0 | 0 | 0 | 3203900 | 215790 | DNAJC19 | 2785 | 2082742.928 | 3203900 | TRUE | FALSE | 2082742.928 | 3203900 | 0.650065 |
| GRSF1 | 6 | 3 | 10.8 | 5 | 0.000468 | 4.2031 | 0 | 9824000 | 0 | 0 | 805420 | GRSF1 | 2399 | 2555404.971 | 3922931.601 | TRUE | TRUE | 2555404.971 | 3922931.601 | 0.651402 |
| HIST1H1B | 1 | 7 | 41.2 | 48 | 0 | 22.902 | 331210000 | 287590000 | 508700000 | 778150000 | 173030000 | HIST1H1B | 143 | 508700000 | 778150000 | FALSE | FALSE | 508700000 | 778150000 | 0.65373 |
| PLCB3 | 2 | 2 | 2 | 2 | 0.001157 | 2.4139 | 0 | 0 | 0 | 3721900 | 115850 | PLCB3 | 2875 | 2435993.285 | 3721900 | TRUE | FALSE | 2435993.285 | 3721900 | 0.654503 |
| PFKL | 3 | 6 | 13.6 | 5 | 0 | 13.887 | 0 | 16535000 | 26706000 | 40674000 | 2081500 | PFKL | 1943 | 26706000 | 40674000 | FALSE | FALSE | 26706000 | 40674000 | 0.656587 |
| RPL35A | 4 | 7 | 52.7 | 53 | 0 | 23.296 | 573680000 | 630930000 | 482040000 | 733780000 | 317250000 | RPL35A | 69 | 482040000 | 733780000 | FALSE | FALSE | 482040000 | 733780000 | 0.656927 |
| HNRNPUL1 | 14 | 6 | 10.7 | 19 | 0 | 29.974 | 39320000 | 43785000 | 83492000 | 55576000 | 6272400 | HNRNPUL1 | 1309 | 83492000 | 55576000 | FALSE | FALSE | 83492000 | 55576000 | 1.502303 |
| ACP1 | 7 | 4 | 63.8 | 12 | 0 | 23.853 | 9608300 | 0 | 38352000 | 25517000 | 10535000 | ACP1 | 1019 | 38352000 | 25517000 | FALSE | FALSE | 38352000 | 25517000 | 1.502998 |
| DPYSL2 | 11 | 26 | 53.8 | 145 | 0 | 323.31 | 1110800000 | 1639100000 | 279100000 | 185360000 | 80412000 | DPYSL2 | 272 | 279100000 | 185360000 | FALSE | FALSE | 279100000 | 185360000 | 1.505719 |
| MRPL49 | 4 | 2 | 16.3 | 8 | 0 | 24.006 | 7946700 | 10524000 | 16054000 | 10658000 | 3516600 | MRPL49 | 1650 | 16054000 | 10658000 | FALSE | FALSE | 16054000 | 10658000 | 1.506286 |
| CEBPZ | 1 | 4 | 4.6 | 6 | 0 | 6.366 | 6964700 | 0 | 14070000 | 9331200 | 702590 | CEBPZ | 2461 | 14070000 | 9331200 | FALSE | FALSE | 14070000 | 9331200 | 1.507845 |
| POLR2B | 4 | 4 | 5.7 | 5 | 0 | 6.5062 | 9381500 | 9819700 | 13973000 | 9245900 | 593440 | POLR2B | 2524 | 13973000 | 9245900 | FALSE | FALSE | 13973000 | 9245900 | 1.511264 |
| EIF3K | 6 | 3 | 25.5 | 8 | 0 | 7.6615 | 25954000 | 3.00E+07 | 54366000 | 35944000 | 17699000 | EIF3K | 759 | 54366000 | 35944000 | FALSE | FALSE | 54366000 | 35944000 | 1.512519 |
| TPP1 | 7 | 2 | 6.8 | 2 | 0.000827 | 2.9805 | 0 | 0 | 7185700 | 4738200 | 498530 | TPP1 | 2584 | 7185700 | 4738200 | FALSE | FALSE | 7185700 | 4738200 | 1.516546 |
| EHD4 | 1 | 5 | 15.9 | 11 | 0 | 14.501 | 0 | 0 | 47940000 | 31495000 | 2062200 | EHD4 | 1949 | 47940000 | 31495000 | FALSE | FALSE | 47940000 | 31495000 | 1.522146 |
| MGST3 | 3 | 2 | 16.3 | 8 | 0 | 11.473 | 0 | 27003000 | 0 | 0 | 12785000 | MGST3 | 930 | 6615237.443 | 4341152.895 | TRUE | TRUE | 6615237.443 | 4341152.895 | 1.523843 |
| PCK2 | 11 | 6 | 11.4 | 15 | 0 | 26.143 | 10889000 | 19826000 | 46790000 | 30692000 | 2486500 | PCK2 | 1832 | 46790000 | 30692000 | FALSE | FALSE | 46790000 | 30692000 | 1.524501 |
| SCRN1 | 6 | 5 | 15.9 | 11 | 0 | 10.113 | 29356000 | 21947000 | 23794000 | 15602000 | 4870900 | SCRN1 | 1440 | 23794000 | 15602000 | FALSE | FALSE | 23794000 | 15602000 | 1.525061 |
| OTUB1 | 7 | 10 | 45.2 | 45 | 0 | 66.789 | 146450000 | 155440000 | 167640000 | 109860000 | 35741000 | OTUB1 | 479 | 167640000 | 109860000 | FALSE | FALSE | 167640000 | 109860000 | 1.525942 |
| RAB3GAP2 | 1 | 3 | 2.7 | 5 | 0 | 5.1258 | 8442700 | 4719800 | 4753600 | 0 | 313700 | RAB3GAP2 | 2706 | 4753600 | 3107581.502 | FALSE | TRUE | 4753600 | 3107581.502 | 1.529678 |
| C12orf10 | 3 | 3 | 17.8 | 7 | 0 | 15.272 | 0 | 0 | 24088000 | 15747000 | 2824600 | C12orf10 | 1780 | 24088000 | 15747000 | FALSE | FALSE | 24088000 | 15747000 | 1.529688 |
| RRP12 | 5 | 11 | 11.9 | 20 | 0 | 25.894 | 33967000 | 28050000 | 72866000 | 47424000 | 2331100 | RRP12 | 1879 | 72866000 | 47424000 | FALSE | FALSE | 72866000 | 47424000 | 1.536479 |
| ATP13A3 | 3 | 2 | 1.5 | 1 | 0.009504 | 1.4473 | 7298900 | 0 | 0 | 0 | 184700 | ATP13A3 | 2808 | 3570066.065 | 2322833.376 | TRUE | TRUE | 3570066.065 | 2322833.376 | 1.536945 |
| RFC4 | 9 | 4 | 17.4 | 5 | 0 | 16.099 | 6263900 | 6442500 | 13933000 | 9006100 | 1589500 | RFC4 | 2084.5 | 13933000 | 9006100 | FALSE | FALSE | 13933000 | 9006100 | 1.547063 |
| FASTKD5 | 1 | 2 | 2.4 | 2 | 0.000423 | 3.1542 | 4895300 | 0 | 0 | 0 | 305530 | FASTKD5 | 2718 | 6228202.735 | 4023496.821 | TRUE | TRUE | 6228202.735 | 4023496.821 | 1.547958 |
| pk;ZAK | 5 | 2 | 5.9 | 3 | 0.000433 | 3.3551 | 0 | 0 | 4523800 | 0 | 1049200 | pk | 2290 | 4523800 | 2912833.047 | FALSE | TRUE | 4523800 | 2912833.047 | 1.553058 |
| DERL1 | 4 | 4 | 26.5 | 8 | 0 | 10.148 | 47524000 | 30945000 | 47860000 | 30739000 | 15168000 | DERL1 | 836 | 47860000 | 30739000 | FALSE | FALSE | 47860000 | 30739000 | 1.55698 |
| HSPB11 | 3 | 3 | 33.3 | 7 | 0 | 15.629 | 30645000 | 24236000 | 23593000 | 15081000 | 16934000 | HSPB11 | 777 | 23593000 | 15081000 | FALSE | FALSE | 23593000 | 15081000 | 1.564419 |
| HMOX1 | 2 | 3 | 13.9 | 3 | 0 | 4.6954 | 16387000 | 0 | 0 | 0 | 1867300 | HMOX1 | 1999 | 3406850.505 | 2175828.033 | TRUE | TRUE | 3406850.505 | 2175828.033 | 1.565772 |
| HEATR1 | 2 | 9 | 5.2 | 26 | 0 | 20.845 | 27948000 | 24565000 | 47991000 | 30595000 | 957540 | HEATR1 | 2325 | 47991000 | 30595000 | FALSE | FALSE | 47991000 | 30595000 | 1.56859 |
| BUD31 | 2 | 3 | 18.1 | 6 | 0.000434 | 3.3796 | 10328000 | 8604600 | 18176000 | 11560000 | 5366900 | BUD31 | 1379 | 18176000 | 11560000 | FALSE | FALSE | 18176000 | 11560000 | 1.572318 |
| PKP3 | 7 | 5 | 7.7 | 8 | 0 | 8.2499 | 0 | 0 | 26575000 | 16900000 | 765960 | PKP3 | 2420 | 26575000 | 16900000 | FALSE | FALSE | 26575000 | 16900000 | 1.572485 |
| CSTF1 | 3 | 6 | 19.3 | 11 | 0 | 16.832 | 22858000 | 24537000 | 29258000 | 18530000 | 4824800 | CSTF1 | 1446 | 29258000 | 18530000 | FALSE | FALSE | 29258000 | 18530000 | 1.578953 |
| MRPL14 | 1 | 2 | 13.1 | 4 | 0.000832 | 3.0142 | 0 | 6187900 | 11812000 | 7471600 | 2818400 | MRPL14 | 1782 | 11812000 | 7471600 | FALSE | FALSE | 11812000 | 7471600 | 1.58092 |
| PGRMC2 | 3 | 3 | 19.7 | 11 | 0 | 7.3071 | 19317000 | 16579000 | 9438800 | 5952900 | 5252400 | PGRMC2 | 1396 | 9438800 | 5952900 | FALSE | FALSE | 9438800 | 5952900 | 1.58558 |
| CHP1 | 6 | 2 | 22 | 0 | 0.003652 | 1.9639 | 0 | 10728000 | 0 | 0 | 3052100 | CHP1 | 1743 | 2261361.163 | 1424876.25 | TRUE | TRUE | 2261361.163 | 1424876.25 | 1.587058 |
| PRPF38B | 3 | 2 | 3.5 | 3 | 0.004301 | 1.8603 | 4793400 | 0 | 0 | 0 | 591670 | PRPF38B | 2526 | 2385155.48 | 1499139.545 | TRUE | TRUE | 2385155.48 | 1499139.545 | 1.591016 |
| CYFIP1;CY | 12 | 5 | 6.2 | 13 | 0 | 10.068 | 24391000 | 16971000 | 46332000 | 29069000 | 2272900 | CYFIP1 | 1900 | 46332000 | 29069000 | FALSE | FALSE | 46332000 | 29069000 | 1.593863 |
| EIF3G | 5 | 6 | 19.1 | 17 | 0 | 28.423 | 55047000 | 55228000 | 46529000 | 29108000 | 9364600 | EIF3G | 1082 | 46529000 | 29108000 | FALSE | FALSE | 46529000 | 29108000 | 1.598495 |
| UBA6 | 3 | 6 | 13.5 | 12 | 0 | 13.771 | 28591000 | 31270000 | 47734000 | 29846000 | 3666500 | UBA6 | 1619 | 47734000 | 29846000 | FALSE | FALSE | 47734000 | 29846000 | 1.599343 |
| PCBD1 | 1 | 2 | 22.1 | 3 | 0 | 7.2239 | 0 | 0 | 56393000 | 35227000 | 12321000 | PCBD1 | 945 | 56393000 | 35227000 | FALSE | FALSE | 56393000 | 35227000 | 1.600846 |
| PITPNA | 4 | 2 | 52 | 5 | 0 | 13.879 | 6663500 | 8794600 | 9280300 | 0 | 4686100 | PITPNA | 1467 | 9280300 | 5780914.793 | FALSE | TRUE | 9280300 | 5780914.793 | 1.605334 |
| GMDS | 2 | 5 | 19 | 10 | 0 | 12.012 | 12713000 | 22635000 | 27980000 | 17427000 | 2833500 | GMDS | 1778 | 27980000 | 17427000 | FALSE | FALSE | 27980000 | 17427000 | 1.605555 |
| NELFA | 11 | 2 | 8.2 | 3 | 0.000442 | 3.5566 | 0 | 0 | 16507000 | 10274000 | 1475300 | NELFA | 2124 | 16507000 | 10274000 | FALSE | FALSE | 16507000 | 10274000 | 1.606677 |
| SURF4 | 5 | 2 | 10.2 | 5 | 0.000444 | 3.606 | 29501000 | 26380000 | 23398000 | 14488000 | 9332400 | SURF4 | 1085 | 23398000 | 14488000 | FALSE | FALSE | 23398000 | 14488000 | 1.614992 |
| SRSF5 | 6 | 2 | 13.4 | 3 | 0 | 7.7839 | 0 | 10178000 | 0 | 0 | 4400900 | SRSF5 | 1520 | 3169997.84 | 1948888.677 | TRUE | TRUE | 3169997.84 | 1948888.677 | 1.626567 |
| PCYT1A | 14 | 3 | 19.4 | 7 | 0 | 5.1229 | 34285000 | 22690000 | 30392000 | 18682000 | 9226900 | PCYT1A | 1091 | 30392000 | 18682000 | FALSE | FALSE | 30392000 | 18682000 | 1.626807 |
| WDR75 | 1 | 2 | 3 | 5 | 0.000778 | 2.4816 | 3500000 | 2392500 | 7089100 | 4355100 | 337730 | WDR75 | 2692 | 7089100 | 4355100 | FALSE | FALSE | 7089100 | 4355100 | 1.62777 |
| RRAGC;RRA | 3 | 5 | 13 | 6 | 0 | 9.5185 | 26724000 | 16600000 | 0 | 0 | 2426900 | RRAGC | 1847 | 7409809.984 | 4540176.776 | TRUE | TRUE | 7409809.984 | 4540176.776 | 1.632053 |
| PROCR | 2 | 3 | 15.1 | 8 | 0 | 9.0508 | 14242000 | 7037700 | 26853000 | 16441000 | 5371800 | PROCR | 1378 | 26853000 | 16441000 | FALSE | FALSE | 26853000 | 16441000 | 1.633295 |
| MRPL47 | 3 | 6 | 26.5 | 11 | 0 | 10.56 | 18024000 | 18717000 | 24572000 | 14969000 | 5061300 | MRPL47 | 1413 | 24572000 | 14969000 | FALSE | FALSE | 24572000 | 14969000 | 1.641526 |
| TNKS1BP1 | 3 | 5 | 3.4 | 7 | 0 | 10.158 | 17131000 | 14089000 | 38214000 | 23263000 | 855400 | TNKS1BP1 | 2374 | 38214000 | 23263000 | FALSE | FALSE | 38214000 | 23263000 | 1.642694 |
| MTX2 | 4 | 2 | 11.1 | 4 | 0.000443 | 3.5729 | 0 | 8088200 | 0 | 0 | 2110100 | MTX2 | 1937 | 7287016.55 | 4410949.09 | TRUE | TRUE | 7287016.55 | 4410949.09 | 1.652029 |
| WDR61 | 7 | 4 | 25.9 | 8 | 0 | 19.355 | 9406900 | 11479000 | 20497000 | 12375000 | 3236500 | WDR61 | 1707 | 20497000 | 12375000 | FALSE | FALSE | 20497000 | 12375000 | 1.656323 |
| GDA | 4 | 5 | 13.2 | 8 | 0 | 15.629 | 0 | 0 | 22738000 | 13692000 | 1151400 | GDA | 2247 | 22738000 | 13692000 | FALSE | FALSE | 22738000 | 13692000 | 1.660678 |
| ERO1L | 7 | 11 | 32.1 | 35 | 0 | 62.356 | 106880000 | 108360000 | 83516000 | 50289000 | 13150000 | ERO1L | 916 | 83516000 | 50289000 | FALSE | FALSE | 83516000 | 50289000 | 1.660721 |
| MYO18A | 6 | 6 | 3.4 | 6 | 0 | 8.258 | 0 | 10100000 | 23859000 | 14353000 | 414670 | MYO18A | 2637 | 23859000 | 14353000 | FALSE | FALSE | 23859000 | 14353000 | 1.662301 |
| EIF3M | 6 | 6 | 38.7 | 18 | 0 | 54.875 | 48211000 | 51488000 | 77274000 | 46464000 | 19769000 | EIF3M | 716 | 77274000 | 46464000 | FALSE | FALSE | 77274000 | 46464000 | 1.663094 |
| PPP2R5D;F | 13 | 2 | 4.8 | 6 | 0 | 5.3568 | 7847700 | 8721500 | 11735000 | 7047700 | 1291800 | PPP2R5D | 2184 | 11735000 | 7047700 | FALSE | FALSE | 11735000 | 7047700 | 1.665082 |
| ANXA10 | 1 | 3 | 11.4 | 4 | 0 | 58.45 | 0 | 0 | 45318000 | 27043000 | 3287300 | ANXA10 | 1694 | 45318000 | 27043000 | FALSE | FALSE | 45318000 | 27043000 | 1.675776 |

| | | | | | | | | | | | | | | | | | | | | |
|---|---|---|---|---|---|---|---|---|---|---|---|---|---|---|---|---|---|---|---|---|
| PSMG1 | 3 | 4 | 17.6 | 6 | 0 | 14.532 | 15989000 | 16285000 | 34515000 | 20551000 | 5089400 | PSMG1 | 1409 | 34515000 | 20551000 | FALSE | FALSE | 34515000 | 20551000 | 1.67948 |
| HCFC1 | 6 | 6 | 3.5 | 8 | 0 | 13.359 | 0 | 6791900 | 17041000 | 10109000 | 508140 | HCFC1 | 2576 | 17041000 | 10109000 | FALSE | FALSE | 17041000 | 10109000 | 1.685726 |
| MRPS23 | 2 | 3 | 21.1 | 7 | 0 | 7.056 | 16877000 | 18875000 | 25901000 | 15327000 | 7558600 | MRPS23 | 1209 | 25901000 | 15327000 | FALSE | FALSE | 25901000 | 15327000 | 1.689894 |
| CTBP2 | 6 | 3 | 8.6 | 8 | 0 | 6.0065 | 51747000 | 46014000 | 75162000 | 44381000 | 8563400 | CTBP2 | 1134 | 75162000 | 44381000 | FALSE | FALSE | 75162000 | 44381000 | 1.693563 |
| BCKDHA | 4 | 2 | 9.1 | 4 | 0 | 7.158 | 0 | 0 | 12366000 | 7301300 | 1110500 | BCKDHA | 2261 | 12366000 | 7301300 | FALSE | FALSE | 12366000 | 7301300 | 1.693671 |
| SMC3 | 1 | 7 | 8.4 | 8 | 0 | 13.455 | 0 | 7952500 | 31803000 | 18739000 | 752380 | SMC3 | 2429 | 31803000 | 18739000 | FALSE | FALSE | 31803000 | 18739000 | 1.697156 |
| MT-ND4 | 1 | 2 | 4.8 | 1 | 0.004633 | 1.8117 | 7439000 | 0 | 0 | 0 | 1450600 | MT-ND4 | 2134 | 3364686.193 | 1976177.952 | TRUE | TRUE | 3364686.193 | 1976177.952 | 1.702623 |
| PPP6C | 3 | 3 | 10.6 | 6 | 0 | 6.2633 | 7546600 | 9422400 | 8093100 | 0 | 1726600 | PPP6C | 2042 | 8093100 | 4731869.889 | FALSE | TRUE | 8093100 | 4731869.889 | 1.710339 |
| ITGA3 | 3 | 6 | 5.5 | 7 | 0 | 10.963 | 22327000 | 45507000 | 0 | 0 | 1762100 | ITGA3 | 2030 | 2643294.08 | 1544539.906 | TRUE | TRUE | 2643294.08 | 1544539.906 | 1.71138 |
| GFPT2 | 2 | 2 | 6.2 | 1 | 0.000786 | 2.55 | 9227400 | 0 | 0 | 0 | 317000 | GFPT2 | 2702 | 4139524.098 | 2403000.141 | TRUE | TRUE | 4139524.098 | 2403000.141 | 1.722648 |
| HIST1H1C | 2 | 3 | 44.1 | 3 | 0 | 6.2664 | 536660000 | 160800000 | 230970000 | 133990000 | 95794000 | HIST1H1C | 239 | 230970000 | 133990000 | FALSE | FALSE | 230970000 | 133990000 | 1.723785 |
| PCCB | 11 | 3 | 10.9 | 3 | 0 | 5.5025 | 0 | 0 | 7665400 | 0 | 471700 | PCCB | 2602 | 7665400 | 4442426.307 | FALSE | TRUE | 7665400 | 4442426.307 | 1.725499 |
| CYB5B | 5 | 5 | 59.3 | 21 | 0 | 38.113 | 40074000 | 50646000 | 78195000 | 45120000 | 30797000 | CYB5B | 526 | 78195000 | 45120000 | FALSE | FALSE | 78195000 | 45120000 | 1.733045 |
| TUBG1;TUE | 4 | 4 | 20 | 11 | 0 | 18.878 | 9114100 | 9078900 | 21720000 | 12511000 | 4546700 | TUBG1 | 1499 | 21720000 | 12511000 | FALSE | FALSE | 21720000 | 12511000 | 1.736072 |
| ALG3 | 7 | 3 | 9.9 | 10 | 0 | 9.0567 | 17014000 | 10455000 | 20986000 | 11873000 | 3658400 | ALG3 | 1621 | 20986000 | 11873000 | FALSE | FALSE | 20986000 | 11873000 | 1.76754 |
| AP3D1 | 8 | 5 | 6.4 | 5 | 0 | 7.6658 | 17996000 | 15346000 | 29041000 | 16356000 | 1430000 | AP3D1 | 2144.5 | 29041000 | 16356000 | FALSE | FALSE | 29041000 | 16356000 | 1.775556 |
| RPL38 | 4 | 4 | 39.1 | 34 | 0 | 18.54 | 150930000 | 146610000 | 272880000 | 153380000 | 206760000 | RPL38 | 112 | 272880000 | 153380000 | FALSE | FALSE | 272880000 | 153380000 | 1.779111 |
| MOGS | 6 | 3 | 7.5 | 5 | 0 | 8.4508 | 26483000 | 22035000 | 45164000 | 25335000 | 3541500 | MOGS | 1645 | 45164000 | 25335000 | FALSE | FALSE | 45164000 | 25335000 | 1.782672 |
| LTA4H | 5 | 17 | 38.8 | 40 | 0 | 69.916 | 32667000 | 44729000 | 294580000 | 165150000 | 11773000 | LTA4H | 963 | 294580000 | 165150000 | FALSE | FALSE | 294580000 | 165150000 | 1.783712 |
| CISD2 | 4 | 3 | 30.4 | 1 | 0 | 5.5626 | 0 | 16134000 | 0 | 0 | 7775000 | CISD2 | 1187 | 5785969.108 | 3243048.707 | TRUE | TRUE | 5785969.108 | 3243048.707 | 1.784114 |
| UBE2K | 4 | 6 | 39 | 13 | 0 | 9.4026 | 24464000 | 26331000 | 52330000 | 29127000 | 9168900 | UBE2K | 1094 | 52330000 | 29127000 | FALSE | FALSE | 52330000 | 29127000 | 1.796615 |
| ACAD8 | 3 | 2 | 8.3 | 3 | 0 | 5.5333 | 0 | 0 | 13677000 | 7591600 | 809390 | ACAD8 | 2398 | 13677000 | 7591600 | FALSE | FALSE | 13677000 | 7591600 | 1.801597 |
| NELFB | 2 | 2 | 4 | 1 | 0.001491 | 2.1706 | 0 | 0 | 7197400 | 0 | 342640 | NELFB | 2690 | 7197400 | 3972561.24 | FALSE | TRUE | 7197400 | 3972561.24 | 1.811778 |
| GCA | 3 | 2 | 13 | 4 | 0.000801 | 2.7326 | 0 | 0 | 14461000 | 7961000 | 1901400 | GCA | 1988 | 14461000 | 7961000 | FALSE | FALSE | 14461000 | 7961000 | 1.81648 |
| RCN3 | 3 | 4 | 14.9 | 4 | 0 | 13.753 | 0 | 10862000 | 0 | 0 | 975070 | RCN3 | 2322 | 6569196.746 | 3598670.984 | TRUE | TRUE | 6569196.746 | 3598670.984 | 1.825451 |
| MRPS31 | 1 | 4 | 13.4 | 9 | 0 | 15.587 | 7424100 | 11224000 | 13366000 | 7315800 | 1521800 | MRPS31 | 2109.5 | 13366000 | 7315800 | FALSE | FALSE | 13366000 | 7315800 | 1.827005 |
| GALE | 6 | 4 | 23.8 | 5 | 0 | 17.37 | 0 | 6477700 | 21702000 | 11856000 | 2815800 | GALE | 1784 | 21702000 | 11856000 | FALSE | FALSE | 21702000 | 11856000 | 1.830466 |
| SEC16A | 10 | 2 | 3.1 | 1 | 0.00257 | 2.0041 | 0 | 3403900 | 0 | 0 | 337540 | SEC16A | 2693 | 6779668.112 | 3702154.203 | TRUE | TRUE | 6779668.112 | 3702154.203 | 1.831277 |
| CLDN1 | 1 | 3 | 19 | 6 | 0 | 33.659 | 16318000 | 24972000 | 0 | 0 | 5294800 | CLDN1 | 1388 | 3781022.635 | 2064255.165 | TRUE | TRUE | 3781022.635 | 2064255.165 | 1.831664 |
| RND3;ARHE | 3 | 2 | 9.8 | 4 | 0 | 4.5354 | 4409800 | 6875900 | 0 | 0 | 757450 | RND3 | 2422 | 5618419.948 | 3065615.803 | TRUE | TRUE | 5618419.948 | 3065615.803 | 1.832721 |
| HEXA | 6 | 4 | 8.1 | 3 | 0.000455 | 3.8622 | 0 | 0 | 32990000 | 17877000 | 2277500 | HEXA | 1898 | 32990000 | 17877000 | FALSE | FALSE | 32990000 | 17877000 | 1.845388 |
| RANBP3 | 10 | 4 | 9.3 | 4 | 0 | 9.4666 | 10905000 | 0 | 0 | 0 | 1009000 | RANBP3 | 2305 | 2295496.822 | 1241375.781 | TRUE | TRUE | 2295496.822 | 1241375.781 | 1.849155 |
| RPS29 | 5 | 2 | 20.7 | 9 | 0 | 5.839 | 74246000 | 62982000 | 67927000 | 36639000 | 53649000 | RPS29 | 368 | 67927000 | 36639000 | FALSE | FALSE | 67927000 | 36639000 | 1.853953 |
| PTK2 | 16 | 5 | 4.9 | 6 | 0 | 15.771 | 10367000 | 9333200 | 23495000 | 12666000 | 818840 | PTK2 | 2391 | 23495000 | 12666000 | FALSE | FALSE | 23495000 | 12666000 | 1.854966 |
| UTP6 | 3 | 3 | 4.7 | 6 | 0 | 4.3662 | 3487900 | 0 | 10539000 | 5629300 | 454140 | UTP6 | 2614 | 10539000 | 5629300 | FALSE | FALSE | 10539000 | 5629300 | 1.872169 |
| ARHGEF1 | 10 | 6 | 8.5 | 8 | 0 | 14.015 | 11467000 | 0 | 37711000 | 20091000 | 1198900 | ARHGEF1 | 2229 | 37711000 | 20091000 | FALSE | FALSE | 37711000 | 20091000 | 1.87701 |
| CYP1B1 | 3 | 5 | 10.1 | 7 | 0 | 10.294 | 19449000 | 10464000 | 0 | 0 | 1000600 | CYP1B1 | 2311 | 3780585.875 | 1986970.243 | TRUE | TRUE | 3780585.875 | 1986970.243 | 1.902689 |
| PLD3 | 11 | 9 | 25.3 | 18 | 0 | 41.23 | 203840000 | 87646000 | 5656700 | 2947600 | 16632000 | PLD3 | 789 | 5656700 | 2947600 | FALSE | FALSE | 5656700 | 2947600 | 1.919087 |
| TRA2A | 4 | 3 | 26.5 | 3 | 0.000467 | 4.1828 | 11088000 | 10325000 | 25373000 | 13219000 | 8178400 | TRA2A | 1159 | 25373000 | 13219000 | FALSE | FALSE | 25373000 | 13219000 | 1.919434 |
| JMJD6 | 6 | 2 | 8.1 | 2 | 0 | 4.8269 | 8557900 | 0 | 5922000 | 0 | 1456500 | JMJD6 | 2131 | 5922000 | 3054445.726 | FALSE | TRUE | 5922000 | 3054445.726 | 1.938813 |
| LY6K | 4 | 2 | 13.3 | 4 | 0.000469 | 4.2219 | 8454100 | 0 | 0 | 0 | 1047200 | LY6K | 2291 | 3667739.673 | 1888702.965 | TRUE | TRUE | 3667739.673 | 1888702.965 | 1.941936 |
| NRBP1 | 2 | 2 | 4.8 | 6 | 0 | 5.1516 | 6736500 | 8930800 | 15354000 | 7866800 | 1853500 | NRBP1 | 2005 | 15354000 | 7866800 | FALSE | FALSE | 15354000 | 7866800 | 1.951747 |
| NDUFAF3 | 1 | 2 | 13.6 | 5 | 0 | 5.3955 | 0 | 10243000 | 0 | 0 | 1937000 | NDUFAF3 | 1979 | 6761561.672 | 3462764.3 | TRUE | TRUE | 6761561.672 | 3462764.3 | 1.952649 |
| CCDC80 | 3 | 2 | 15.3 | 2 | 0.008191 | 1.498 | 0 | 16781000 | 0 | 0 | 1940700 | CCDC80 | 1978 | 3845732.982 | 1959957.583 | TRUE | TRUE | 3845732.982 | 1959957.583 | 1.962151 |
| CPT2 | 6 | 7 | 10.9 | 9 | 0 | 13.413 | 15714000 | 14315000 | 44269000 | 22557000 | 2238200 | CPT2 | 1909 | 44269000 | 22557000 | FALSE | FALSE | 44269000 | 22557000 | 1.962539 |
| OSGEP | 2 | 3 | 12.5 | 5 | 0 | 7.2051 | 8089500 | 12178000 | 0 | 0 | 1156100 | OSGEP | 2246 | 6217408.877 | 3166266.749 | TRUE | TRUE | 6217408.877 | 3166266.749 | 1.96364 |
| ITPA | 3 | 5 | 36.1 | 18 | 0 | 15.601 | 73874000 | 80442000 | 133080000 | 67601000 | 30580000 | ITPA | 530 | 133080000 | 67601000 | FALSE | FALSE | 133080000 | 67601000 | 1.96861 |
| UFL1 | 3 | 3 | 6.7 | 4 | 0 | 4.2791 | 0 | 7885000 | 0 | 0 | 670800 | UFL1 | 2481 | 4543517.397 | 2291811.206 | TRUE | TRUE | 4543517.397 | 2291811.206 | 1.982501 |
| AKR1B1 | 3 | 15 | 56 | 74 | 0 | 87.26 | 2806600000 | 976170000 | 0 | 0 | 210910000 | AKR1B1 | 111 | 3807045.845 | 1913130.933 | TRUE | TRUE | 3807045.845 | 1913130.933 | 1.989956 |
| CHI3L1 | 2 | 3 | 23.7 | 3 | 0 | 4.9475 | 12022000 | 0 | 0 | 0 | 1335700 | CHI3L1 | 2175 | 1915076.379 | 958777.4917 | TRUE | TRUE | 1915076.379 | 958777.4917 | 1.997415 |
| AURKA;AUR | 19 | 2 | 5.5 | 1 | 0.000449 | 3.7271 | 8821700 | 0 | 0 | 0 | 383550 | AURKA | 2657 | 5812344.175 | 2889833.735 | TRUE | TRUE | 5812344.175 | 2889833.735 | 2.011307 |
| TBRG4 | 5 | 4 | 8.1 | 5 | 0 | 7.7808 | 21591000 | 21991000 | 46521000 | 23127000 | 3052400 | TBRG4 | 1742 | 46521000 | 23127000 | FALSE | FALSE | 46521000 | 23127000 | 2.011545 |
| UFC1 | 1 | 3 | 18 | 7 | 0 | 6.0391 | 26018000 | 16148000 | 0 | 0 | 5500800 | UFC1 | 1369 | 6210422.993 | 3056435.061 | TRUE | TRUE | 6210422.993 | 3056435.061 | 2.031917 |
| COPS3 | 6 | 2 | 9.6 | 2 | 0 | 11.23 | 9905800 | 0 | 0 | 0 | 813190 | COPS3 | 2394 | 4380812.367 | 2132453.39 | TRUE | TRUE | 4380812.367 | 2132453.39 | 2.054353 |
| PDCD10 | 8 | 2 | 11.8 | 7 | 0 | 5.3673 | 6480400 | 8738300 | 43866000 | 21301000 | 5019700 | PDCD10 | 1419 | 43866000 | 21301000 | FALSE | FALSE | 43866000 | 21301000 | 2.05934 |
| FITM2 | 1 | 2 | 8.4 | 2 | 0.004961 | 1.7548 | 6916100 | 0 | 0 | 0 | 1531700 | FITM2 | 2105 | 5662342.344 | 2731666.348 | TRUE | TRUE | 5662342.344 | 2731666.348 | 2.072853 |
| VAMP5 | 1 | 2 | 12.1 | 1 | 0.004911 | 1.7177 | 0 | 0 | 5324100 | 0 | 1464700 | VAMP5 | 2126 | 5324100 | 2567878.237 | FALSE | TRUE | 5324100 | 2567878.237 | 2.073346 |
| AAR2 | 2 | 2 | 5.5 | 3 | 0 | 4.8652 | 8214000 | 4277300 | 0 | 3615300 | 812790 | AAR2 | 2395 | 7500087.131 | 3615300 | TRUE | FALSE | 7500087.131 | 3615300 | 2.074541 |
| MCMBP | 3 | 3 | 5.8 | 4 | 0 | 4.4019 | 5700400 | 5515800 | 19411000 | 9252800 | 1355300 | MCMBP | 2169 | 19411000 | 9252800 | FALSE | FALSE | 19411000 | 9252800 | 2.097851 |
| MRPL4 | 7 | 2 | 27.6 | 5 | 0 | 8.0408 | 12113000 | 0 | 0 | 0 | 2335900 | MRPL4 | 1878 | 3495993.571 | 1647384.592 | TRUE | TRUE | 3495993.571 | 1647384.592 | 2.122148 |
| NDUFB4 | 4 | 2 | 23.3 | 3 | 0 | 5.6414 | 9618200 | 0 | 0 | 0 | 4890900 | NDUFB4 | 1433 | 2817925.003 | 1326130.714 | TRUE | TRUE | 2817925.003 | 1326130.714 | 2.124923 |
| CRK | 3 | 4 | 21.6 | 8 | 0 | 10.091 | 23769000 | 22241000 | 19631000 | 9208700 | 4590700 | CRK | 1490 | 19631000 | 9208700 | FALSE | FALSE | 19631000 | 9208700 | 2.131788 |
| ABHD10 | 3 | 5 | 20.9 | 9 | 0 | 17.663 | 10703000 | 13405000 | 24091000 | 11071000 | 2724200 | ABHD10 | 1793 | 24091000 | 11071000 | FALSE | FALSE | 24091000 | 11071000 | 2.176046 |
| PPAN;PPAN | 7 | 2 | 11.5 | 4 | 0 | 5.4885 | 3635600 | 0 | 0 | 0 | 520030 | PPAN | 2565 | 3890656.456 | 1771624.049 | TRUE | TRUE | 3890656.456 | 1771624.049 | 2.196096 |
| CASP6 | 4 | 2 | 14.7 | 3 | 0 | 5.3127 | 0 | 0 | 10989000 | 4956200 | 1528400 | CASP6 | 2107 | 10989000 | 4956200 | FALSE | FALSE | 10989000 | 4956200 | 2.217223 |
| CSK;MATK | 9 | 2 | 20.9 | 4 | 0.000781 | 2.5 | 4605800 | 5212700 | 13423000 | 5965700 | 4045900 | CSK | 1558 | 13423000 | 5965700 | FALSE | FALSE | 13423000 | 5965700 | 2.250029 |
| DRG2 | 8 | 3 | 10.5 | 2 | 0 | 7.3695 | 0 | 5163100 | 0 | 0 | 1022900 | DRG2 | 2299 | 2928436.964 | 1301011.873 | TRUE | TRUE | 2928436.964 | 1301011.873 | 2.250892 |
| APRT | 6 | 6 | 54.5 | 12 | 0 | 32.103 | 27734000 | 21866000 | 99598000 | 44234000 | 15085000 | APRT | 839 | 99598000 | 44234000 | FALSE | FALSE | 99598000 | 44234000 | 2.251616 |
| SDF2L1 | 1 | 2 | 14.9 | 2 | 0.000443 | 3.5854 | 15885000 | 0 | 0 | 0 | 2428500 | SDF2L1 | 1845 | 3965714.176 | 1760296.777 | TRUE | TRUE | 3965714.176 | 1760296.777 | 2.252867 |
| ATP6AP1 | 4 | 3 | 6.4 | 3 | 0.000803 | 2.7392 | 21842000 | 12971000 | 0 | 0 | 2030000 | ATP6AP1 | 1958 | 2769169.405 | 1226771.663 | TRUE | TRUE | 2769169.405 | 1226771.663 | 2.257282 |
| CFL2 | 3 | 3 | 38.3 | 4 | 0 | 14.613 | 15885000 | 24386000 | 0 | 0 | 4262300 | CFL2 | 1538 | 3605306.894 | 1594906.599 | TRUE | TRUE | 3605306.894 | 1594906.599 | 2.260513 |
| IL6ST | 5 | 2 | 2.8 | 2 | 0.001141 | 2.2986 | 6839200 | 0 | 0 | 0 | 208550 | IL6ST | 2791 | 5107084.299 | 2258537.96 | TRUE | TRUE | 5107084.299 | 2258537.96 | 2.261235 |
| SERINC1 | 1 | 4 | 10.8 | 7 | 0 | 10.275 | 49130000 | 30562000 | 0 | 0 | 4996500 | SERINC1 | 1422 | 3438669.047 | 1471385.358 | TRUE | TRUE | 3438669.047 | 1471385.358 | 2.337028 |
| RRAGB;RRA | 4 | 2 | 11.9 | 4 | 0 | 5.5907 | 15997000 | 10929000 | 6307200 | 0 | 3091800 | RRAGB | 1733 | 6307200 | 2696541.295 | FALSE | TRUE | 6307200 | 2696541.295 | 2.338996 |
| CMSS1 | 4 | 2 | 8.9 | 3 | 0.005567 | 1.6669 | 32067000 | 0 | 0 | 0 | 3512400 | CMSS1 | 1653 | 4980316.355 | 2073802.304 | TRUE | TRUE | 4980316.355 | 2073802.304 | 2.401539 |
| EHD2 | 2 | 5 | 12.2 | 12 | 0 | 16.386 | 45440000 | 29539000 | 0 | 0 | 2641200 | EHD2 | 1802 | 2972141.234 | 1235705.894 | TRUE | TRUE | 2972141.234 | 1235705.894 | 2.405217 |
| POLR2E | 5 | 2 | 17.2 | 2 | 0.001136 | 2.2736 | 0 | 21172000 | 0 | 0 | 4798100 | POLR2E | 1450 | 4245694.656 | 1757698.584 | TRUE | TRUE | 4245694.656 | 1757698.584 | 2.415485 |
| GINM1 | 2 | 2 | 9.1 | 2 | 0.003984 | 1.9103 | 6575000 | 0 | 0 | 0 | 657130 | GINM1 | 2488 | 4225375.235 | 1744683.136 | TRUE | TRUE | 4225375.235 | 1744683.136 | 2.421858 |
| NDRG3 | 6 | 2 | 6.8 | 3 | 0.004287 | 1.8458 | 0 | 0 | 2876000 | 0 | 638800 | NDRG3 | 2497 | 2876000 | 1182955.422 | FALSE | TRUE | 2876000 | 1182955.422 | 2.431199 |
| ABLIM1 | 14 | 2 | 16.9 | 2 | 0.000429 | 3.2901 | 0 | 0 | 5173200 | 0 | 480810 | ABLIM1 | 2595 | 5173200 | 2094395.485 | FALSE | TRUE | 5173200 | 2094395.485 | 2.470021 |
| CHMP1A | 4 | 3 | 13.8 | 2 | 0.000799 | 2.7147 | 12075000 | 0 | 0 | 0 | 4010200 | CHMP1A | 1562 | 4187293.202 | 1695072.668 | TRUE | TRUE | 4187293.202 | 1695072.668 | 2.470274 |
| TST | 2 | 5 | 23.2 | 9 | 0 | 22.974 | 0 | 17065000 | 124510000 | 50311000 | 9126100 | TST | 1097 | 124510000 | 50311000 | FALSE | FALSE | 124510000 | 50311000 | 2.474807 |
| ARMT1 | 2 | 2 | 4.8 | 1 | 0.000828 | 2.9836 | 0 | 0 | 6889000 | 0 | 1244800 | ARMT1 | 2205 | 6889000 | 2773953.594 | FALSE | TRUE | 6889000 | 2773953.594 | 2.483459 |
| GMFB | 2 | 3 | 29.3 | 4 | 0 | 5.7733 | 15571000 | 0 | 0 | 0 | 3822100 | GMFB | 1597 | 5397569.396 | 2147001.208 | TRUE | TRUE | 5397569.396 | 2147001.208 | 2.514004 |
| PARP4 | 1 | 3 | 1.8 | 3 | 0 | 5.1999 | 0 | 9794000 | 0 | 0 | 218560 | PARP4 | 2781 | 5607210.837 | 2213483.551 | TRUE | TRUE | 5607210.837 | 2213483.551 | 2.533206 |
| MAPK1 | 2 | 4 | 15.5 | 5 | 0 | 8.7507 | 0 | 7640900 | 20287000 | 7943200 | 1915200 | MAPK1 | 1982 | 20287000 | 7943200 | FALSE | FALSE | 20287000 | 7943200 | 2.554008 |
| PTTG1IP | 3 | 2 | 23.4 | 4 | 0 | 5.2244 | 23549000 | 0 | 0 | 0 | 4418200 | PTTG1IP | 1517 | 5266001.765 | 2053565.977 | TRUE | TRUE | 5266001.765 | 2053565.977 | 2.564321 |
| TMED1 | 3 | 2 | 9.7 | 3 | 0 | 4.9461 | 14522000 | 8821800 | 0 | 0 | 4287800 | TMED1 | 1533 | 4083961.258 | 1589988.617 | TRUE | TRUE | 4083961.258 | 1589988.617 | 2.568547 |
| HMGCL | 3 | 3 | 10.8 | 3 | 0.000446 | 3.6492 | 10897000 | 0 | 0 | 0 | 1511900 | HMGCL | 2112 | 4480411.97 | 1717423.589 | TRUE | TRUE | 4480411.97 | 1717423.589 | 2.608798 |
| OSMR | 3 | 2 | 1.5 | 4 | 0.001126 | 2.2183 | 0 | 8634000 | 0 | 0 | 400790 | OSMR | 2642 | 2200211.398 | 837150.69 | TRUE | TRUE | 2200211.398 | 837150.69 | 2.628214 |
| BCAR1 | 16 | 2 | 3.9 | 2 | 0.000435 | 3.3868 | 0 | 13816000 | 0 | 0 | 658980 | BCAR1 | 2486 | 6173970.511 | 2315513.423 | TRUE | TRUE | 6173970.511 | 2315513.423 | 2.666351 |
| TMX2 | 2 | 2 | 13.6 | 1 | 0.001158 | 2.4162 | 0 | 4520600 | 0 | 0 | 802770 | TMX2 | 2402 | 2990903.181 | 1119862.048 | TRUE | TRUE | 2990903.181 | 1119862.048 | 2.670778 |
| CPSF7 | 7 | 2 | 5.3 | 2 | 0.00113 | 2.2404 | 0 | 0 | 7432100 | 0 | 902090 | CPSF7 | 2351 | 7432100 | 2773447.393 | FALSE | TRUE | 7432100 | 2773447.393 | 2.679734 |
| ACAP2 | 5 | 2 | 3.2 | 3 | 0.000436 | 3.4352 | 3718900 | 5819600 | 0 | 0 | 302100 | ACAP2 | 2721 | 4903910.855 | 1822424.241 | TRUE | TRUE | 4903910.855 | 1822424.241 | 2.690872 |
| PEA15 | 3 | 2 | 22.3 | 5 | 0.00047 | 4.2397 | 9335700 | 8743200 | 5794600 | 0 | 3471900 | PEA15 | 1661 | 5794600 | 2152349.196 | FALSE | TRUE | 5794600 | 2152349.196 | 2.692221 |
| MACF1 | 12 | 6 | 2.1 | 12 | 0 | 16.297 | 14927000 | 14946000 | 12529000 | 0 | 163890 | MACF1 | 2826 | 12529000 | 4591929.817 | FALSE | TRUE | 12529000 | 4591929.817 | 2.728482 |
| CLPTM1L | 3 | 2 | 6 | 4 | 0.000454 | 3.8471 | 6250700 | 3935700 | 0 | 0 | 689720 | CLPTM1L | 2468 | 4239694.304 | 1548370.831 | TRUE | TRUE | 4239694.304 | 1548370.831 | 2.738165 |
| ALDH1A1 | 4 | 12 | 34.1 | 19 | 0 | 55.205 | 0 | 35786000 | 188010000 | 68620000 | 9822400 | ALDH1A1 | 1055 | 188010000 | 68620000 | FALSE | FALSE | 188010000 | 68620000 | 2.739872 |
| RHOG | 1 | 2 | 22.5 | 4 | 0 | 9.48 | 0 | 0 | 8491400 | 0 | 901650 | RHOG | 2352 | 8491400 | 3088971.706 | FALSE | TRUE | 8491400 | 3088971.706 | 2.748941 |
| UBE2H | 5 | 2 | 48.4 | 4 | 0.000427 | 3.2602 | 0 | 0 | 3428100 | 0 | 8047000 | UBE2H | 1167 | 3428100 | 1240283.036 | FALSE | TRUE | 3428100 | 1240283.036 | 2.763966 |
| DNAAF5 | 5 | 2 | 2.7 | 4 | 0.000471 | 4.2525 | 6025000 | 0 | 0 | 0 | 473740 | DNAAF5 | 2600 | 3405591.896 | 1229384.73 | TRUE | TRUE | 3405591.896 | 1229384.73 | 2.77016 |
| NOL6 | 5 | 2 | 3.1 | 3 | 0.004958 | 1.7514 | 8328900 | 4999600 | 0 | 0 | 416970 | NOL6 | 2636 | 5037415.062 | 1811755.582 | TRUE | TRUE | 5037415.062 | 1811755.582 | 2.780405 |
| TMEM59 | 5 | 6 | 20.4 | 9 | 0 | 14.579 | 71200000 | 35618000 | 0 | 0 | 7886000 | TMEM59 | 1175 | 3275425.026 | 1157990.365 | TRUE | TRUE | 3275425.026 | 1157990.365 | 2.828543 |
| CUL1 | 2 | 4 | 6.8 | 4 | 0 | 6.2575 | 5741100 | 0 | 0 | 0 | 584060 | CUL1 | 2529 | 2600528.624 | 916323.5955 | TRUE | TRUE | 2600528.624 | 916323.5955 | 2.838002 |
| PPP4R1 | 7 | 3 | 3 | 5 | 0.000451 | 3.772 | 15281000 | 9048600 | 103030000 | 35692000 | 3220200 | PPP4R1 | 1710 | 103030000 | 35692000 | FALSE | FALSE | 103030000 | 35692000 | 2.886641 |
| TTI1 | 1 | 2 | 1.9 | 3 | 0.00081 | 2.819 | 0 | 8471900 | 0 | 0 | 568090 | TTI1 | 2538 | 5224683.514 | 1809264.67 | TRUE | TRUE | 5224683.514 | 1809264.67 | 2.887739 |
| KIF5C | 3 | 2 | 11.6 | 2 | 0 | 4.8325 | 2417800 | 5954300 | 0 | 0 | 162240 | KIF5C | 2830 | 6785801.569 | 2344062.565 | TRUE | TRUE | 6785801.569 | 2344062.565 | 2.894889 |
| DHODH | 5 | 3 | 8.4 | 2 | 0.000457 | 3.9005 | 0 | 4775500 | 0 | 0 | 1504000 | DHODH | 2114 | 7204728.917 | 2450780.322 | TRUE | TRUE | 7204728.917 | 2450780.322 | 2.939769 |
| RAB32 | 4 | 6 | 33.8 | 9 | 0 | 20.201 | 22930000 | 36707000 | 0 | 0 | 4575100 | RAB32 | 1494.5 | 5440763.719 | 1833469.762 | TRUE | TRUE | 5440763.719 | 1833469.762 | 2.967468 |
| NCDN | 4 | 2 | 3.4 | 0 | 0.000429 | 3.2959 | 0 | 7186900 | 0 | 0 | 535020 | NCDN | 2554 | 2897606.458 | 972985.0431 | TRUE | TRUE | 2897606.458 | 972985.0431 | 2.978059 |
| AP3S1 | 2 | 2 | 13.5 | 3 | 0 | 4.5675 | 8390700 | 0 | 0 | 0 | 2244400 | AP3S1 | 1908 | 3281793.419 | 1089080.692 | TRUE | TRUE | 3281793.419 | 1089080.692 | 3.013361 |
| UBE2A | 6 | 2 | 22.7 | 7 | 0 | 6.8202 | 15990000 | 13299000 | 9444700 | 0 | 9912100 | UBE2A | 1051 | 9444700 | 3134152.783 | FALSE | TRUE | 9444700 | 3134152.783 | 3.013478 |
| ADPRHL2 | 1 | 3 | 10.7 | 8 | 0 | 8.8945 | 9183700 | 7276900 | 8909900 | 0 | 1762700 | ADPRHL2 | 2029 | 8909900 | 2938596.626 | FALSE | TRUE | 8909900 | 2938596.626 | 3.032026 |
| MRPS7 | 5 | 4 | 13.3 | 7 | 0 | 10.767 | 18384000 | 19708000 | 22556000 | 0 | 5514600 | MRPS7 | 1366 | 22556000 | 7404923.526 | FALSE | TRUE | 22556000 | 7404923.526 | 3.046081 |
| QTRTD1 | 4 | 2 | 6.8 | 3 | 0.000445 | 3.6313 | 0 | 0 | 8878000 | 0 | 1129700 | QTRTD1 | 2256 | 8878000 | 2910751.101 | FALSE | TRUE | 8878000 | 2910751.101 | 3.050072 |
| CDC45 | 3 | 2 | 4 | 2 | 0.000824 | 2.9503 | 0 | 0 | 7929300 | 0 | 269040 | CDC45 | 2741 | 7929300 | 2555690.635 | FALSE | TRUE | 7929300 | 2555690.635 | 3.102606 |
| LAS1L | 4 | 4 | 8.5 | 7 | 0 | 8.9421 | 13136000 | 15760000 | 11792000 | 0 | 1085700 | LAS1L | 2274 | 11792000 | 3712076.896 | FALSE | TRUE | 11792000 | 3712076.896 | 3.176658 |
| NDUFAF4 | 1 | 2 | 10.9 | 3 | 0 | 10.519 | 0 | 9495300 | 0 | 0 | 1098500 | NDUFAF4 | 2271 | 3416771.356 | 1073008.732 | TRUE | TRUE | 3416771.356 | 1073008.732 | 3.18429 |
| MAP1B | 5 | 15 | 8.6 | 16 | 0 | 54.799 | 43964000 | 14274000 | 0 | 0 | 577540 | MAP1B | 2535 | 7910095.79 | 2472494.17 | TRUE | TRUE | 7910095.79 | 2472494.17 | 3.199237 |
| H1FX | 1 | 2 | 11.7 | 3 | 0.000455 | 3.8647 | 0 | 13324000 | 0 | 0 | 3899000 | H1FX | 1584 | 5243400.903 | 1631278.792 | TRUE | TRUE | 5243400.903 | 1631278.792 | 3.214289 |
| PTGR1 | 3 | 2 | 10.3 | 4 | 0 | 7.5292 | 0 | 0 | 7145700 | 0 | 1278500 | PTGR1 | 2190 | 7145700 | 2221815.966 | FALSE | TRUE | 7145700 | 2221815.966 | 3.216153 |
| DST | 13 | 3 | 0.7 | 2 | 0.000417 | 3.0622 | 0 | 11861000 | 0 | 0 | 78590 | DST | 2897 | 4796847.286 | 1491281.736 | TRUE | TRUE | 4796847.286 | 1491281.736 | 3.216594 |
| NT5C3A | 7 | 3 | 10.9 | 3 | 0 | 5.1478 | 0 | 0 | 8680600 | 0 | 555010 | NT5C3A | 2545 | 8680600 | 2684618.815 | FALSE | TRUE | 8680600 | 2684618.815 | 3.233457 |
| GTF2F2 | 1 | 2 | 11.2 | 3 | 0.002226 | 2.1193 | 0 | 0 | 4252000 | 0 | 613820 | GTF2F2 | 2509 | 4252000 | 1308654.642 | FALSE | TRUE | 4252000 | 1308654.642 | 3.249138 |
| C11orf68 | 3 | 3 | 12.4 | 3 | 0.000432 | 3.3469 | 0 | 0 | 3686700 | 0 | 1031200 | C11orf68 | 2296 | 3686700 | 1128982.946 | FALSE | TRUE | 3686700 | 1128982.946 | 3.265505 |
| PLP2 | 2 | 2 | 18.4 | 9 | 0 | 16.867 | 54606000 | 33696000 | 0 | 0 | 25699000 | PLP2 | 601 | 2706722.76 | 828434.3114 | TRUE | TRUE | 2706722.76 | 828434.3114 | 3.267275 |
| SCCPDH | 1 | 2 | 6.8 | 3 | 0.000466 | 4.1572 | 6716200 | 0 | 0 | 0 | 580700 | SCCPDH | 2533 | 7187862.797 | 2184715.393 | TRUE | TRUE | 7187862.797 | 2184715.393 | 3.290068 |
| NAGK | 8 | 3 | 44.7 | 7 | 0 | 5.7887 | 10881000 | 9343000 | 7239300 | 0 | 5400000 | NAGK | 1377 | 7239300 | 2194911.125 | FALSE | TRUE | 7239300 | 2194911.125 | 3.29822 |
| GNE | 5 | 2 | 3.5 | 4 | 0 | 5.142 | 0 | 7596200 | 0 | 0 | 391650 | GNE | 2650 | 2672087.532 | 809622.6781 | TRUE | TRUE | 2672087.532 | 809622.6781 | 3.300411 |
| CHMP5 | 2 | 2 | 13.5 | 5 | 0.000447 | 3.6684 | 19324000 | 0 | 0 | 0 | 3938200 | CHMP5 | 1578 | 6393485.686 | 1901431.785 | TRUE | TRUE | 6393485.686 | 1901431.785 | 3.362459 |
| CDC123 | 4 | 3 | 25.5 | 1 | 0 | 5.4395 | 4815200 | 0 | 0 | 0 | 1458200 | CDC123 | 2130 | 3510725.694 | 1043981.167 | TRUE | TRUE | 3510725.694 | 1043981.167 | 3.362825 |
| KIF15 | 6 | 2 | 2.2 | 1 | 0.000819 | 2.9004 | 6750000 | 0 | 0 | 0 | 248210 | KIF15 | 2762 | 3945138.882 | 1154423.97 | TRUE | TRUE | 3945138.882 | 1154423.97 | 3.417409 |
| SLC30A1 | 1 | 2 | 6.1 | 2 | 0 | 7.2398 | 13287000 | 0 | 0 | 0 | 1006800 | SLC30A1 | 2307 | 3058476.481 | 880324.7277 | TRUE | TRUE | 3058476.481 | 880324.7277 | 3.474259 |
| SMC4 | 3 | 7 | 5.8 | 9 | 0 | 11.522 | 25014000 | 24251000 | 16834000 | 0 | 966880 | SMC4 | 2324 | 16834000 | 4844013.841 | FALSE | TRUE | 16834000 | 4844013.841 | 3.475217 |
| SNX6 | 4 | 5 | 15.6 | 5 | 0 | 7.5481 | 18870000 | 31593000 | 0 | 0 | 2518700 | SNX6 | 1828 | 3678268.176 | 1057507.527 | TRUE | TRUE | 3678268.176 | 1057507.527 | 3.478243 |
| PICALM;SN | 20 | 4 | 7.2 | 7 | 0 | 8.8606 | 9204200 | 12083000 | 14782000 | 0 | 1757100 | PICALM | 2032 | 14782000 | 4217962.648 | FALSE | TRUE | 14782000 | 4217962.648 | 3.504536 |
| CCDC51 | 3 | 3 | 15.2 | 2 | 0.000467 | 4.1841 | 0 | 8463100 | 0 | 0 | 895690 | CCDC51 | 2354 | 4852224.516 | 1382741.897 | TRUE | TRUE | 4852224.516 | 1382741.897 | 3.509132 |
| RPL7L1 | 3 | 2 | 12.1 | 4 | 0 | 7.2335 | 0 | 0 | 7351800 | 0 | 1751800 | RPL7L1 | 2034 | 7351800 | 2079304.251 | FALSE | TRUE | 7351800 | 2079304.251 | 3.535702 |
| HGS | 3 | 2 | 8 | 2 | 0.000424 | 3.1877 | 0 | 7005700 | 0 | 0 | 1233400 | HGS | 2209 | 4677633.634 | 1296646.239 | TRUE | TRUE | 4677633.634 | 1296646.239 | 3.607486 |
| PEF1 | 1 | 2 | 8.5 | 3 | 0 | 4.4063 | 6590900 | 6621800 | 7439400 | 0 | 2437900 | PEF1 | 1844 | 7439400 | 2034022.609 | FALSE | TRUE | 7439400 | 2034022.609 | 3.657481 |

| | | | | | | | | | | | | | | | | | | | | |
|---|---|---|---|---|---|---|---|---|---|---|---|---|---|---|---|---|---|---|---|---|
| ECHDC1 | 14 | 2 | 31.6 | 3 | 0.000426 | 3.2353 | 8541100 | 8679600 | 7318700 | 0 | 6315500 | ECHDC1 | 1304 | 7318700 | 1972174.738 | FALSE | TRUE | 7318700 | 1972174.738 | 3.710979 |
| GPS1 | 17 | 3 | 6.6 | 3 | 0.000811 | 2.8223 | 11418000 | 12151000 | 7327100 | 0 | 1902300 | GPS1 | 1986 | 7327100 | 1923808.576 | FALSE | TRUE | 7327100 | 1923808.576 | 3.808643 |
| DNAJA3 | 3 | 2 | 7.3 | 3 | 0 | 4.5075 | 10247000 | 0 | 0 | 0 | 975790 | DNAJA3 | 2321 | 6706484.506 | 1760381.832 | TRUE | TRUE | 6706484.506 | 1760381.832 | 3.809676 |
| HSDL2 | 2 | 4 | 14.2 | 2 | 0 | 17.146 | 0 | 25158000 | 0 | 0 | 3697300 | HSDL2 | 1612 | 6034400.953 | 1582821.406 | TRUE | TRUE | 6034400.953 | 1582821.406 | 3.812433 |
| RBM47 | 22 | 3 | 19.8 | 3 | 0 | 4.8831 | 0 | 0 | 6767000 | 0 | 510770 | RBM47 | 2575 | 6767000 | 1774871.81 | FALSE | TRUE | 6767000 | 1774871.81 | 3.81267 |
| CD97 | 3 | 2 | 3.5 | 3 | 0 | 6.3207 | 7183200 | 0 | 0 | 0 | 447420 | CD97 | 2618 | 3153879.487 | 813092.117 | TRUE | TRUE | 3153879.487 | 813092.117 | 3.878871 |
| CD3EAP | 2 | 2 | 6.7 | 3 | 0.003662 | 1.9766 | 6109000 | 7162600 | 6485400 | 0 | 1139900 | CD3EAP | 2251 | 6485400 | 1668912.941 | FALSE | TRUE | 6485400 | 1668912.941 | 3.886003 |
| PAWR | 3 | 2 | 8.8 | 4 | 0 | 6.5532 | 4177800 | 7212600 | 0 | 0 | 1381500 | PAWR | 2165 | 5884252.539 | 1500453.815 | TRUE | TRUE | 5884252.539 | 1500453.815 | 3.921649 |
| DLG1;DLG4 | 35 | 2 | 4.9 | 2 | 0.000793 | 2.646 | 0 | 0 | 6367400 | 0 | 1305200 | DLG1 | 2178 | 6367400 | 1620725.033 | FALSE | TRUE | 6367400 | 1620725.033 | 3.928736 |
| ARL1 | 6 | 3 | 17.5 | 6 | 0 | 6.3129 | 15425000 | 15146000 | 33273000 | 8361700 | 6790900 | ARL1 | 1267 | 33273000 | 8361700 | FALSE | FALSE | 33273000 | 8361700 | 3.979215 |
| C1orf123 | 1 | 2 | 25 | 5 | 0 | 5.0947 | 0 | 0 | 22467000 | 0 | 3079800 | C1orf123 | 1738 | 22467000 | 5602994.209 | FALSE | TRUE | 22467000 | 5602994.209 | 4.00982 |
| WDR77 | 3 | 2 | 6.8 | 4 | 0.005552 | 1.6514 | 0 | 16908000 | 0 | 0 | 6047600 | WDR77 | 1325 | 3970662.603 | 944197.7839 | TRUE | TRUE | 3970662.603 | 944197.7839 | 4.205329 |
| TM9SF4 | 4 | 3 | 7.2 | 8 | 0 | 12.719 | 27951000 | 23937000 | 11512000 | 0 | 2972300 | TM9SF4 | 1757 | 11512000 | 2726732.427 | FALSE | TRUE | 11512000 | 2726732.427 | 4.221903 |
| EXOSC3 | 2 | 3 | 18.2 | 5 | 0 | 12.024 | 7930400 | 9335600 | 9835200 | 0 | 2040700 | EXOSC3 | 1953 | 9835200 | 2317415.614 | FALSE | TRUE | 9835200 | 2317415.614 | 4.244038 |
| NPLOC4 | 3 | 3 | 5.3 | 1 | 0 | 5.1085 | 0 | 17072000 | 0 | 0 | 1406300 | NPLOC4 | 2157 | 4829439.012 | 1126971.11 | TRUE | TRUE | 4829439.012 | 1126971.11 | 4.285326 |
| TTC37 | 2 | 2 | 4.1 | 1 | 0.002932 | 1.9891 | 0 | 0 | 4660100 | 0 | 218080 | TTC37 | 2783 | 4660100 | 1086610.918 | FALSE | TRUE | 4660100 | 1086610.918 | 4.288656 |
| ARMCX3 | 2 | 4 | 11.9 | 2 | 0 | 7.5087 | 0 | 15568000 | 0 | 0 | 2282000 | ARMCX3 | 1896 | 5468862.734 | 1264064.133 | TRUE | TRUE | 5468862.734 | 1264064.133 | 4.326412 |
| YBX3 | 4 | 3 | 27.8 | 4 | 0 | 5.8656 | 13620000 | 23159000 | 11867000 | 0 | 5590300 | YBX3 | 1362 | 11867000 | 2710046.658 | FALSE | TRUE | 11867000 | 2710046.658 | 4.378891 |
| FAM134C | 6 | 2 | 30 | 1 | 0.000452 | 3.7793 | 4479100 | 0 | 0 | 0 | 1415600 | FAM134C | 2152 | 3729683.731 | 848107.1991 | TRUE | TRUE | 3729683.731 | 848107.1991 | 4.397656 |
| ABHD14A-A | 9 | 2 | 4.6 | 2 | 0.000802 | 2.7361 | 0 | 0 | 8384600 | 0 | 309220 | ABHD14A-A | 2713 | 8384600 | 1871379.637 | FALSE | TRUE | 8384600 | 1871379.637 | 4.480438 |
| IFNGR1 | 7 | 6 | 16.5 | 16 | 0 | 16.703 | 121390000 | 72275000 | 0 | 0 | 11478000 | IFNGR1 | 976 | 4414361.368 | 983929.1527 | TRUE | TRUE | 4414361.368 | 983929.1527 | 4.486463 |
| MXRA7 | 5 | 2 | 12.4 | 2 | 0 | 6.0075 | 11805000 | 7027900 | 0 | 0 | 3571900 | MXRA7 | 1639 | 4278350.067 | 945950.8875 | TRUE | TRUE | 4278350.067 | 945950.8875 | 4.522804 |
| TIMM9 | 5 | 2 | 29.2 | 11 | 0 | 11.766 | 22441000 | 31366000 | 21498000 | 0 | 12635000 | TIMM9 | 936 | 21498000 | 4731134.926 | FALSE | TRUE | 21498000 | 4731134.926 | 4.543941 |
| UBE2S | 2 | 2 | 13.2 | 3 | 0.000457 | 3.8966 | 8243700 | 4949300 | 0 | 0 | 1103900 | UBE2S | 2268 | 5119759.924 | 1105235.546 | TRUE | TRUE | 5119759.924 | 1105235.546 | 4.632279 |
| STXBP2 | 6 | 2 | 6.9 | 4 | 0 | 6.9946 | 0 | 0 | 12426000 | 0 | 649180 | STXBP2 | 2492 | 12426000 | 2661393.921 | FALSE | TRUE | 12426000 | 2661393.921 | 4.668982 |
| RMDN1 | 11 | 2 | 6.9 | 1 | 0.001163 | 2.4525 | 0 | 0 | 5685300 | 0 | 940250 | RMDN1 | 2333 | 5685300 | 1213877.035 | FALSE | TRUE | 5685300 | 1213877.035 | 4.683588 |
| RBM27;RBM | 8 | 2 | 2.2 | 3 | 0.000451 | 3.7596 | 3500700 | 0 | 0 | 0 | 212600 | RBM27 | 2787 | 12508300.41 | 2654957.42 | TRUE | TRUE | 12508300.41 | 2654957.42 | 4.7113 |
| ASMTL | 3 | 2 | 5.3 | 3 | 0 | 5.0054 | 5784100 | 9545900 | 0 | 0 | 737630 | ASMTL | 2442 | 8955568.398 | 1858272.466 | TRUE | TRUE | 8955568.398 | 1858272.466 | 4.819298 |
| STUB1 | 5 | 2 | 10.8 | 2 | 0.006226 | 1.6294 | 0 | 0 | 9390300 | 0 | 1521200 | STUB1 | 2111 | 9390300 | 1940956.383 | FALSE | TRUE | 9390300 | 1940956.383 | 4.837976 |
| SKIV2L | 2 | 2 | 1.8 | 2 | 0.002209 | 2.0316 | 0 | 0 | 4591300 | 0 | 63843 | SKIV2L | 2905 | 4591300 | 945638.8276 | FALSE | TRUE | 4591300 | 945638.8276 | 4.855236 |
| QSOX2 | 2 | 3 | 11.6 | 3 | 0 | 5.4671 | 0 | 12857000 | 9307300 | 0 | 1683500 | QSOX2 | 2055 | 9307300 | 1898484.204 | FALSE | TRUE | 9307300 | 1898484.204 | 4.90249 |
| MTOR | 1 | 2 | 1 | 4 | 0 | 4.7243 | 5125500 | 3821000 | 5740500 | 0 | 114790 | MTOR | 2878 | 5740500 | 1159447.375 | FALSE | TRUE | 5740500 | 1159447.375 | 4.951066 |
| IL13RA2 | 1 | 2 | 4.2 | 3 | 0.006201 | 1.5764 | 6915800 | 0 | 0 | 0 | 384210 | IL13RA2 | 2656 | 3068020.334 | 617371.484 | TRUE | TRUE | 3068020.334 | 617371.484 | 4.969488 |
| GATAD2B;G | 6 | 2 | 8 | 6 | 0 | 5.6357 | 6307000 | 5972800 | 6758500 | 0 | 1822100 | GATAD2B | 2011 | 6758500 | 1359402.112 | FALSE | TRUE | 6758500 | 1359402.112 | 4.971671 |
| MAP7 | 8 | 4 | 7.2 | 3 | 0 | 6.0057 | 0 | 0 | 16574000 | 0 | 863210 | MAP7 | 2372 | 16574000 | 3270788.489 | FALSE | TRUE | 16574000 | 3270788.489 | 5.06728 |
| CWF19L1 | 3 | 2 | 6.5 | 1 | 0.00046 | 3.992 | 0 | 0 | 6706500 | 0 | 467430 | CWF19L1 | 2606 | 6706500 | 1318466.555 | FALSE | TRUE | 6706500 | 1318466.555 | 5.086591 |
| GPD2 | 3 | 3 | 6.5 | 3 | 0 | 6.5555 | 0 | 0 | 9652500 | 0 | 650910 | GPD2 | 2490 | 9652500 | 1879996.516 | FALSE | TRUE | 9652500 | 1879996.516 | 5.134318 |
| ZC3H15 | 2 | 2 | 6.8 | 3 | 0.000803 | 2.7438 | 0 | 0 | 14715000 | 0 | 953720 | ZC3H15 | 2330 | 14715000 | 2832335.76 | FALSE | TRUE | 14715000 | 2832335.76 | 5.195359 |
| MTCH1 | 3 | 2 | 5.8 | 2 | 0.002575 | 2.0218 | 16041000 | 0 | 13234000 | 0 | 1768800 | MTCH1 | 2026 | 13234000 | 2519230.799 | FALSE | TRUE | 13234000 | 2519230.799 | 5.253191 |
| RNPS1 | 8 | 2 | 12.8 | 5 | 0 | 10.676 | 9991100 | 9354700 | 10815000 | 0 | 3427200 | RNPS1 | 1669 | 10815000 | 2050691.471 | FALSE | TRUE | 10815000 | 2050691.471 | 5.273831 |
| HINT2 | 1 | 3 | 28.2 | 5 | 0 | 19.198 | 0 | 35439000 | 0 | 0 | 5691600 | HINT2 | 1354 | 4186456.689 | 778789.045 | TRUE | TRUE | 4186456.689 | 778789.045 | 5.375598 |
| BAZ1B | 2 | 2 | 1.6 | 3 | 0.001136 | 2.2782 | 0 | 0 | 7871700 | 0 | 240540 | BAZ1B | 2768 | 7871700 | 1419241.735 | FALSE | TRUE | 7871700 | 1419241.735 | 5.546412 |
| DDAH2 | 5 | 2 | 9.8 | 3 | 0.000453 | 3.8099 | 9704300 | 7962000 | 12901000 | 0 | 2093800 | DDAH2 | 1940 | 12901000 | 2323767.549 | FALSE | TRUE | 12901000 | 2323767.549 | 5.55176 |
| COX6C | 1 | 2 | 20 | 4 | 0.001156 | 2.4046 | 0 | 69477000 | 0 | 0 | 34459000 | COX6C | 491 | 4911986.14 | 882334.5321 | TRUE | TRUE | 4911986.14 | 882334.5321 | 5.567034 |
| NQO2 | 4 | 3 | 19.7 | 3 | 0 | 6.9395 | 0 | 12397000 | 0 | 0 | 2572800 | NQO2 | 1814 | 6906889.634 | 1239933.003 | TRUE | TRUE | 6906889.634 | 1239933.003 | 5.570373 |
| LAD1 | 5 | 14 | 33.1 | 26 | 0 | 48.947 | 11090000 | 11149000 | 281110000 | 50419000 | 8528400 | LAD1 | 1137 | 281110000 | 50419000 | FALSE | FALSE | 281110000 | 50419000 | 5.575477 |
| SMARCC2 | 5 | 2 | 4.5 | 3 | 0.000464 | 4.0972 | 6568400 | 5310800 | 17386000 | 0 | 731190 | SMARCC2 | 2445 | 17386000 | 3109331.496 | FALSE | TRUE | 17386000 | 3109331.496 | 5.591556 |
| NDUFB1 | 2 | 2 | 34.5 | 3 | 0 | 5.6042 | 0 | 6804300 | 8852000 | 0 | 7775800 | NDUFB1 | 1186 | 8852000 | 1566134.378 | FALSE | TRUE | 8852000 | 1566134.378 | 5.652133 |
| HDGFRP2 | 7 | 2 | 15.7 | 3 | 0.00082 | 2.9105 | 0 | 10851000 | 0 | 0 | 3283800 | HDGFRP2 | 1695 | 9885235.147 | 1746313.326 | TRUE | TRUE | 9885235.147 | 1746313.326 | 5.660631 |
| WTAP | 1 | 2 | 5.8 | 2 | 0 | 6.4456 | 0 | 10759000 | 0 | 0 | 921590 | WTAP | 2340 | 8097597.693 | 1422117.471 | TRUE | TRUE | 8097597.693 | 1422117.471 | 5.694043 |
| AP2A2 | 14 | 2 | 12.6 | 2 | 0.000444 | 3.598 | 6286400 | 7607200 | 9625900 | 0 | 639450 | AP2A2 | 2496 | 9625900 | 1685268.691 | FALSE | TRUE | 9625900 | 1685268.691 | 5.711789 |
| LPCAT1 | 3 | 3 | 6.6 | 4 | 0 | 6.4188 | 14105000 | 18857000 | 8361400 | 0 | 2166200 | LPCAT1 | 1921 | 8361400 | 1455189.295 | FALSE | TRUE | 8361400 | 1455189.295 | 5.745919 |
| RAP1A | 2 | 2 | 42.9 | 2 | 0.00047 | 4.2344 | 0 | 5925900 | 0 | 0 | 1222600 | RAP1A | 2212 | 3767477.685 | 653295.8877 | TRUE | TRUE | 3767477.685 | 653295.8877 | 5.766878 |
| TRIM25 | 2 | 6 | 11.6 | 5 | 0 | 13.716 | 0 | 0 | 21290000 | 0 | 1784200 | TRIM25 | 2022 | 21290000 | 3683836.041 | FALSE | TRUE | 21290000 | 3683836.041 | 5.779302 |
| NEDD4;NED | 24 | 2 | 1.8 | 2 | 0.004919 | 1.7245 | 8501700 | 0 | 0 | 0 | 218250 | NEDD4 | 2782 | 5439671.056 | 939799.8009 | TRUE | TRUE | 5439671.056 | 939799.8009 | 5.788117 |
| DRAP1 | 5 | 3 | 13.7 | 5 | 0 | 5.0917 | 21433000 | 15898000 | 14586000 | 0 | 6089000 | DRAP1 | 1318 | 14586000 | 2429167.408 | FALSE | TRUE | 14586000 | 2429167.408 | 6.004526 |
| MRRF | 9 | 2 | 12.4 | 3 | 0.00046 | 3.9902 | 7875500 | 0 | 8731600 | 0 | 2021600 | MRRF | 1960 | 8731600 | 1444354.509 | FALSE | TRUE | 8731600 | 1444354.509 | 6.04533 |
| AHCTF1 | 3 | 3 | 1.5 | 4 | 0 | 4.8331 | 6547200 | 8194900 | 11529000 | 0 | 248000 | AHCTF1 | 2763 | 11529000 | 1906351.905 | FALSE | TRUE | 11529000 | 1906351.905 | 6.047677 |
| SH3GL1 | 6 | 4 | 12.5 | 5 | 0 | 8.8118 | 11824000 | 17417000 | 7689900 | 0 | 2265400 | SH3GL1 | 1903 | 7689900 | 1263151.883 | FALSE | TRUE | 7689900 | 1263151.883 | 6.087866 |
| NDUFA2 | 2 | 2 | 25 | 2 | 0.003647 | 1.9461 | 0 | 0 | 7097700 | 0 | 7002900 | NDUFA2 | 1251 | 7097700 | 1164857.245 | FALSE | TRUE | 7097700 | 1164857.245 | 6.093193 |
| SAMM50 | 1 | 3 | 9 | 9 | 0 | 11.334 | 13410000 | 9124900 | 12976000 | 0 | 1228400 | SAMM50 | 2211 | 12976000 | 2114188.046 | FALSE | TRUE | 12976000 | 2114188.046 | 6.137581 |
| HTATSF1 | 3 | 4 | 5.7 | 6 | 0 | 9.1407 | 27204000 | 24820000 | 14755000 | 0 | 1441700 | HTATSF1 | 2138 | 14755000 | 2395355.199 | FALSE | TRUE | 14755000 | 2395355.199 | 6.159838 |
| CFAP20 | 1 | 2 | 10.9 | 4 | 0.000457 | 3.8951 | 0 | 0 | 10775000 | 0 | 1697400 | CFAP20 | 2049 | 10775000 | 1743304.195 | FALSE | TRUE | 10775000 | 1743304.195 | 6.180792 |
| NUP98 | 9 | 3 | 1.8 | 4 | 0 | 4.7495 | 0 | 0 | 6094900 | 0 | 137350 | NUP98 | 2859 | 6094900 | 982884.6765 | FALSE | TRUE | 6094900 | 982884.6765 | 6.201033 |
| TRNT1 | 11 | 2 | 6 | 4 | 0 | 5.2244 | 8996000 | 8897700 | 12005000 | 0 | 930130 | TRNT1 | 2337 | 12005000 | 1933688.507 | FALSE | TRUE | 12005000 | 1933688.507 | 6.208342 |
| COA7 | 1 | 2 | 10 | 3 | 0 | 4.487 | 0 | 0 | 13293000 | 0 | 2415700 | COA7 | 1854 | 13293000 | 2109509.841 | FALSE | TRUE | 13293000 | 2109509.841 | 6.301464 |
| FDX1 | 1 | 2 | 9.2 | 1 | 0.004631 | 1.8044 | 0 | 12697000 | 0 | 0 | 1785400 | FDX1 | 2020 | 5343151.404 | 845434.9043 | TRUE | TRUE | 5343151.404 | 845434.9043 | 6.320003 |
| OSTF1 | 1 | 3 | 24.8 | 6 | 0 | 14.631 | 23498000 | 16766000 | 16175000 | 0 | 4575100 | OSTF1 | 1494.5 | 16175000 | 2525306.074 | FALSE | TRUE | 16175000 | 2525306.074 | 6.405164 |
| STXBP3 | 1 | 2 | 2.7 | 4 | 0.00113 | 2.2385 | 8902900 | 7661700 | 6926300 | 0 | 838340 | STXBP3 | 2380 | 6926300 | 1041564.239 | FALSE | TRUE | 6926300 | 1041564.239 | 6.649902 |
| MRPL44 | 1 | 2 | 8.7 | 3 | 0 | 11.729 | 0 | 0 | 12143000 | 0 | 1492800 | MRPL44 | 2120 | 12143000 | 1779855.479 | FALSE | TRUE | 12143000 | 1779855.479 | 6.822464 |
| TATDN1 | 11 | 4 | 14.3 | 5 | 0 | 5.1813 | 0 | 16900000 | 0 | 0 | 2327700 | TATDN1 | 1881 | 8023832.376 | 1170691.652 | TRUE | TRUE | 8023832.376 | 1170691.652 | 6.853925 |
| NUFIP2 | 1 | 4 | 7.1 | 6 | 0 | 7.056 | 11376000 | 0 | 0 | 0 | 1001800 | NUFIP2 | 2310 | 8622840.605 | 1235694.162 | TRUE | TRUE | 8622840.605 | 1235694.162 | 6.978135 |
| NAA50 | 10 | 5 | 44.2 | 5 | 0 | 9.1374 | 20375000 | 24293000 | 24701000 | 0 | 8572200 | NAA50 | 1132 | 24701000 | 3511896.572 | FALSE | TRUE | 24701000 | 3511896.572 | 7.033521 |
| SCARB1 | 7 | 2 | 5.2 | 2 | 0.000421 | 3.1208 | 0 | 0 | 14877000 | 0 | 835750 | SCARB1 | 2382 | 14877000 | 2071576.165 | FALSE | TRUE | 14877000 | 2071576.165 | 7.181488 |
| AP3M1 | 4 | 4 | 14.1 | 6 | 0 | 5.3114 | 13045000 | 11713000 | 16498000 | 0 | 1984700 | AP3M1 | 1971 | 16498000 | 2230153.567 | FALSE | TRUE | 16498000 | 2230153.567 | 7.397697 |
| AMPD2 | 8 | 2 | 18.4 | 2 | 0.003655 | 1.9702 | 0 | 0 | 6966800 | 0 | 3699800 | AMPD2 | 1611 | 6966800 | 935411.7451 | FALSE | TRUE | 6966800 | 935411.7451 | 7.447843 |
| PAPSS2 | 2 | 3 | 5.7 | 2 | 0 | 5.692 | 0 | 0 | 13850000 | 0 | 627750 | PAPSS2 | 2500 | 13850000 | 1843409.852 | FALSE | TRUE | 13850000 | 1843409.852 | 7.513251 |
| NOL9 | 1 | 4 | 7 | 5 | 0 | 8.186 | 12751000 | 14913000 | 11308000 | 0 | 1559900 | NOL9 | 2098 | 11308000 | 1483435.763 | FALSE | TRUE | 11308000 | 1483435.763 | 7.622844 |
| S100A8 | 1 | 3 | 31.2 | 5 | 0 | 7.3706 | 61224000 | 0 | 0 | 0 | 7818500 | S100A8 | 1181 | 7397907.569 | 970422.1353 | TRUE | TRUE | 7397907.569 | 970422.1353 | 7.623391 |
| TSPAN6 | 3 | 2 | 8.6 | 3 | 0.000443 | 3.5978 | 0 | 11582000 | 10402000 | 0 | 3415900 | TSPAN6 | 1673 | 10402000 | 1358051.235 | FALSE | TRUE | 10402000 | 1358051.235 | 7.659505 |
| MRPS21 | 1 | 2 | 29.9 | 6 | 0 | 15.918 | 6786600 | 11683000 | 0 | 0 | 5488900 | MRPS21 | 1371 | 6544605.664 | 846526.7626 | TRUE | TRUE | 6544605.664 | 846526.7626 | 7.731127 |
| ADAM10 | 2 | 2 | 3.9 | 3 | 0 | 4.6737 | 0 | 0 | 11282000 | 0 | 371840 | ADAM10 | 2669 | 11282000 | 1429910.14 | FALSE | TRUE | 11282000 | 1429910.14 | 7.890006 |
| CSTF2;CST | 5 | 3 | 9.6 | 2 | 0 | 6.5931 | 0 | 0 | 13559000 | 0 | 909080 | CSTF2 | 2347 | 13559000 | 1686867.976 | FALSE | TRUE | 13559000 | 1686867.976 | 8.037973 |
| EXOSC5 | 3 | 3 | 23.4 | 3 | 0 | 11.161 | 0 | 16843000 | 0 | 0 | 3022500 | EXOSC5 | 1747 | 9334489.731 | 1154804.558 | TRUE | TRUE | 9334489.731 | 1154804.558 | 8.083177 |
| ADNP | 3 | 2 | 2.5 | 2 | 0 | 5.8616 | 0 | 0 | 7630500 | 0 | 136960 | ADNP | 2861 | 7630500 | 943654.7818 | FALSE | TRUE | 7630500 | 943654.7818 | 8.086114 |
| NF1 | 6 | 2 | 1.6 | 1 | 0 | 5.151 | 0 | 0 | 9652700 | 0 | 119340 | NF1 | 2873 | 9652700 | 1177618.11 | FALSE | TRUE | 9652700 | 1177618.11 | 8.1968 |
| F11R | 2 | 2 | 6.4 | 3 | 0.000427 | 3.268 | 0 | 0 | 30818000 | 0 | 2327200 | F11R | 1882.5 | 30818000 | 3651948.858 | FALSE | TRUE | 30818000 | 3651948.858 | 8.438782 |
| PELP1 | 7 | 4 | 3.2 | 6 | 0.000428 | 3.2814 | 12398000 | 15344000 | 20299000 | 0 | 1239000 | PELP1 | 2207 | 20299000 | 2263131.726 | FALSE | TRUE | 20299000 | 2263131.726 | 8.969429 |
| PRPF4 | 2 | 3 | 7.9 | 6 | 0 | 7.6584 | 14281000 | 14093000 | 15055000 | 0 | 1489700 | PRPF4 | 2121 | 15055000 | 1655156.776 | FALSE | TRUE | 15055000 | 1655156.776 | 9.095815 |
| MRPS36 | 1 | 2 | 30.1 | 5 | 0 | 6.2755 | 8834000 | 7982600 | 8687200 | 0 | 5505900 | MRPS36 | 1368 | 8687200 | 928320.05 | FALSE | TRUE | 8687200 | 928320.05 | 9.35798 |
| SQLE | 3 | 2 | 6.5 | 1 | 0.000456 | 3.8895 | 0 | 0 | 19754000 | 0 | 1473700 | SQLE | 2125 | 19754000 | 2031653.425 | FALSE | TRUE | 19754000 | 2031653.425 | 9.723115 |
| POLDIP2 | 2 | 2 | 6.9 | 4 | 0 | 5.1234 | 0 | 0 | 14629000 | 0 | 1573800 | POLDIP2 | 2090 | 14629000 | 1490486.155 | FALSE | TRUE | 14629000 | 1490486.155 | 9.814918 |
| AK4 | 2 | 3 | 17.5 | 5 | 0 | 5.0297 | 7857100 | 8474900 | 17012000 | 0 | 2399300 | AK4 | 1860 | 17012000 | 1660978.715 | FALSE | TRUE | 17012000 | 1660978.715 | 10.24215 |
| CDK5RAP3 | 10 | 2 | 5.7 | 5 | 0.002228 | 2.1246 | 6812200 | 5506500 | 8149000 | 0 | 1386400 | CDK5RAP3 | 2164 | 8149000 | 775038.4701 | FALSE | TRUE | 8149000 | 775038.4701 | 10.51432 |
| NDC80 | 1 | 2 | 3.7 | 2 | 0.006218 | 1.5967 | 0 | 0 | 26238000 | 0 | 610560 | NDC80 | 2512 | 26238000 | 2486942.374 | FALSE | TRUE | 26238000 | 2486942.374 | 10.5503 |
| SNRPA | 7 | 3 | 16 | 9 | 0 | 15.993 | 58336000 | 50152000 | 39241000 | 0 | 12998000 | SNRPA | 921 | 39241000 | 3489094.83 | FALSE | TRUE | 39241000 | 3489094.83 | 11.24676 |
| TBCB | 8 | 6 | 22.3 | 12 | 0 | 14.879 | 72795000 | 69144000 | 35099000 | 0 | 9867400 | TBCB | 1053 | 35099000 | 3039249.314 | FALSE | TRUE | 35099000 | 3039249.314 | 11.54858 |
| DNMT1 | 7 | 3 | 2.5 | 2 | 0 | 5.2665 | 9893800 | 10896000 | 10070000 | 0 | 397140 | DNMT1 | 2644 | 10070000 | 859417.5798 | FALSE | TRUE | 10070000 | 859417.5798 | 11.71724 |
| DDX47 | 3 | 6 | 18 | 5 | 0 | 8.9 | 0 | 0 | 24544000 | 0 | 2777000 | DDX47 | 1788 | 24544000 | 2049919.868 | FALSE | TRUE | 24544000 | 2049919.868 | 11.97315 |
| NUBP2 | 7 | 2 | 8.4 | 2 | 0.001146 | 2.3186 | 0 | 0 | 14049000 | 0 | 1445400 | NUBP2 | 2136 | 14049000 | 1158341.615 | FALSE | TRUE | 14049000 | 1158341.615 | 12.12855 |
| IPO9 | 1 | 5 | 5.9 | 3 | 0 | 7.3746 | 37282000 | 45228000 | 36177000 | 0 | 3215400 | IPO9 | 1711 | 36177000 | 2950956.87 | FALSE | TRUE | 36177000 | 2950956.87 | 12.25941 |
| CECR5 | 2 | 3 | 12.2 | 4 | 0 | 20.885 | 8551200 | 0 | 20595000 | 0 | 2269600 | CECR5 | 1902 | 20595000 | 1678861.423 | FALSE | TRUE | 20595000 | 1678861.423 | 12.26724 |
| ALDOC | 7 | 4 | 28 | 19 | 0 | 40.681 | 35899000 | 33817000 | 40961000 | 0 | 5248000 | ALDOC | 1397 | 40961000 | 3336062.39 | FALSE | TRUE | 40961000 | 3336062.39 | 12.27825 |
| NDRG1 | 14 | 9 | 37.8 | 23 | 0 | 38.27 | 211280000 | 72006000 | 22301000 | 0 | 25535000 | NDRG1 | 604 | 22301000 | 1809946.33 | FALSE | TRUE | 22301000 | 1809946.33 | 12.32136 |
| IGF2BP2 | 7 | 5 | 12.7 | 6 | 0 | 25.148 | 0 | 20794000 | 41217000 | 0 | 2396400 | IGF2BP2 | 1862 | 41217000 | 3289105.831 | FALSE | TRUE | 41217000 | 3289105.831 | 12.53137 |
| GRN | 8 | 2 | 4.9 | 5 | 0 | 5.9789 | 0 | 0 | 21469000 | 0 | 1064600 | GRN | 2282 | 21469000 | 1673797.171 | FALSE | TRUE | 21469000 | 1673797.171 | 12.82652 |
| TESC | 2 | 2 | 13.4 | 3 | 0.00042 | 3.1117 | 0 | 0 | 19447000 | 0 | 2219500 | TESC | 1913 | 19447000 | 1504908.054 | FALSE | TRUE | 19447000 | 1504908.054 | 12.92238 |
| EIF2B1 | 4 | 2 | 11.2 | 5 | 0.004965 | 1.7629 | 8964700 | 14170000 | 15533000 | 0 | 4859500 | EIF2B1 | 1442 | 15533000 | 1195457.892 | FALSE | TRUE | 15533000 | 1195457.892 | 12.99335 |
| ATP5J2-PT | 7 | 2 | 44.4 | 6 | 0 | 6.8325 | 18894000 | 15899000 | 17626000 | 0 | 13256000 | ATP5J2-PT | 905 | 17626000 | 1353918.956 | FALSE | TRUE | 17626000 | 1353918.956 | 13.0185 |
| PPIF | 4 | 2 | 21.3 | 5 | 0 | 10.981 | 28500000 | 20337000 | 23038000 | 0 | 7126000 | PPIF | 1243 | 23038000 | 1764837.332 | FALSE | TRUE | 23038000 | 1764837.332 | 13.05389 |
| FKBP9 | 5 | 6 | 11.4 | 6 | 0 | 6.2266 | 25338000 | 24794000 | 28292000 | 0 | 3373000 | FKBP9 | 1678.5 | 28292000 | 2117360.653 | FALSE | TRUE | 28292000 | 2117360.653 | 13.36192 |
| TMEM43 | 2 | 8 | 27 | 15 | 0 | 39.377 | 1.07E+08 | 106380000 | 38509000 | 0 | 11088000 | TMEM43 | 993 | 38509000 | 2851110.158 | FALSE | TRUE | 38509000 | 2851110.158 | 13.50667 |
| SLC25A22; | 15 | 2 | 8.9 | 6 | 0 | 6.1505 | 0 | 7792400 | 14287000 | 0 | 1419000 | SLC25A22 | 2149 | 14287000 | 1001730.388 | FALSE | TRUE | 14287000 | 1001730.388 | 14.26232 |
| YY2;YY1;Z | 4 | 2 | 4.6 | 2 | 0.000811 | 2.8238 | 0 | 0 | 16625000 | 0 | 2412800 | YY2 | 1857 | 16625000 | 1079470.59 | FALSE | TRUE | 16625000 | 1079470.59 | 15.40107 |
| SEC22B | 1 | 4 | 21.9 | 17 | 0 | 11.329 | 24449000 | 17372000 | 14743000 | 0 | 4291900 | SEC22B | 1532 | 14743000 | 939611.7922 | FALSE | TRUE | 14743000 | 939611.7922 | 15.69052 |
| ATP6V0D1 | 4 | 2 | 10.8 | 5 | 0 | 6.5098 | 16140000 | 10604000 | 26634000 | 0 | 4084700 | ATP6V0D1 | 1553 | 26634000 | 1673883.889 | FALSE | TRUE | 26634000 | 1673883.889 | 15.9115 |
| MACROD1 | 1 | 3 | 10.8 | 4 | 0 | 6.0614 | 0 | 0 | 40880000 | 0 | 3177200 | MACROD1 | 1718 | 40880000 | 2370110.284 | FALSE | TRUE | 40880000 | 2370110.284 | 17.24814 |
| GPX1 | 3 | 2 | 17.8 | 3 | 0.000798 | 2.7055 | 0 | 0 | 16497000 | 0 | 2864200 | GPX1 | 1770 | 16497000 | 927601.4786 | FALSE | TRUE | 16497000 | 927601.4786 | 17.78458 |
| CCDC58 | 3 | 3 | 27.7 | 5 | 0 | 9.8394 | 0 | 0 | 19818000 | 0 | 10481000 | CCDC58 | 1022 | 19818000 | 1085945.1 | FALSE | TRUE | 19818000 | 1085945.1 | 18.24954 |
| LYPLA2 | 5 | 3 | 20.7 | 8 | 0.000442 | 3.566 | 19317000 | 16187000 | 22330000 | 0 | 6957000 | LYPLA2 | 1254 | 22330000 | 1128499.128 | FALSE | TRUE | 22330000 | 1128499.128 | 19.78734 |
| USMG5 | 1 | 2 | 43.1 | 15 | 0 | 31.434 | 37819000 | 32874000 | 34569000 | 0 | 36916000 | USMG5 | 470 | 34569000 | 1741667.842 | FALSE | TRUE | 34569000 | 1741667.842 | 19.84822 |
| SLK | 2 | 6 | 5.4 | 9 | 0 | 9.332 | 15902000 | 0 | 31039000 | 0 | 1314700 | SLK | 2177 | 31039000 | 1557560.688 | FALSE | TRUE | 31039000 | 1557560.688 | 19.92796 |
| PSIP1 | 3 | 7 | 15.5 | 13 | 0 | 18.33 | 44835000 | 64043000 | 28491000 | 0 | 5256300 | PSIP1 | 1394 | 28491000 | 1332309.58 | FALSE | TRUE | 28491000 | 1332309.58 | 21.38467 |
| EPB41L2 | 19 | 6 | 8.2 | 7 | 0 | 13.044 | 31052000 | 33741000 | 28508000 | 0 | 1854100 | EPB41L2 | 2004 | 28508000 | 1257544.149 | FALSE | TRUE | 28508000 | 1257544.149 | 22.66958 |
| LIG1 | 9 | 4 | 5.8 | 6 | 0 | 6.2163 | 14697000 | 15995000 | 36411000 | 0 | 1595600 | LIG1 | 2082 | 36411000 | 1589379.534 | FALSE | TRUE | 36411000 | 1589379.534 | 22.90894 |
| CTSL | 6 | 8 | 31.2 | 31 | 0 | 49.923 | 252250000 | 539970000 | 50267000 | 0 | 78451000 | CTSL | 277 | 50267000 | 1871336.354 | FALSE | TRUE | 50267000 | 1871336.354 | 26.86155 |
| IST1 | 12 | 3 | 12.1 | 2 | 0 | 4.4263 | 0 | 11432000 | 12597000 | 0 | 3830400 | IST1 | 1596 | 12597000 | 460352.9759 | FALSE | TRUE | 12597000 | 460352.9759 | 27.36379 |
| HMGCS2 | 3 | 2 | 5.8 | 4 | 0.001494 | 2.1823 | 0 | 0 | 18004000 | 0 | 832650 | HMGCS2 | 2383 | 18004000 | 634840.2594 | FALSE | TRUE | 18004000 | 634840.2594 | 28.35989 |
| NDUFS8 | 6 | 3 | 20.7 | 5 | 0 | 10.402 | 42866000 | 38067000 | 81371000 | 0 | 13283000 | NDUFS8 | 904 | 81371000 | 1400500.429 | FALSE | TRUE | 81371000 | 1400500.429 | 58.10137 |
| TLN2 | 4 | 2 | 3.8 | 7 | 0.004956 | 1.7491 | 123760000 | 123130000 | 130170000 | 0 | 4248300 | TLN2 | 1540 | 130170000 | 1466775.171 | FALSE | TRUE | 130170000 | 1466775.171 | 88.74571 |
| GOLGB1 | 6 | 6 | 2 | 6 | 0 | 6.5436 | 0 | 0 | 288830000 | 0 | 2260800 | GOLGB1 | 1905 | 288830000 | 2406020.544 | FALSE | TRUE | 288830000 | 2406020.544 | 120.0447 |

Supplementary Table S2: The proteins interacted with TRIM31 were identified by CO-IP and mass spectrometry (MS)

| Gene name | Peptides | Unique pe | Peptides | Peptides V | Unique pe | Unique pe | Sequence c | Mol. weig | Sequence | Q-value | Score | Sequence | Sequence | intensity 31 | intensity VECTOR | FC (31/vector) |
|---|---|---|---|---|---|---|---|---|---|---|---|---|---|---|---|---|
| TRIM31 | 29 | 29 | 29 | 0 | 29 | 0 | 69.9 | 48.243 | 425 | 0 | 323.31 | 69.9 | 0 | 778710000 | 0 | #DIV/0! |
| RPS27A | 3 | 1 | 3 | 0 | 1 | 0 | 24.4 | 17.965 | 156 | 0 | 63.115 | 24.4 | 0 | 8261100 | 0 | #DIV/0! |
| WDR87 | 1 | 1 | 1 | 0 | 1 | 0 | 0.4 | 337.83 | 2912 | 0.0023866 | 6.4195 | 0.4 | 0 | 7774400 | 0 | #DIV/0! |
| RPS10 | 2 | 2 | 2 | 1 | 2 | 1 | 19.8 | 14.294 | 121 | 0 | 12.772 | 19.8 | 7.4 | 3495700 | 0 | #DIV/0! |
| LMNB1 | 6 | 6 | 5 | 2 | 5 | 2 | 15.4 | 66.408 | 586 | 0 | 56.906 | 13.5 | 7 | 3471800 | 0 | #DIV/0! |
| COL1A1 | 2 | 2 | 1 | 1 | 1 | 1 | 1.6 | 138.94 | 1464 | 0 | 11.253 | 0.6 | 1 | 3194000 | 0 | #DIV/0! |
| TP53 | 2 | 2 | 2 | 0 | 2 | 0 | 11.2 | 43.66 | 393 | 0.0023126 | 6.2582 | 11.2 | 0 | 3023211 | 0 | #DIV/0! |
| PHB2 | 2 | 2 | 2 | 0 | 2 | 0 | 12.2 | 23.615 | 213 | 0 | 12.877 | 12.2 | 0 | 2893200 | 0 | #DIV/0! |
| PHB | 3 | 3 | 3 | 0 | 3 | 0 | 16.4 | 22.27 | 201 | 0 | 22.181 | 16.4 | 0 | 2749000 | 0 | #DIV/0! |
| PCMT1 | 2 | 2 | 2 | 1 | 2 | 1 | 10 | 26.626 | 250 | 0 | 13.05 | 10 | 4.4 | 2702100 | 0 | #DIV/0! |
| TARDBP | 3 | 3 | 3 | 1 | 3 | 1 | 16 | 26.743 | 243 | 0 | 36.452 | 16 | 7.4 | 2160800 | 0 | #DIV/0! |
| NUFIP2 | 2 | 2 | 2 | 1 | 2 | 1 | 4.9 | 76.12 | 695 | 0 | 21.526 | 4.9 | 1.9 | 1976800 | 0 | #DIV/0! |
| RPS12 | 2 | 2 | 2 | 0 | 2 | 0 | 25 | 14.515 | 132 | 0 | 13.893 | 25 | 0 | 1948500 | 0 | #DIV/0! |
| SNRPN | 2 | 2 | 2 | 1 | 2 | 1 | 8.9 | 17.546 | 169 | 0 | 13.044 | 8.9 | 4.7 | 1729800 | 0 | #DIV/0! |
| CPSF7 | 3 | 3 | 2 | 2 | 2 | 2 | 14.6 | 24.387 | 219 | 0 | 18.11 | 11 | 9.6 | 1666500 | 0 | #DIV/0! |
| SNRNP200 | 2 | 2 | 2 | 1 | 2 | 1 | 1 | 244.5 | 2136 | 0 | 11.801 | 1 | 0.7 | 1623900 | 0 | #DIV/0! |
| RPN1 | 2 | 2 | 2 | 0 | 2 | 0 | 4.6 | 49.921 | 435 | 0 | 12.23 | 4.6 | 0 | 1531800 | 0 | #DIV/0! |
| HLA-B | 2 | 2 | 2 | 1 | 2 | 1 | 10.3 | 25.744 | 224 | 0 | 20.621 | 10.3 | 4 | 1397800 | 0 | #DIV/0! |
| TMA16 | 2 | 2 | 2 | 1 | 2 | 1 | 16 | 17.155 | 144 | 0 | 11.413 | 16 | 5.6 | 1377500 | 0 | #DIV/0! |
| MYBBP1A | 4 | 4 | 4 | 1 | 4 | 1 | 5.2 | 140.13 | 1252 | 0 | 33.709 | 5.2 | 0.9 | 1348500 | 0 | #DIV/0! |
| EPRS | 2 | 2 | 2 | 0 | 2 | 0 | 2.4 | 170.59 | 1512 | 0 | 13.179 | 2.4 | 0 | 1345100 | 0 | #DIV/0! |
| RAB6B | 1 | 1 | 1 | 1 | 1 | 1 | 22.4 | 5.8575 | 49 | 0.0024631 | 6.5802 | 22.4 | 22.4 | 1320800 | 0 | #DIV/0! |
| MRPS34 | 3 | 3 | 3 | 1 | 3 | 1 | 18.7 | 26.332 | 225 | 0 | 17.48 | 18.7 | 3.6 | 1304600 | 0 | #DIV/0! |
| U2SURP | 2 | 2 | 2 | 0 | 2 | 0 | 2.5 | 118.29 | 1029 | 0 | 11.767 | 2.5 | 0 | 1257400 | 0 | #DIV/0! |
| ATP5O | 2 | 2 | 2 | 1 | 2 | 1 | 10.3 | 23.277 | 213 | 0 | 13.215 | 10.3 | 4.7 | 1249200 | 0 | #DIV/0! |
| USO1 | 2 | 2 | 2 | 1 | 2 | 1 | 1.7 | 107.89 | 962 | 0 | 53.829 | 1.7 | 1.6 | 1227100 | 0 | #DIV/0! |
| EIF5B | 2 | 2 | 2 | 1 | 2 | 1 | 3.3 | 138.68 | 1220 | 0 | 17.112 | 3.3 | 1.7 | 1213300 | 0 | #DIV/0! |
| CPSF6 | 3 | 3 | 2 | 2 | 2 | 2 | 11.5 | 52.269 | 478 | 0 | 35.04 | 8.2 | 6.3 | 1153700 | 0 | #DIV/0! |
| NBPF19 | 1 | 1 | 1 | 0 | 1 | 0 | 7.2 | 26.705 | 235 | 0 | 7.283 | 7.2 | 0 | 1078600 | 0 | #DIV/0! |
| PCNA | 3 | 3 | 2 | 1 | 2 | 1 | 15.3 | 28.768 | 261 | 0 | 17.913 | 12.3 | 3.1 | 1006700 | 0 | #DIV/0! |
| VDAC1 | 1 | 1 | 1 | 0 | 1 | 0 | 6 | 20.274 | 183 | 0.0024691 | 6.5978 | 6 | 0 | 983570 | 0 | #DIV/0! |
| HOXA10 | 1 | 1 | 1 | 0 | 1 | 0 | 3.2 | 42.414 | 410 | 0 | 7.1316 | 3.2 | 0 | 952240 | 0 | #DIV/0! |
| HGF | 1 | 1 | 1 | 0 | 1 | 0 | 3.9 | 23.584 | 205 | 0.002439 | 6.5551 | 3.9 | 0 | 940820 | 0 | #DIV/0! |
| MCM3 | 3 | 3 | 2 | 2 | 2 | 2 | 5 | 91.929 | 818 | 0 | 16.875 | 3.4 | 4 | 926290 | 0 | #DIV/0! |
| ATP1A1 | 2 | 2 | 2 | 0 | 2 | 0 | 2.7 | 112.89 | 1023 | 0 | 14.24 | 2.7 | 0 | 908650 | 0 | #DIV/0! |
| SMN1 | 1 | 1 | 1 | 0 | 1 | 0 | 3.5 | 30.415 | 282 | 0 | 7.9403 | 3.5 | 0 | 853110 | 0 | #DIV/0! |
| PGRMC1 | 1 | 1 | 1 | 0 | 1 | 0 | 7.2 | 21.671 | 195 | 0 | 8.9196 | 7.2 | 0 | 830280 | 0 | #DIV/0! |
| SLC16A1 | 1 | 1 | 1 | 0 | 1 | 0 | 4.1 | 31.689 | 296 | 0.0046838 | 6.1963 | 4.1 | 0 | 806580 | 0 | #DIV/0! |
| BANF1 | 1 | 1 | 1 | 0 | 1 | 0 | 27 | 10.058 | 89 | 0 | 7.0826 | 27 | 0 | 782090 | 0 | #DIV/0! |
| CCT4 | 2 | 2 | 2 | 0 | 2 | 0 | 7.8 | 57.924 | 539 | 0 | 13.988 | 7.8 | 0 | 756570 | 0 | #DIV/0! |
| EEF1G | 2 | 2 | 2 | 0 | 2 | 0 | 4.6 | 50.118 | 437 | 0 | 10.836 | 4.6 | 0 | 729470 | 0 | #DIV/0! |
| HSPA5 | 3 | 2 | 3 | 1 | 2 | 0 | 4.9 | 72.332 | 654 | 0 | 22.782 | 4.9 | 2.4 | 662010 | 0 | #DIV/0! |
| FUBP1 | 4 | 1 | 4 | 2 | 1 | 0 | 5.3 | 68.903 | 655 | 0.0025253 | 6.8228 | 5.3 | 2.1 | 613930 | 0 | #DIV/0! |
| ATP5B | 2 | 2 | 2 | 0 | 2 | 0 | 7.2 | 38.138 | 362 | 0 | 11.254 | 7.2 | 0 | 605320 | 0 | #DIV/0! |
| BSG | 1 | 1 | 1 | 0 | 1 | 0 | 9.5 | 20.542 | 189 | 0.0046404 | 6.1646 | 9.5 | 0 | 594850 | 0 | #DIV/0! |
| NDUFA4 | 1 | 1 | 1 | 0 | 1 | 0 | 14.8 | 9.3697 | 81 | 0.0025641 | 6.9554 | 14.8 | 0 | 571840 | 0 | #DIV/0! |
| PTRH2 | 1 | 1 | 1 | 0 | 1 | 0 | 8.3 | 19.325 | 180 | 0.0045872 | 6.0827 | 8.3 | 0 | 560900 | 0 | #DIV/0! |
| SS18L1 | 1 | 1 | 1 | 0 | 1 | 0 | 5.7 | 23.41 | 211 | 0.004662 | 6.1812 | 5.7 | 0 | 557370 | 0 | #DIV/0! |
| RPS21 | 1 | 1 | 1 | 0 | 1 | 0 | 12.3 | 8.85 | 81 | 0.0068337 | 6.0451 | 12.3 | 0 | 509200 | 0 | #DIV/0! |
| MSI2 | 1 | 1 | 1 | 0 | 1 | 0 | 3.7 | 28.776 | 267 | 0.0024752 | 6.6291 | 3.7 | 0 | 491670 | 0 | #DIV/0! |
| PHF6 | 1 | 1 | 1 | 1 | 1 | 1 | 4.3 | 36.43 | 324 | 0.004717 | 6.2433 | 4.3 | 4.3 | 469050 | 0 | #DIV/0! |
| NOB1 | 2 | 2 | 2 | 1 | 2 | 1 | 6.3 | 46.674 | 412 | 0 | 11.987 | 6.3 | 2.2 | 440870 | 0 | #DIV/0! |
| FTSJ3 | 1 | 1 | 1 | 0 | 1 | 0 | 2.6 | 96.557 | 847 | 0.0024814 | 6.6506 | 2.6 | 0 | 412600 | 0 | #DIV/0! |
| RBBP7 | 1 | 1 | 1 | 0 | 1 | 0 | 5.6 | 31.503 | 285 | 0.0045662 | 6.0732 | 5.6 | 0 | 397270 | 0 | #DIV/0! |
| TSR1 | 1 | 1 | 1 | 0 | 1 | 0 | 1.4 | 91.809 | 804 | 0.0046083 | 6.1241 | 1.4 | 0 | 362690 | 0 | #DIV/0! |
| SSR4 | 1 | 1 | 1 | 0 | 1 | 0 | 12.8 | 16.245 | 148 | 0.0024213 | 6.5054 | 12.8 | 0 | 338590 | 0 | #DIV/0! |
| ATP5J2-PT | 1 | 1 | 1 | 0 | 1 | 0 | 20.4 | 5.9149 | 54 | 0.0045767 | 6.0811 | 20.4 | 0 | 316150 | 0 | #DIV/0! |
| RBM14 | 2 | 2 | 2 | 0 | 2 | 0 | 3 | 69.491 | 669 | 0 | 14.649 | 3 | 0 | 306100 | 0 | #DIV/0! |
| ASPH | 1 | 1 | 1 | 0 | 1 | 0 | 2.6 | 85.862 | 758 | 0.0024038 | 6.4599 | 2.6 | 0 | 287370 | 0 | #DIV/0! |
| SS18 | 1 | 1 | 1 | 0 | 1 | 0 | 3.5 | 43.519 | 395 | 0 | 16.632 | 3.5 | 0 | 285870 | 0 | #DIV/0! |
| DICER1 | 1 | 1 | 1 | 0 | 1 | 0 | 0.5 | 218.68 | 1922 | 0.0025773 | 6.9632 | 0.5 | 0 | 279000 | 0 | #DIV/0! |
| HSP90AB2P | 4 | 1 | 4 | 2 | 1 | 0 | 11.8 | 44.348 | 381 | 0 | 7.5868 | 11.8 | 5.5 | 278990 | 0 | #DIV/0! |
| FAM199X | 1 | 1 | 1 | 0 | 1 | 0 | 3.9 | 42.801 | 388 | 0.0047506 | 6.2953 | 3.9 | 0 | 236760 | 0 | #DIV/0! |
| PRPS1 | 3 | 3 | 2 | 1 | 2 | 1 | 11.6 | 31.392 | 285 | 0 | 17.63 | 7.4 | 4.2 | 213950 | 0 | #DIV/0! |
| CAD | 2 | 2 | 2 | 0 | 2 | 0 | 1.2 | 236.02 | 2162 | 0 | 10.928 | 1.2 | 0 | 207650 | 0 | #DIV/0! |
| RPN2 | 1 | 1 | 1 | 0 | 1 | 0 | 7.4 | 17.808 | 162 | 0 | 16.086 | 7.4 | 0 | 168460 | 0 | #DIV/0! |
| RRP12 | 1 | 1 | 1 | 0 | 1 | 0 | 1.8 | 143.7 | 1297 | 0 | 7.7261 | 1.8 | 0 | 146250 | 0 | #DIV/0! |
| CNP | 1 | 1 | 1 | 0 | 1 | 0 | 19.2 | 13.647 | 120 | 0 | 7.2111 | 19.2 | 0 | 103200 | 0 | #DIV/0! |
| EIF2AK2 | 1 | 1 | 1 | 0 | 1 | 0 | 2.2 | 62.094 | 551 | 0 | 11.539 | 2.2 | 0 | 74065 | 0 | #DIV/0! |
| KHDRBS1 | 14 | 12 | 13 | 13 | 11 | 11 | 30.9 | 48.227 | 443 | 0 | 210.11 | 30.9 | 30.9 | 1227800000 | 1292400000 | 0.950015 |
| HNRNPF | 17 | 15 | 17 | 13 | 15 | 11 | 65.8 | 45.671 | 415 | 0 | 323.31 | 65.8 | 58.1 | 933100000 | 643490000 | 1.450061 |
| ALB | 6 | 6 | 5 | 6 | 5 | 6 | 8.6 | 69.226 | 604 | 0 | 53.313 | 7.1 | 8.6 | 569670000 | 734380000 | 0.775716 |
| TUBB4B | 23 | 1 | 23 | 21 | 1 | 1 | 62 | 49.83 | 445 | 0 | 323.31 | 62 | 59.8 | 540150000 | 372090000 | 1.451665 |
| HNRNPH1 | 15 | 10 | 15 | 14 | 10 | 9 | 47.9 | 51.229 | 472 | 0 | 310.42 | 47.9 | 47.9 | 361740000 | 463810000 | 0.779931 |
| TRIM21 | 24 | 24 | 23 | 21 | 23 | 21 | 54.5 | 54.169 | 475 | 0 | 323.31 | 52.6 | 49.3 | 341180000 | 202830000 | 1.682098 |
| TUBA1B | 20 | 1 | 18 | 16 | 1 | 1 | 54.8 | 50.151 | 451 | 0 | 279.99 | 51.4 | 46.3 | 226460000 | 124080000 | 1.825113 |
| ZNF326 | 14 | 14 | 11 | 13 | 11 | 13 | 26.5 | 65.653 | 582 | 0 | 149.54 | 24.9 | 24.2 | 179600000 | 144800000 | 1.240331 |
| TUBB | 22 | 4 | 22 | 21 | 4 | 4 | 64.3 | 47.766 | 426 | 0 | 161.93 | 64.3 | 62.4 | 178710000 | 163830000 | 1.090826 |
| DDX3X | 28 | 27 | 26 | 26 | 25 | 25 | 54.2 | 69.047 | 622 | 0 | 321.94 | 52.4 | 52.4 | 151330000 | 119850000 | 1.262662 |
| HNRNPH3 | 10 | 10 | 9 | 9 | 9 | 9 | 48.6 | 36.926 | 346 | 0 | 177.89 | 42.5 | 46 | 148340000 | 98149000 | 1.511376 |
| HNRNPUL1 | 23 | 23 | 23 | 17 | 23 | 17 | 43.3 | 85.939 | 766 | 0 | 323.31 | 43.3 | 35.1 | 103590000 | 83548000 | 1.239886 |
| HNRNPK | 18 | 18 | 18 | 17 | 18 | 17 | 50.5 | 50.976 | 463 | 0 | 210.86 | 50.5 | 48.2 | 102940000 | 120330000 | 0.855481 |
| PABPC1 | 20 | 12 | 19 | 19 | 12 | 12 | 44.1 | 58.535 | 522 | 0 | 253.66 | 42 | 42.3 | 95143000 | 120610000 | 0.788848 |
| ILF3 | 28 | 26 | 20 | 26 | 19 | 24 | 43 | 95.337 | 894 | 0 | 239.38 | 31.4 | 42.6 | 93893000 | 114050000 | 0.823262 |
| TAF15 | 12 | 10 | 12 | 11 | 10 | 10 | 26.2 | 61.829 | 592 | 0 | 162.71 | 26.2 | 26.2 | 83096000 | 59577000 | 1.394766 |
| ACTB | 20 | 9 | 10 | 20 | 4 | 9 | 69.1 | 41.736 | 375 | 0 | 230.5 | 41.1 | 69.1 | 80289000 | 445670000 | 0.180153 |
| SEC16A | 31 | 31 | 29 | 28 | 29 | 28 | 19 | 249.48 | 2334 | 0 | 263.73 | 17.7 | 18 | 79233000 | 82120000 | 0.964844 |
| EEF1A1P5 | 9 | 9 | 9 | 6 | 9 | 6 | 34.2 | 50.184 | 462 | 0 | 69.257 | 34.2 | 22.3 | 78013000 | 54224000 | 1.438717 |
| KHSRP | 21 | 18 | 18 | 18 | 15 | 16 | 36.4 | 73.027 | 711 | 0 | 201.3 | 32.2 | 33.9 | 76290000 | 65374000 | 1.166978 |
| AKAP8 | 20 | 20 | 19 | 16 | 19 | 16 | 38 | 76.107 | 692 | 0 | 236.39 | 36.8 | 31.8 | 76269000 | 63154000 | 1.207667 |
| RPS2 | 11 | 11 | 10 | 10 | 10 | 10 | 41.3 | 31.324 | 293 | 0 | 95.009 | 34.8 | 38.6 | 72922000 | 75696000 | 0.963353 |
| HNRNPU | 22 | 22 | 19 | 21 | 19 | 21 | 35.3 | 88.317 | 804 | 0 | 257.72 | 32.1 | 31.6 | 69716000 | 70236000 | 0.992596 |
| HNRNPA2B1 | 11 | 10 | 11 | 9 | 10 | 8 | 36 | 37.429 | 353 | 0 | 111.68 | 36 | 32.6 | 64029000 | 57973000 | 1.104462 |
| RPS18 | 8 | 8 | 7 | 7 | 7 | 7 | 45.4 | 17.718 | 152 | 0 | 51.45 | 40.8 | 45.4 | 63426000 | 27596000 | 2.298377 |
| PCBP1 | 14 | 11 | 13 | 8 | 11 | 6 | 67.7 | 37.497 | 356 | 0 | 153.87 | 65.4 | 32.6 | 62842000 | 46134000 | 1.362162 |
| DDX5 | 25 | 19 | 24 | 21 | 18 | 16 | 41.7 | 69.086 | 614 | 0 | 199.33 | 41.2 | 38.6 | 60991000 | 84042000 | 0.72572 |
| IGF2BP1 | 16 | 14 | 13 | 15 | 11 | 13 | 33.6 | 63.48 | 577 | 0 | 142.33 | 27 | 32.2 | 60586000 | 73104000 | 0.828764 |
| RPL6 | 14 | 14 | 12 | 12 | 12 | 12 | 38.2 | 32.728 | 288 | 0 | 140.69 | 34.7 | 35.1 | 58020000 | 60120000 | 0.96507 |
| EMD | 9 | 9 | 9 | 7 | 9 | 7 | 41.7 | 28.994 | 254 | 0 | 150.84 | 41.7 | 34.6 | 54699000 | 38764000 | 1.411077 |
| RPL4 | 15 | 15 | 12 | 14 | 12 | 14 | 39.6 | 47.697 | 427 | 0 | 137.66 | 34.4 | 38.2 | 54466000 | 72541000 | 0.750831 |
| HNRNPH2 | 11 | 7 | 11 | 10 | 7 | 6 | 38.3 | 49.263 | 449 | 0 | 129.51 | 38.3 | 34.1 | 53046000 | 54231000 | 0.978149 |
| RPL3 | 15 | 15 | 12 | 14 | 12 | 14 | 36 | 46.108 | 403 | 0 | 111.63 | 33.5 | 36 | 52295000 | 48736000 | 1.073026 |
| BAG4 | 12 | 12 | 12 | 11 | 12 | 11 | 46.4 | 49.593 | 457 | 0 | 234.52 | 46.4 | 46.4 | 51322000 | 31955000 | 1.606071 |
| AKAP8L | 10 | 10 | 10 | 10 | 10 | 10 | 20.7 | 71.648 | 646 | 0 | 122.14 | 20.7 | 20.7 | 51190000 | 52086000 | 0.982798 |
| RPS9 | 10 | 10 | 8 | 10 | 8 | 10 | 41.2 | 22.591 | 194 | 0 | 70.906 | 33 | 41.2 | 49418000 | 58267000 | 0.84813 |
| RPS4X | 16 | 16 | 12 | 15 | 12 | 15 | 55.5 | 29.597 | 263 | 0 | 181.27 | 44.5 | 52.5 | 49032000 | 61113000 | 0.802317 |

| | | | | | | | | | | | | | | | | |
|---|---|---|---|---|---|---|---|---|---|---|---|---|---|---|---|---|
| NPM1 | 9 | 9 | 8 | 8 | 8 | 8 | 42.2 | 32.575 | 294 | 0 | 132.76 | 42.2 | 39.8 | 47535000 | 48346000 | 0.983225 |
| RPS17 | 8 | 8 | 8 | 8 | 8 | 8 | 13.2 | 64.532 | 584 | 0 | 85.394 | 13.2 | 13.2 | 47468000 | 58042000 | 0.817822 |
| RPS8 | 10 | 10 | 10 | 9 | 10 | 9 | 49 | 24.205 | 208 | 0 | 137.42 | 49 | 45.7 | 45866000 | 59768000 | 0.767401 |
| RPS14 | 8 | 8 | 6 | 7 | 6 | 7 | 40.7 | 16.159 | 150 | 0 | 63.613 | 39.3 | 39.3 | 45638000 | 48204000 | 0.946768 |
| RPL10 | 9 | 2 | 7 | 9 | 1 | 2 | 42 | 22.975 | 200 | 0 | 78.073 | 40.5 | 42 | 45425000 | 39359000 | 1.15412 |
| RPL13 | 5 | 5 | 5 | 5 | 5 | 5 | 24.2 | 24.261 | 211 | 0 | 54.845 | 24.2 | 24.2 | 44762000 | 49726000 | 0.900173 |
| RPL7 | | 13 | 11 | 12 | 11 | 12 | 48 | 29.225 | 248 | 0 | 92.598 | 45.2 | 47.6 | 42367000 | 46253000 | 0.915984 |
| RPS16 | 8 | 8 | 7 | 8 | 7 | 8 | 51.4 | 16.445 | 146 | 0 | 52.978 | 45.9 | 51.4 | 42285000 | 28288000 | 1.494803 |
| SERBP1 | 12 | 12 | 11 | 9 | 11 | 9 | 35.5 | 44.965 | 408 | 0 | 193.05 | 32.4 | 23.5 | 42248000 | 22465000 | 1.880614 |
| RPS3 | 14 | 14 | 14 | 6 | 14 | 6 | 60.9 | 26.688 | 243 | 0 | 107.74 | 60.9 | 32.5 | 42050000 | 24873000 | 1.690588 |
| RPL15 | 10 | 10 | 9 | 9 | 9 | 9 | 43.6 | 24.146 | 204 | 0 | 85.021 | 43.6 | 43.1 | 40982000 | 55993000 | 0.731913 |
| HSPA8 | 16 | 14 | 15 | 9 | 13 | 7 | 28.6 | 70.897 | 646 | 0 | 136.33 | 26.9 | 17.8 | 40660000 | 19242000 | 2.113086 |
| HSPA1B | 15 | 8 | 15 | 11 | 8 | 5 | 28 | 70.108 | 642 | 0 | 143.42 | 28 | 23.4 | 40011000 | 30440000 | 1.314422 |
| DDX1 | 20 | 20 | 18 | 15 | 18 | 15 | 35 | 82.431 | 740 | 0 | 158.06 | 33.6 | 26.8 | 36241000 | 25059000 | 1.446227 |
| YLPM1 | 20 | 20 | 17 | 20 | 17 | 20 | 11.4 | 241.64 | 2146 | 0 | 148.35 | 11 | 11.4 | 34450000 | 36474000 | 0.944508 |
| RPL27A | 7 | 7 | 6 | 6 | 6 | 6 | 35.1 | 16.561 | 148 | 0 | 58.38 | 28.4 | 29.7 | 34108000 | 40960000 | 0.832715 |
| RPS3A | 7 | 7 | 7 | 7 | 7 | 7 | 36.8 | 25.608 | 223 | 0 | 71.211 | 36.8 | 36.8 | 34094000 | 35168000 | 0.969461 |
| HNRNPL | 13 | 13 | 12 | 12 | 12 | 12 | 38.9 | 58.362 | 530 | 0 | 156.96 | 37 | 36.4 | 33902000 | 52186000 | 0.649638 |
| RTCB | 17 | 17 | 15 | 11 | 15 | 11 | 43.4 | 55.21 | 505 | 0 | 169.02 | 38.8 | 32.5 | 32535000 | 25051000 | 1.298751 |
| RPS6 | 6 | 6 | 4 | 5 | 4 | 5 | 21.7 | 28.68 | 249 | 0 | 83.38 | 18.5 | 18.5 | 32120000 | 42990000 | 0.747151 |
| RPS19 | 9 | 9 | 9 | 3 | 9 | 3 | 49 | 16.06 | 145 | 0 | 61.249 | 49 | 19.3 | 31965000 | 8870700 | 3.603436 |
| HIST1H4A | 8 | 8 | 8 | 6 | 8 | 6 | 59.2 | 11.367 | 103 | 0 | 69.538 | 59.2 | 57.3 | 30989000 | 36335000 | 0.852869 |
| RPL34 | 5 | 5 | 4 | 5 | 4 | 5 | 30.8 | 13.293 | 117 | 0 | 32.528 | 30.8 | 30.8 | 30790000 | 27025000 | 1.139315 |
| RPL19 | 5 | 5 | 2 | 5 | 2 | 5 | 19.2 | 23.134 | 193 | 0 | 53.564 | 13.5 | 19.2 | 29256000 | 35041000 | 0.834908 |
| DHX9 | 23 | 23 | 20 | 19 | 20 | 19 | 24.3 | 140.96 | 1270 | 0 | 217.69 | 21.3 | 20.5 | 29080000 | 40533000 | 0.71744 |
| TUBB8 | 11 | 2 | 11 | 10 | 2 | 2 | 24.5 | 49.775 | 444 | 0 | 28.348 | 24.5 | 22.7 | 28881000 | 20778000 | 1.38998 |
| RPL7A | 10 | 10 | 8 | 8 | 8 | 8 | 34.6 | 29.995 | 266 | 0 | 71.741 | 30.8 | 34.2 | 28586000 | 28947000 | 0.987529 |
| ILF2 | 7 | 7 | 6 | 7 | 6 | 7 | 26.7 | 38.91 | 352 | 0 | 90.085 | 23.6 | 26.7 | 28547000 | 26944000 | 1.059494 |
| SLC25A5 | 9 | 4 | 8 | 5 | 4 | 2 | 27.2 | 32.852 | 298 | 0 | 87.621 | 24.5 | 16.8 | 27803000 | 11181000 | 2.486629 |
| PRDX1 | 8 | 8 | 8 | 7 | 8 | 7 | 44.7 | 22.11 | 199 | 0 | 63.261 | 44.7 | 39.2 | 27701000 | 13575000 | 2.040589 |
| RPL12 | 5 | 5 | 5 | 4 | 5 | 4 | 45.5 | 17.818 | 165 | 0 | 110.53 | 45.5 | 35.8 | 26941000 | 22337000 | 1.206115 |
| HIST1H1C | 5 | 5 | 4 | 5 | 4 | 5 | 15 | 21.364 | 213 | 0 | 36.067 | 10.8 | 15 | 26828000 | 39664000 | 0.676382 |
| RPSA | 8 | 8 | 8 | 4 | 8 | 4 | 51.3 | 29.404 | 263 | 0 | 107.19 | 51.3 | 27.8 | 25891000 | 14349000 | 1.804377 |
| RPS11 | 7 | 7 | 7 | 6 | 7 | 6 | 43 | 18.431 | 158 | 0 | 66.812 | 43 | 36.7 | 24350000 | 26080000 | 0.933666 |
| RPS25 | 3 | 3 | 2 | 3 | 2 | 3 | 24 | 13.742 | 125 | 0 | 19.715 | 16 | 24 | 23872000 | 15390000 | 1.551137 |
| FUS | 8 | 6 | 7 | 7 | 5 | 6 | 21.7 | 53.425 | 526 | 0 | 60.994 | 21.5 | 21.7 | 23623000 | 12272000 | 1.924951 |
| DDX17 | 20 | 14 | 19 | 16 | 13 | 11 | 35.1 | 80.253 | 729 | 0 | 125.44 | 33.2 | 30 | 23269000 | 28620000 | 0.813033 |
| HNRNPDL | 10 | 9 | 8 | 9 | 7 | 8 | 22.3 | 40.04 | 363 | 0 | 136.18 | 22 | 22.3 | 23233000 | 33961000 | 0.684108 |
| RPL8 | 8 | 8 | 6 | 7 | 6 | 7 | 34.2 | 28.024 | 257 | 0 | 73.97 | 28.4 | 31.1 | 23055000 | 25181000 | 0.915571 |
| LARP1 | 19 | 19 | 13 | 17 | 13 | 17 | 32.7 | 123.51 | 1096 | 0 | 194.17 | 20.3 | 27.8 | 22922000 | 32347000 | 0.708628 |
| RPL27 | 4 | 4 | 4 | 4 | 4 | 4 | 36 | 15.798 | 136 | 0 | 29.945 | 36 | 36 | 22581000 | 18387000 | 1.228096 |
| MOV10 | 21 | 21 | 14 | 21 | 14 | 21 | 30.1 | 107.21 | 947 | 0 | 241.13 | 17.8 | 30.1 | 21779000 | 26083000 | 0.834988 |
| RPLP0 | 6 | 6 | 6 | 6 | 6 | 6 | 30.3 | 34.273 | 317 | 0 | 43.519 | 30.3 | 30.3 | 21040000 | 23989000 | 0.877069 |
| CP | 8 | 8 | 8 | 6 | 8 | 6 | 7.9 | 122.2 | 1065 | 0 | 72.576 | 7.9 | 5.9 | 21032000 | 19930000 | 1.055294 |
| RPL24 | 5 | 5 | 3 | 4 | 3 | 4 | 39.7 | 14.369 | 121 | 0 | 41.999 | 25.6 | 31.4 | 20917000 | 29614000 | 0.706321 |
| GNB2L1 | 10 | 10 | 10 | 5 | 10 | 5 | 47.3 | 35.076 | 317 | 0 | 82.278 | 47.3 | 19.9 | 20827000 | 7581900 | 2.746937 |
| RPL11 | 6 | 6 | 6 | 4 | 6 | 4 | 33.1 | 20.252 | 178 | 0 | 64.107 | 33.1 | 24.2 | 20774000 | 24321000 | 0.854159 |
| DDX21 | 17 | 17 | 13 | 16 | 13 | 16 | 29.5 | 87.343 | 783 | 0 | 186.21 | 23.4 | 27.2 | 20769000 | 33245000 | 0.624726 |
| RPL36A | 5 | 2 | 3 | 5 | 1 | 2 | 25 | 13.228 | 112 | 0 | 32.367 | 17 | 25 | 20742000 | 28946000 | 0.716576 |
| YBX1 | 7 | 4 | 6 | 7 | 3 | 4 | 29.3 | 35.924 | 324 | 0 | 105.51 | 22.2 | 29.3 | 20360000 | 23726000 | 0.85813 |
| PTBP1 | 10 | 10 | 8 | 9 | 8 | 9 | 26.2 | 62.463 | 588 | 0 | 99.78 | 20.6 | 24 | 20332000 | 35221000 | 0.577269 |
| GPRASP2 | 9 | 9 | 9 | 3 | 9 | 3 | 13 | 93.772 | 838 | 0 | 81.907 | 13 | 4.2 | 20064000 | 10947000 | 1.832831 |
| RPL28 | 5 | 5 | 5 | 3 | 5 | 3 | 22.9 | 19.072 | 170 | 0 | 35.484 | 22.9 | 17.6 | 19966000 | 21962000 | 0.909116 |
| MATR3 | 18 | 15 | 15 | 17 | 12 | 14 | 28.3 | 94.622 | 847 | 0 | 162.42 | 22 | 28.3 | 19886000 | 26035000 | 0.763818 |
| RCN2 | 9 | 9 | 8 | 8 | 8 | 8 | 33.4 | 36.876 | 317 | 0 | 132.34 | 30.9 | 28.4 | 19845000 | 21968000 | 0.903359 |
| CTTN | 12 | 12 | 11 | 8 | 11 | 8 | 21.6 | 61.585 | 550 | 0 | 94.956 | 18.9 | 15.3 | 19594000 | 15383000 | 1.273744 |
| HIST1H2BN | 5 | 5 | 3 | 5 | 3 | 5 | 31.3 | 18.804 | 166 | 0 | 54.257 | 21.7 | 31.3 | 19551000 | 18586000 | 1.051921 |
| RPL14 | 4 | 4 | 4 | 4 | 4 | 4 | 36.3 | 14.558 | 124 | 0 | 44.35 | 36.3 | 36.3 | 19396000 | 16539000 | 1.172743 |
| HNRNPD | 9 | 8 | 6 | 9 | 5 | 8 | 38.6 | 23.811 | 210 | 0 | 81.765 | 32.4 | 38.6 | 19370000 | 31295000 | 0.618949 |
| RPS20 | 4 | 4 | 4 | 3 | 4 | 3 | 28.6 | 13.373 | 119 | 0 | 28.74 | 28.6 | 22.7 | 19283000 | 17954000 | 1.074023 |
| KHDRBS3 | 8 | 6 | 7 | 6 | 5 | 4 | 21.7 | 38.799 | 346 | 0 | 38.999 | 19.4 | 16.2 | 19271000 | 18337000 | 1.050935 |
| RBMX | 12 | 6 | 9 | 9 | 4 | 5 | 28.4 | 42.331 | 391 | 0 | 99.881 | 24.3 | 21 | 19212000 | 19351000 | 0.992817 |
| RPL18 | 5 | 5 | 4 | 4 | 4 | 4 | 28 | 18.756 | 164 | 0 | 48.436 | 22 | 27.4 | 19108000 | 17068000 | 1.119522 |
| PABPC4 | 17 | 9 | 14 | 15 | 7 | 8 | 33.3 | 67.97 | 615 | 0 | 66.323 | 27.2 | 30.1 | 18257000 | 27932000 | 0.653623 |
| NCL | 11 | 11 | 10 | 9 | 10 | 9 | 18.9 | 76.613 | 710 | 0 | 120.7 | 17.7 | 17.7 | 17897000 | 17870000 | 1.001511 |
| HIST1H2AJ | 2 | 2 | 2 | 2 | 2 | 2 | 16.6 | 18.481 | 169 | 0 | 19.56 | 16.6 | 16.6 | 17613000 | 45579000 | 0.386428 |
| RPL10A | 7 | 7 | 6 | 3 | 6 | 3 | 35 | 24.831 | 217 | 0 | 40.995 | 31.3 | 17.1 | 17491000 | 13757000 | 1.271425 |
| FGG | 4 | 4 | 4 | 4 | 4 | 4 | 12.1 | 50.322 | 445 | 0 | 136.7 | 12.1 | 12.1 | 17487000 | 16455000 | 1.062716 |
| HNRNPR | 17 | 10 | 8 | 15 | 5 | 8 | 29.2 | 70.942 | 633 | 0 | 119.87 | 16.6 | 24.8 | 17424000 | 23580000 | 0.738931 |
| SRSF1 | 10 | 9 | 7 | 10 | 6 | 9 | 37.9 | 28.329 | 253 | 0 | 80.847 | 28.1 | 37.9 | 17413000 | 22368000 | 0.778478 |
| RPL5 | 8 | 8 | 7 | 5 | 7 | 5 | 33.6 | 27.045 | 238 | 0 | 54.506 | 33.6 | 22.7 | 17058000 | 12724000 | 1.340616 |
| PRCC | 8 | 8 | 7 | 8 | 7 | 8 | 27.1 | 52.417 | 491 | 0 | 103.89 | 24.8 | 27.1 | 16307000 | 17070000 | 0.955302 |
| RPL31 | 3 | 3 | 3 | 2 | 3 | 2 | 26.4 | 14.463 | 125 | 0 | 37.514 | 26.4 | 18.4 | 15652000 | 30812000 | 0.507984 |
| SRSF7 | 6 | 5 | 6 | 6 | 5 | 5 | 43.1 | 15.763 | 137 | 0 | 42.074 | 43.1 | 43.1 | 15507000 | 25121000 | 0.617292 |
| CTNND1 | 6 | 6 | 6 | 6 | 6 | 6 | 9.3 | 104.85 | 938 | 0 | 47.322 | 9.3 | 9.3 | 15318000 | 12128000 | 1.263028 |
| RPL21 | 5 | 5 | 3 | 5 | 3 | 5 | 30.6 | 18.565 | 160 | 0 | 46.958 | 25 | 30.6 | 15198000 | 21566000 | 0.70472 |
| KRT1 | 15 | 11 | 7 | 14 | 5 | 10 | 25.2 | 66.038 | 644 | 0 | 202.18 | 13.8 | 22.5 | 14902000 | 56639000 | 0.263105 |
| SRSF6 | 4 | 4 | 3 | 4 | 3 | 4 | 11.9 | 39.586 | 344 | 0 | 31.268 | 8.7 | 11.9 | 14643000 | 13928000 | 1.051335 |
| FGB | 8 | 8 | 8 | 5 | 8 | 5 | 29 | 31.18 | 272 | 0 | 94.432 | 29 | 22.4 | 14624000 | 11488000 | 1.272981 |
| SF1 | 6 | 5 | 5 | 6 | 4 | 5 | 15.6 | 68.329 | 639 | 0 | 56.555 | 14.1 | 15.6 | 14529000 | 14245000 | 1.019937 |
| RPS26 | 4 | 4 | 4 | 4 | 4 | 4 | 37.4 | 13.015 | 115 | 0 | 34.776 | 37.4 | 37.4 | 14193000 | 19012000 | 0.746529 |
| RPS5 | 6 | 6 | 5 | 5 | 5 | 5 | 36.5 | 22.391 | 200 | 0 | 76.538 | 32 | 31.5 | 13894000 | 7867900 | 1.76591 |
| RPS15A | 4 | 4 | 4 | 4 | 4 | 4 | 38 | 11.477 | 100 | 0 | 39.38 | 38 | 38 | 13741000 | 12481000 | 1.100953 |
| RPL18A | 8 | 8 | 5 | 8 | 5 | 8 | 39.2 | 20.762 | 176 | 0 | 62.763 | 26.1 | 39.2 | 13608000 | 10958000 | 1.241832 |
| CALU | 10 | 10 | 9 | 8 | 9 | 8 | 36.2 | 37.106 | 315 | 0 | 109.27 | 36.2 | 30.5 | 13397000 | 13261000 | 1.010256 |
| RPL17 | 4 | 4 | 4 | 4 | 4 | 4 | 24.3 | 19.586 | 169 | 0 | 44.536 | 24.3 | 24.3 | 13387000 | 12719000 | 1.05252 |
| RPL23A | 6 | 6 | 5 | 5 | 5 | 5 | 28.5 | 17.692 | 158 | 0 | 54.487 | 27.2 | 20.3 | 12715000 | 22184000 | 0.573161 |
| DHX30 | 18 | 18 | 14 | 14 | 14 | 14 | 20.8 | 130.55 | 1166 | 0 | 173.94 | 16 | 17.2 | 12615000 | 13991000 | 0.901651 |
| RPS24 | 4 | 4 | 4 | 4 | 4 | 4 | 29.8 | 15.197 | 131 | 0 | 42.189 | 29.8 | 29.8 | 12593000 | 13049000 | 0.965055 |
| RPS7 | 6 | 6 | 5 | 5 | 5 | 5 | 47.4 | 22.127 | 194 | 0 | 51.775 | 43.3 | 42.8 | 12168000 | 14442000 | 0.842543 |
| HSPD1 | 10 | 10 | 10 | 5 | 10 | 5 | 21.1 | 61.054 | 573 | 0 | 78.379 | 21.1 | 9.9 | 12100000 | 5720000 | 2.115385 |
| RPL23 | 3 | 3 | 3 | 2 | 3 | 2 | 47.3 | 9.6694 | 91 | 0 | 26.641 | 47.3 | 38.5 | 11852000 | 19018000 | 0.623199 |
| HNRNPA3 | 5 | 5 | 4 | 5 | 4 | 5 | 19.6 | 39.594 | 378 | 0 | 64.994 | 15.6 | 19.6 | 11740000 | 12009000 | 0.9776 |
| RPL26 | 4 | 4 | 2 | 4 | 2 | 4 | 25.2 | 12.63 | 107 | 0 | 25.912 | 16.8 | 25.2 | 11515000 | 10368000 | 1.110629 |
| H3F3B | 3 | 3 | 3 | 3 | 3 | 3 | 25 | 10.334 | 92 | 0 | 17.348 | 25 | 25 | 11485000 | 9623900 | 1.193383 |
| RPL35A | 2 | 2 | 2 | 2 | 2 | 2 | 22.3 | 10.645 | 94 | 0 | 20.098 | 22.3 | 22.3 | 11235000 | 14436000 | 0.778263 |
| U2AF2 | 7 | 7 | 6 | 6 | 6 | 6 | 24.4 | 53.5 | 475 | 0 | 94.595 | 22.5 | 22.1 | 11062000 | 18201000 | 0.607769 |
| ELAVL1 | 5 | 5 | 4 | 5 | 4 | 5 | 24.2 | 36.091 | 326 | 0 | 64.393 | 16.6 | 24.2 | 10965000 | 14798000 | 0.740979 |
| HSP90AB1 | 11 | 8 | 11 | 6 | 8 | 4 | 18.5 | 83.263 | 724 | 0 | 186.68 | 18.5 | 9.7 | 10789000 | 6568000 | 1.642661 |
| RPS13 | 5 | 5 | 4 | 4 | 4 | 4 | 33.8 | 16.733 | 148 | 0 | 44.862 | 27 | 25.7 | 10778000 | 12790000 | 0.84269 |
| SRSF3 | 4 | 3 | 4 | 3 | 3 | 2 | 40 | 10.32 | 95 | 0 | 18.631 | 40 | 31.6 | 10740000 | 18920000 | 0.567653 |
| UPF1 | 14 | 14 | 6 | 14 | 6 | 14 | 17 | 124.34 | 1129 | 0 | 141.24 | 8 | 17 | 10626000 | 12812000 | 0.829379 |
| TUBB2A | 20 | 1 | 20 | 18 | 1 | 1 | 60.2 | 49.906 | 445 | 0 | 38.704 | 60.2 | 55.1 | 10488000 | 11491000 | 0.912714 |
| HNRNPM | 8 | 8 | 6 | 6 | 6 | 6 | 14.5 | 77.569 | 730 | 0 | 56.914 | 11.5 | 11.1 | 10215000 | 7978500 | 1.280316 |
| FAM98A | 8 | 8 | 8 | 6 | 8 | 6 | 22.5 | 55.4 | 519 | 0 | 125.18 | 22.5 | 18.3 | 10212000 | 9934400 | 1.027943 |
| TIAL1 | 6 | 4 | 6 | 6 | 4 | 4 | 22.9 | 41.59 | 375 | 0 | 63.112 | 22.9 | 22.9 | 10080000 | 9358700 | 1.077073 |
| SFPQ | 10 | 9 | 5 | 9 | 5 | 8 | 18.8 | 76.149 | 707 | 0 | 90.166 | 7.9 | 18.7 | 9860600 | 13377000 | 0.737131 |
| HNRNPAB | 4 | 4 | 4 | 4 | 4 | 4 | 15.4 | 30.302 | 280 | 0 | 66.891 | 15.4 | 15.4 | 9782500 | 17996000 | 0.543593 |
| HNRNPA1 | 9 | 8 | 8 | 8 | 7 | 7 | 28 | 33.155 | 307 | 0 | 72.358 | 25.4 | 28 | 9668200 | 16309000 | 0.592814 |

| | | | | | | | | | | | | | | | | |
|---|---|---|---|---|---|---|---|---|---|---|---|---|---|---|---|---|
| RPL32 | 4 | 4 | 4 | 2 | 4 | 2 | 33.1 | 15.616 | 133 | 0 | 55.496 | 33.1 | 20.3 | 9459800 | 10478000 | 0.902825 |
| SF1 | 4 | 1 | 4 | 3 | 1 | 1 | 28.3 | 18.639 | 180 | 0 | 43.985 | 28.3 | 23.9 | 9445600 | 9158400 | 1.031359 |
| C14orf166 | 7 | 7 | 7 | 5 | 7 | 5 | 35.2 | 28.068 | 244 | 0 | 78.859 | 35.2 | 27.9 | 9413600 | 6594700 | 1.427449 |
| RPS23 | 6 | 6 | 5 | 6 | 5 | 6 | 42 | 15.807 | 143 | 0 | 43.895 | 42 | 42 | 9322100 | 18076000 | 0.515717 |
| SYNCRIP | 16 | 9 | 10 | 16 | 7 | 9 | 28.4 | 69.602 | 623 | 0 | 155.98 | 20.7 | 28.4 | 9234000 | 18414000 | 0.501466 |
| CNN2 | 5 | 5 | 5 | 5 | 5 | 5 | 26.8 | 32.616 | 298 | 0 | 66.069 | 26.8 | 26.8 | 9229800 | 4255200 | 2.169064 |
| EEF2 | 13 | 13 | 12 | 6 | 12 | 6 | 19.2 | 95.337 | 858 | 0 | 104.87 | 18.1 | 10 | 8900900 | 7456800 | 1.193662 |
| CNN3 | 5 | 5 | 5 | 3 | 5 | 3 | 19.1 | 36.413 | 329 | 0 | 37.612 | 19.1 | 10 | 8750600 | 5210700 | 1.679352 |
| EIF4A3 | 7 | 5 | 6 | 4 | 4 | 2 | 19.2 | 46.871 | 411 | 0 | 61.398 | 16.1 | 11.7 | 8479200 | 7969000 | 1.064023 |
| RPL13A | 3 | 3 | 3 | 3 | 3 | 3 | 14.8 | 24.104 | 210 | 0 | 19.525 | 14.8 | 14.8 | 8407400 | 6647000 | 1.264841 |
| RCC2 | 8 | 8 | 7 | 6 | 7 | 6 | 19.3 | 56.084 | 522 | 0 | 58.77 | 18 | 13.8 | 8300200 | 6451000 | 1.286653 |
| RPL30 | 2 | 2 | 2 | 2 | 2 | 2 | 68.6 | 5.5553 | 51 | 0 | 70.347 | 68.6 | 68.6 | 7815600 | 12272000 | 0.636864 |
| SRSF2 | 5 | 5 | 5 | 2 | 5 | 2 | 45.1 | 15.371 | 133 | 0 | 32.09 | 45.1 | 14.3 | 7696900 | 8502900 | 0.905209 |
| NONO | 8 | 7 | 6 | 6 | 6 | 5 | 35 | 28.09 | 234 | 0 | 50.325 | 31.6 | 31.6 | 7498500 | 9107200 | 0.82336 |
| XRCC6 | 8 | 8 | 7 | 5 | 7 | 5 | 19.5 | 64.283 | 559 | 0 | 46.44 | 16.6 | 12.5 | 7187500 | 5527100 | 1.300411 |
| ACTA1 | 12 | 1 | 7 | 12 | 1 | 1 | 30.2 | 42.051 | 377 | 0 | 17.538 | 21.5 | 30.2 | 7019100 | 26634000 | 0.263539 |
| HNRNPA0 | 5 | 5 | 5 | 4 | 5 | 4 | 20.7 | 30.84 | 305 | 0 | 39.87 | 20.7 | 18.4 | 6721100 | 9872900 | 0.680762 |
| HNRNPUL2 | 5 | 5 | 2 | 5 | 2 | 5 | 7.4 | 85.104 | 747 | 0 | 32.778 | 2.9 | 7.4 | 6644900 | 7088800 | 0.93738 |
| PHGDH | 6 | 6 | 6 | 2 | 6 | 2 | 13.3 | 55.938 | 526 | 0 | 72.914 | 13.3 | 4.6 | 6559600 | 1983400 | 3.30725 |
| SLC25A3 | 4 | 4 | 4 | 2 | 4 | 2 | 18.8 | 36.161 | 324 | 0 | 26.055 | 18.8 | 8 | 6473600 | 2179700 | 2.96995 |
| TCF25 | 4 | 4 | 4 | 2 | 4 | 2 | 7.5 | 76.666 | 676 | 0 | 29.959 | 7.5 | 4.7 | 6350800 | 1817600 | 3.494058 |
| IGKV A18 | 2 | 2 | 2 | 2 | 2 | 2 | 16.7 | 13.085 | 120 | 0 | 56.493 | 16.7 | 16.7 | 6322900 | 7521800 | 0.84061 |
| PCBP2 | 7 | 4 | 5 | 5 | 3 | 3 | 35.1 | 38.58 | 365 | 0 | 26.739 | 29 | 21.1 | 6151400 | 3171900 | 1.939342 |
| U2AF1 | 7 | 7 | 5 | 6 | 5 | 6 | 31.7 | 27.872 | 240 | 0 | 89.898 | 22.1 | 30.8 | 6085600 | 8654700 | 0.703156 |
| TUFM | 8 | 8 | 8 | 6 | 8 | 6 | 27 | 49.541 | 452 | 0 | 61.472 | 27 | 21.2 | 6082200 | 5297800 | 1.148061 |
| PLG | 2 | 2 | 2 | 2 | 2 | 2 | 2.2 | 90.568 | 810 | 0 | 12.65 | 2.2 | 2.2 | 5920900 | 3316000 | 1.785555 |
| RPS27 | 4 | 4 | 2 | 4 | 2 | 4 | 40.5 | 9.461 | 84 | 0 | 24.017 | 29.8 | 40.5 | 5816100 | 4562700 | 1.274706 |
| LRRC59 | 6 | 6 | 4 | 4 | 4 | 4 | 30.6 | 34.93 | 307 | 0 | 37.592 | 22.1 | 19.2 | 5757300 | 2079100 | 2.769131 |
| RPL22 | 2 | 2 | 2 | 2 | 2 | 2 | 28.9 | 14.787 | 128 | 0 | 24.911 | 28.9 | 28.9 | 5659300 | 13173000 | 0.429614 |
| KRT9 | 10 | 10 | 3 | 9 | 3 | 9 | 25.2 | 62.064 | 623 | 0 | 142.17 | 6.1 | 23.1 | 5593100 | 18815000 | 0.297268 |
| RPL37A | 3 | 3 | 3 | 2 | 3 | 2 | 38.9 | 8.0627 | 72 | 0 | 20.325 | 38.9 | 29.2 | 5533300 | 6709200 | 0.824733 |
| HNRNPC | 5 | 5 | 4 | 5 | 4 | 5 | 19 | 25.256 | 231 | 0 | 32.684 | 18.6 | 19 | 5491300 | 5925400 | 0.926739 |
| RPS29 | 3 | 3 | 2 | 3 | 2 | 3 | 46.4 | 6.6767 | 56 | 0 | 18.564 | 26.8 | 46.4 | 5392500 | 3410900 | 1.580961 |
| ECM1 | 2 | 2 | 2 | 2 | 2 | 2 | 4.8 | 60.673 | 540 | 0 | 30.982 | 4.8 | 4.8 | 5203800 | 2996700 | 1.73651 |
| DHX15 | 8 | 8 | 6 | 7 | 6 | 7 | 11.9 | 90.932 | 795 | 0 | 63.996 | 8.8 | 10.6 | 5203000 | 5551500 | 0.937224 |
| EYA4 | 5 | 5 | 5 | 2 | 5 | 2 | 13.5 | 61.993 | 570 | 0 | 45.006 | 13.5 | 5.4 | 5127300 | 4613900 | 1.111272 |
| IGF2BP3 | 10 | 8 | 8 | 9 | 6 | 7 | 24.5 | 63.704 | 579 | 0 | 87.133 | 19.9 | 22.3 | 5000800 | 5727200 | 0.873167 |
| IGF2 | 2 | 2 | 2 | 2 | 2 | 2 | 13.9 | 20.14 | 180 | 0 | 45.217 | 13.9 | 13.9 | 4932500 | 4094100 | 1.204782 |
| FXR1 | 7 | 6 | 4 | 5 | 3 | 5 | 23.1 | 50.99 | 454 | 0 | 48.276 | 13 | 18.3 | 4710300 | 10990000 | 0.428599 |
| KPNA2 | 5 | 5 | 5 | 4 | 5 | 4 | 15.3 | 57.861 | 529 | 0 | 103.26 | 15.3 | 13.2 | 4527600 | 6485100 | 0.698154 |
| ZCCHC3 | 9 | 9 | 4 | 8 | 4 | 8 | 27.7 | 43.618 | 404 | 0 | 151.48 | 12.9 | 25.5 | 4493100 | 4776500 | 0.940668 |
| EWSR1 | 3 | 3 | 3 | 3 | 3 | 3 | 7.2 | 61.414 | 584 | 0 | 61.398 | 7.2 | 7.2 | 4426800 | 4905200 | 0.902471 |
| ATP5A1 | 5 | 5 | 4 | 3 | 4 | 3 | 12.7 | 59.75 | 553 | 0 | 35.828 | 8.9 | 8.5 | 4321500 | 4284000 | 1.008754 |
| HSPA9 | 7 | 7 | 7 | 3 | 7 | 3 | 13.7 | 73.68 | 679 | 0 | 45.311 | 13.7 | 6.3 | 4318000 | 2949100 | 1.464176 |
| SNX9 | 6 | 6 | 6 | 4 | 6 | 4 | 15.8 | 66.591 | 595 | 0 | 163.38 | 15.8 | 10.4 | 4291300 | 2994400 | 1.433108 |
| RPS15 | 4 | 4 | 4 | 4 | 4 | 4 | 39.3 | 12.99 | 112 | 0 | 28.123 | 39.3 | 39.3 | 4156400 | 4902500 | 0.847812 |
| PGAM5 | 4 | 4 | 3 | 3 | 3 | 3 | 17.6 | 32.004 | 289 | 0 | 25.39 | 14.5 | 13.5 | 4107100 | 6462800 | 0.635499 |
| N4BP2L2 | 3 | 3 | 3 | 2 | 3 | 2 | 5.6 | 82.367 | 709 | 0 | 33.381 | 5.6 | 3.7 | 4098000 | 3475600 | 1.179077 |
| EIF4A2 | 5 | 3 | 5 | 5 | 3 | 3 | 15.2 | 41.29 | 362 | 0 | 29.391 | 15.2 | 15.2 | 4021600 | 3452300 | 1.164905 |
| GNL3 | 5 | 5 | 4 | 4 | 4 | 4 | 13.5 | 61.992 | 549 | 0 | 32.353 | 11.7 | 11.5 | 3634900 | 3748500 | 0.969695 |
| CNBP | 6 | 6 | 4 | 5 | 4 | 5 | 41.8 | 19.463 | 177 | 0 | 71.382 | 25.4 | 33.3 | 3560500 | 3857200 | 0.923079 |
| IGFBP3 | 3 | 3 | 3 | 3 | 3 | 3 | 13.9 | 21.844 | 194 | 0 | 18.466 | 13.9 | 13.9 | 3558900 | 3147400 | 1.130743 |
| LUC7L2 | 4 | 4 | 3 | 4 | 3 | 4 | 10.3 | 54.223 | 458 | 0 | 27.143 | 8.5 | 10.3 | 3492000 | 3523300 | 0.991116 |
| SRRM2 | 7 | 7 | 5 | 5 | 5 | 5 | 4.5 | 299.61 | 2752 | 0 | 47.006 | 3.2 | 3.4 | 3433300 | 3887000 | 0.883278 |
| NCBP1 | 5 | 5 | 3 | 5 | 3 | 5 | 9.2 | 91.838 | 790 | 0 | 41.076 | 6.3 | 9.2 | 3304800 | 4148800 | 0.796568 |
| XRCC5 | 7 | 7 | 6 | 4 | 6 | 4 | 15.4 | 82.704 | 732 | 0 | 54.107 | 12.4 | 9 | 3223600 | 2907400 | 1.108757 |
| COL3A1 | 3 | 3 | 3 | 2 | 3 | 2 | 2.5 | 138.56 | 1466 | 0 | 23.207 | 2.5 | 1.6 | 3111100 | 2401300 | 1.29559 |
| SEC13 | 3 | 3 | 3 | 3 | 3 | 3 | 19.9 | 35.54 | 322 | 0 | 24.287 | 19.9 | 19.9 | 3017800 | 2873500 | 1.050218 |
| DAZAP1 | 3 | 3 | 3 | 2 | 3 | 2 | 14.4 | 35.02 | 327 | 0 | 18.624 | 14.4 | 9.5 | 2810200 | 3092200 | 0.908803 |
| FAM168A | 2 | 2 | 2 | 2 | 2 | 2 | 15.2 | 26.184 | 244 | 0 | 12.818 | 15.2 | 15.2 | 2696700 | 3357200 | 0.803259 |
| SNRPD3 | 2 | 2 | 2 | 2 | 2 | 2 | 15.1 | 13.916 | 126 | 0 | 12.099 | 15.1 | 15.1 | 2609400 | 2228100 | 1.171132 |
| RPL10 | 8 | 1 | 7 | 8 | 1 | 1 | 44.8 | 18.565 | 163 | 0 | 60.182 | 44.2 | 44.8 | 2600800 | 2129800 | 1.221148 |
| YTHDF2 | 5 | 4 | 4 | 3 | 3 | 2 | 8.8 | 62.333 | 579 | 0 | 39.302 | 7.6 | 4.8 | 2571600 | 5170300 | 0.497379 |
| NUDT21 | 4 | 4 | 4 | 2 | 4 | 2 | 15.9 | 26.227 | 227 | 0 | 23.23 | 15.9 | 7.5 | 2451300 | 2655100 | 0.923242 |
| H1FX | 4 | 4 | 3 | 3 | 3 | 3 | 22.5 | 22.487 | 213 | 0 | 30.52 | 16.9 | 17.4 | 2340400 | 3415600 | 0.685209 |
| FUBP3 | 6 | 5 | 4 | 4 | 3 | 4 | 19.1 | 61.64 | 572 | 0 | 35.887 | 14.9 | 13.5 | 2337400 | 2388300 | 0.978688 |
| SNRPC | 3 | 3 | 2 | 3 | 2 | 3 | 21.7 | 19.687 | 180 | 0 | 18.398 | 11.7 | 21.7 | 2232400 | 2620300 | 0.851964 |
| ATAD3A | 5 | 5 | 4 | 3 | 4 | 3 | 11.7 | 64.243 | 572 | 0 | 31.682 | 9.1 | 6.1 | 2201300 | 1284500 | 1.713741 |
| SSB | 3 | 3 | 3 | 3 | 3 | 3 | 9.3 | 46.836 | 408 | 0 | 26.74 | 9.3 | 9.3 | 2080000 | 2734300 | 0.760707 |
| RBM25 | 3 | 3 | 2 | 3 | 2 | 3 | 5.8 | 100.18 | 843 | 0 | 27.819 | 3.8 | 5.8 | 2078200 | 2081300 | 0.998511 |
| RAI1 | 3 | 3 | 2 | 3 | 2 | 3 | 5.2 | 101.43 | 942 | 0 | 21.619 | 3.9 | 5.2 | 1729300 | 2046100 | 0.845169 |
| RBM4 | 3 | 3 | 3 | 3 | 3 | 3 | 9.3 | 40.313 | 364 | 0 | 76.061 | 9.3 | 9.3 | 1718600 | 1089300 | 1.57771 |
| SNRPD2 | 2 | 2 | 2 | 2 | 2 | 2 | 16.9 | 13.527 | 118 | 0 | 13.521 | 16.9 | 16.9 | 1618200 | 1715200 | 0.943447 |
| PRPF38B | 3 | 3 | 2 | 3 | 2 | 3 | 4.9 | 64.467 | 546 | 0 | 22.166 | 3.3 | 4.9 | 1575400 | 1889500 | 0.833766 |
| KRT10 | 14 | 12 | 2 | 14 | 2 | 12 | 34.1 | 58.826 | 584 | 0 | 173.64 | 3.9 | 34.1 | 1571800 | 32322000 | 0.048629 |
| ZNF207 | 3 | 3 | 3 | 2 | 3 | 2 | 7.7 | 52.541 | 493 | 0 | 18.74 | 7.7 | 5.3 | 1567300 | 1922700 | 0.815156 |
| HNRNPDL | 1 | 1 | 1 | 1 | 1 | 1 | 31.6 | 6.7215 | 57 | 0 | 32.167 | 31.6 | 31.6 | 1546400 | 1305100 | 1.18489 |
| YWHAQ | 5 | 5 | 5 | 2 | 5 | 2 | 23.3 | 27.764 | 245 | 0 | 70.55 | 23.3 | 9 | 1475800 | 1399400 | 1.054595 |
| EIF2S3 | 2 | 2 | 2 | 2 | 2 | 2 | 9.1 | 51.109 | 472 | 0 | 14.682 | 9.1 | 9.1 | 1353700 | 1480800 | 0.914168 |
| MRPS27 | 2 | 2 | 2 | 2 | 2 | 2 | 6.4 | 34.458 | 299 | 0 | 12.883 | 6.4 | 6.4 | 972540 | 677460 | 1.435568 |
| LUC7L3 | 3 | 3 | 3 | 2 | 3 | 2 | 8.8 | 58.22 | 489 | 0 | 55.693 | 8.8 | 5.9 | 868360 | 1029700 | 0.843314 |
| TUBB4A | 20 | 1 | 20 | 19 | 1 | 1 | 58.3 | 49.585 | 444 | 0 | 28.432 | 58.3 | 57.9 | 668760 | 1126800 | 0.593504 |
| GRWD1 | 2 | 2 | 2 | 2 | 2 | 2 | 14 | 25.477 | 228 | 0 | 20.809 | 14 | 14 | 633130 | 706840 | 0.895719 |
| RBM7 | 1 | 1 | 1 | 1 | 1 | 1 | 7.5 | 17.223 | 146 | 0 | 7.3515 | 7.5 | 7.5 | 514810 | 854560 | 0.602427 |
| PPP1CC | 4 | 4 | 2 | 4 | 2 | 4 | 20.7 | 30.987 | 270 | 0 | 42.616 | 13 | 20.7 | 485850 | 597240 | 0.813492 |
| MTHFD1 | 1 | 1 | 1 | 0 | 1 | 0 | 1.2 | 110.61 | 1020 | 0 | 20.392 | 1.2 | 0 | 0 | 0 | #DIV/0! |
| FUSIP1 | 1 | 1 | 0 | 1 | 0 | 1 | 23.2 | 8.3294 | 69 | 0 | 7.7189 | 0 | 23.2 | 0 | 0 | #DIV/0! |
| AGO2 | 1 | 1 | 1 | 0 | 1 | 0 | 1.7 | 97.207 | 859 | 0.0025707 | 6.9616 | 1.7 | 0 | 0 | 0 | #DIV/0! |
| ZNFX1 | 1 | 1 | 0 | 1 | 0 | 1 | 2.4 | 81.151 | 707 | 0.0047619 | 6.3189 | 0 | 2.4 | 0 | 0 | #DIV/0! |
| MYH10 | 64 | 49 | 0 | 64 | 0 | 49 | 37.5 | 229 | 1976 | 0 | 323.31 | 0 | 37.5 | 0 | 132780000 | 0 |
| MYH9 | 96 | 78 | 0 | 96 | 0 | 78 | 51.5 | 226.53 | 1960 | 0 | 323.31 | 0 | 51.5 | 0 | 473250000 | 0 |
| MYO6 | 21 | 21 | 0 | 21 | 0 | 21 | 20.7 | 145.01 | 1253 | 0 | 264.06 | 0 | 20.7 | 0 | 21239000 | 0 |
| MYL12A | 6 | 6 | 0 | 6 | 0 | 6 | 44.1 | 20.457 | 177 | 0 | 138.87 | 0 | 44.1 | 0 | 64895000 | 0 |
| NAF1 | 3 | 3 | 0 | 3 | 0 | 3 | 7.1 | 53.716 | 494 | 0 | 111.19 | 0 | 7.1 | 0 | 761200 | 0 |
| MYH14 | 16 | 10 | 0 | 16 | 0 | 10 | 9.5 | 227.87 | 1995 | 0 | 104.02 | 0 | 9.5 | 0 | 24842000 | 0 |
| MYL6 | 7 | 7 | 0 | 7 | 0 | 7 | 52.4 | 16.29 | 145 | 0 | 93.248 | 0 | 52.4 | 0 | 42356000 | 0 |
| MYO1D | 11 | 11 | 0 | 11 | 0 | 11 | 16 | 116.2 | 1006 | 0 | 79.938 | 0 | 16 | 0 | 10848000 | 0 |
| RPL9 | 4 | 4 | 2 | 4 | 2 | 4 | 28.1 | 21.863 | 192 | 0 | 67.489 | 17.2 | 28.1 | 0 | 5515500 | 0 |
| MYO1C | 10 | 10 | 0 | 10 | 0 | 10 | 11.6 | 118.99 | 1039 | 0 | 66.368 | 0 | 11.6 | 0 | 9461900 | 0 |
| KRT2 | 9 | 5 | 3 | 9 | 0 | 5 | 19.9 | 65.432 | 639 | 0 | 55.779 | 5 | 19.9 | 0 | 6784000 | 0 |
| PKM | 6 | 6 | 2 | 4 | 2 | 4 | 18.1 | 57.936 | 531 | 0 | 47.401 | 6.2 | 11.9 | 0 | 5982200 | 0 |
| SNRNP70 | 3 | 3 | 1 | 2 | 1 | 2 | 8.7 | 51.556 | 437 | 0 | 43.826 | 3 | 5.7 | 0 | 524970 | 0 |
| DHX36 | 3 | 3 | 1 | 3 | 1 | 3 | 5.5 | 91.43 | 797 | 0 | 43.339 | 1.8 | 5.5 | 0 | 964320 | 0 |
| MYO1B | 4 | 4 | 0 | 4 | 0 | 4 | 4.6 | 128.48 | 1107 | 0 | 42.602 | 0 | 4.6 | 0 | 2016000 | 0 |
| GNB1 | 5 | 2 | 0 | 5 | 0 | 2 | 16.8 | 37.377 | 340 | 0 | 39.62 | 0 | 16.8 | 0 | 8636800 | 0 |
| TRIM41 | 3 | 3 | 2 | 3 | 2 | 3 | 6.2 | 71.669 | 630 | 0 | 39.153 | 4.3 | 6.2 | 0 | 3158200 | 0 |
| RSL1D1 | 5 | 5 | 2 | 4 | 2 | 4 | 15.3 | 48.208 | 430 | 0 | 38.361 | 8.1 | 11.9 | 0 | 2661100 | 0 |
| YBX3 | 4 | 1 | 4 | 4 | 1 | 1 | 11.3 | 40.089 | 372 | 0 | 35.181 | 11.3 | 11.3 | 0 | 1302300 | 0 |
| LYAR | 3 | 3 | 1 | 3 | 1 | 3 | 13.7 | 43.614 | 379 | 0 | 33.893 | 3.7 | 13.7 | 0 | 1250500 | 0 |

| | | | | | | | | | | | | | | | | |
|---|---|---|---|---|---|---|---|---|---|---|---|---|---|---|---|---|
| SRSF9 | 4 | 3 | 1 | 4 | 0 | 3 | 18.1 | 25.542 | 221 | 0 | 32.027 | 3.2 | 18.1 | 0 | 3614900 | 0 |
| RPL35 | 3 | 3 | 2 | 3 | 2 | 3 | 16.3 | 14.551 | 123 | 0 | 30.884 | 16.3 | 16.3 | 0 | 9918800 | 0 |
| NAT10 | 3 | 3 | 1 | 2 | 1 | 2 | 4.8 | 93.533 | 834 | 0 | 29.749 | 1.2 | 3.6 | 0 | 1302500 | 0 |
| TOP1 | 4 | 4 | 0 | 4 | 0 | 4 | 7.6 | 90.725 | 765 | 0 | 29.2 | 0 | 7.6 | 0 | 3905700 | 0 |
| PRPF19 | 4 | 4 | 2 | 3 | 2 | 3 | 14.7 | 55.18 | 504 | 0 | 27.463 | 7.9 | 9.5 | 0 | 3420400 | 0 |
| EIF2S2 | 4 | 4 | 1 | 4 | 1 | 4 | 11.4 | 38.388 | 333 | 0 | 26.971 | 3 | 11.4 | 0 | 2110000 | 0 |
| FXR2 | 5 | 3 | 3 | 3 | 1 | 2 | 11.1 | 74.222 | 673 | 0 | 25.838 | 6.1 | 7.9 | 0 | 2134200 | 0 |
| EIF2S1 | 2 | 2 | 1 | 2 | 1 | 2 | 7.9 | 36.112 | 315 | 0 | 25.595 | 3.8 | 7.9 | 0 | 1114500 | 0 |
| NOP56 | 4 | 4 | 0 | 4 | 0 | 4 | 7.9 | 66.049 | 594 | 0 | 24.335 | 0 | 7.9 | 0 | 2333800 | 0 |
| SNRPD1 | 2 | 2 | 1 | 2 | 1 | 2 | 44 | 8.3926 | 75 | 0 | 23.957 | 17.3 | 44 | 0 | 3065900 | 0 |
| CFL1 | 3 | 3 | 2 | 2 | 2 | 2 | 32.1 | 17.777 | 159 | 0 | 23.719 | 25.2 | 24.5 | 0 | 1537800 | 0 |
| FMR1 | 4 | 3 | 2 | 4 | 1 | 3 | 12.9 | 47.897 | 419 | 0 | 21.483 | 6.4 | 12.9 | 0 | 1922800 | 0 |
| CD59 | 3 | 3 | 0 | 3 | 0 | 3 | 29.6 | 11.985 | 108 | 0 | 20.872 | 0 | 29.6 | 0 | 2952600 | 0 |
| KRT5 | 5 | 3 | 1 | 5 | 0 | 3 | 9.3 | 62.378 | 590 | 0 | 20.622 | 2 | 9.3 | 0 | 1488200 | 0 |
| CFI | 1 | 1 | 1 | 1 | 1 | 1 | 3.4 | 42.519 | 378 | 0 | 20.39 | 3.4 | 3.4 | 0 | 1334700 | 0 |
| TUBA1C | 19 | 0 | 17 | 15 | 0 | 0 | 43 | 57.73 | 519 | 0 | 19.662 | 40.1 | 35.6 | 0 | 1400800 | 0 |
| OTX1 | 1 | 1 | 1 | 1 | 1 | 1 | 3.4 | 37.327 | 354 | 0 | 19.325 | 3.4 | 3.4 | 0 | 100120 | 0 |
| UBTF | 3 | 3 | 1 | 2 | 1 | 2 | 5 | 87.435 | 745 | 0 | 19.249 | 1.5 | 3.5 | 0 | 1615900 | 0 |
| IGHV1-45 | 1 | 1 | 1 | 1 | 1 | 1 | 9.4 | 13.508 | 117 | 0 | 18.731 | 9.4 | 9.4 | 0 | 1511600 | 0 |
| DRG1 | 3 | 3 | 0 | 3 | 0 | 3 | 11.2 | 40.542 | 367 | 0 | 18.367 | 0 | 11.2 | 0 | 1205700 | 0 |
| PURA | 2 | 2 | 1 | 2 | 1 | 2 | 16.1 | 34.91 | 322 | 0 | 18.116 | 9 | 16.1 | 0 | 646830 | 0 |
| MRPS22 | 3 | 3 | 1 | 2 | 1 | 2 | 16.6 | 36.805 | 319 | 0 | 18.078 | 3.4 | 13.2 | 0 | 452550 | 0 |
| NAP1L1 | 3 | 3 | 1 | 3 | 1 | 3 | 22 | 20.966 | 177 | 0 | 17.61 | 10.2 | 22 | 0 | 2379000 | 0 |
| CDC5L | 3 | 3 | 1 | 2 | 1 | 2 | 4.2 | 92.25 | 802 | 0 | 17.52 | 1.2 | 3 | 0 | 2863000 | 0 |
| NACA | 1 | 1 | 0 | 1 | 0 | 1 | 21.1 | 7.813 | 71 | 0 | 17.284 | 0 | 21.1 | 0 | 473990 | 0 |
| C4B | 1 | 1 | 1 | 1 | 1 | 1 | 0.8 | 187.67 | 1698 | 0 | 16.92 | 0.8 | 0.8 | 0 | 2627000 | 0 |
| TRA2A | 1 | 1 | 1 | 1 | 1 | 1 | 5 | 32.688 | 282 | 0 | 16.563 | 5 | 5 | 0 | 771070 | 0 |
| SF3B1 | 2 | 2 | 1 | 2 | 1 | 2 | 2.4 | 145.83 | 1304 | 0 | 16.022 | 1.2 | 2.4 | 0 | 940570 | 0 |
| GIGYF2 | 2 | 2 | 1 | 2 | 1 | 2 | 3.9 | 96.24 | 836 | 0 | 15.67 | 2 | 3.9 | 0 | 1222200 | 0 |
| DDX6 | 1 | 1 | 0 | 1 | 0 | 1 | 12.8 | 20.426 | 187 | 0 | 15.308 | 0 | 12.8 | 0 | 199550 | 0 |
| CAPRIN1 | 2 | 2 | 1 | 2 | 1 | 2 | 12.9 | 20.236 | 186 | 0 | 15.224 | 5.9 | 12.9 | 0 | 767340 | 0 |
| FLOT1 | 2 | 2 | 1 | 1 | 1 | 1 | 12.9 | 20.491 | 186 | 0 | 13.703 | 7 | 5.9 | 0 | 846110 | 0 |
| RBPMS | 1 | 1 | 0 | 1 | 0 | 1 | 8 | 24.997 | 224 | 0 | 13.64 | 0 | 8 | 0 | 482150 | 0 |
| RPL36 | 2 | 2 | 0 | 2 | 0 | 2 | 21 | 12.254 | 105 | 0 | 13.127 | 0 | 21 | 0 | 808560 | 0 |
| DSG2 | 1 | 1 | 0 | 1 | 0 | 1 | 1.2 | 122.29 | 1118 | 0 | 12.672 | 0 | 1.2 | 0 | 441180 | 0 |
| KRT14 | 3 | 1 | 0 | 3 | 0 | 1 | 6.1 | 51.561 | 472 | 0 | 12.672 | 0 | 6.1 | 0 | 341310 | 0 |
| SNRPA | 2 | 2 | 1 | 2 | 1 | 2 | 20.7 | 10.083 | 87 | 0 | 12.227 | 11.5 | 20.7 | 0 | 1811800 | 0 |
| PRPF40A | 2 | 2 | 1 | 2 | 1 | 2 | 2.7 | 108.8 | 957 | 0 | 12.173 | 1.7 | 2.7 | 0 | 1368500 | 0 |
| STAU1 | 2 | 2 | 1 | 2 | 1 | 2 | 3.7 | 54.945 | 493 | 0 | 12.101 | 2 | 3.7 | 0 | 1710100 | 0 |
| FN1 | 2 | 2 | 1 | 2 | 1 | 2 | 0.9 | 262.62 | 2386 | 0 | 12.077 | 0.3 | 0.9 | 0 | 2527100 | 0 |
| THRAP3 | 2 | 2 | 0 | 2 | 0 | 2 | 4.1 | 108.66 | 955 | 0 | 12.024 | 0 | 4.1 | 0 | 615390 | 0 |
| SEC61B | 1 | 1 | 1 | 1 | 1 | 1 | 15.6 | 9.9743 | 96 | 0 | 11.993 | 15.6 | 15.6 | 0 | 1577400 | 0 |
| RPL29 | 1 | 1 | 1 | 1 | 1 | 1 | 9.4 | 17.752 | 159 | 0 | 11.907 | 9.4 | 9.4 | 0 | 8460700 | 0 |
| TIA1 | 3 | 1 | 3 | 3 | 1 | 1 | 9.4 | 42.835 | 385 | 0 | 11.868 | 9.4 | 9.4 | 0 | 409840 | 0 |
| TMF1 | 2 | 2 | 1 | 2 | 1 | 2 | 1.7 | 122.84 | 1093 | 0 | 11.763 | 0.9 | 1.7 | 0 | 90287000 | 0 |
| MAGOHB | 2 | 2 | 1 | 2 | 1 | 2 | 33.3 | 10.715 | 90 | 0 | 11.646 | 22.2 | 33.3 | 0 | 1391200 | 0 |
| AP2B1 | 2 | 2 | 1 | 2 | 1 | 2 | 7.4 | 70.916 | 639 | 0 | 11.565 | 3.3 | 7.4 | 0 | 140180 | 0 |
| | 1 | 1 | 1 | 1 | 1 | 1 | 9.4 | 12.822 | 117 | 0 | 11.562 | 9.4 | 9.4 | 0 | 838170 | 0 |
| TUBB2B | 20 | 1 | 20 | 18 | 1 | 1 | 60.2 | 49.953 | 445 | 0 | 11.543 | 60.2 | 55.1 | 0 | 2714800 | 0 |
| ADH7 | 2 | 2 | 1 | 1 | 1 | 1 | 4.7 | 41.481 | 386 | 0 | 11.402 | 2.6 | 4.4 | 0 | 350330 | 0 |
| MYL1 | 2 | 2 | 0 | 2 | 0 | 2 | 8.2 | 21.145 | 194 | 0 | 11.263 | 0 | 8.2 | 0 | 1805500 | 0 |
| GTPBP1 | 2 | 2 | 0 | 2 | 0 | 2 | 4.2 | 72.453 | 669 | 0 | 11.176 | 0 | 4.2 | 0 | 867780 | 0 |
| TRA2B | 2 | 2 | 1 | 1 | 1 | 1 | 18.7 | 12.548 | 107 | 0 | 11.071 | 9.3 | 9.3 | 0 | 508180 | 0 |
| GNAI3 | 2 | 2 | 0 | 2 | 0 | 2 | 5.4 | 40.532 | 354 | 0 | 10.869 | 0 | 5.4 | 0 | 1880000 | 0 |
| USP47 | 2 | 2 | 1 | 1 | 1 | 1 | 1.9 | 157.31 | 1375 | 0 | 10.843 | 0.6 | 1.3 | 0 | 510140 | 0 |
| C2orf47 | 2 | 2 | 0 | 2 | 0 | 2 | 12.4 | 25.006 | 225 | 0 | 10.828 | 0 | 12.4 | 0 | 329390 | 0 |
| STRBP | 4 | 2 | 1 | 4 | 0 | 2 | 5.8 | 73.652 | 672 | 0 | 10.803 | 2.1 | 5.8 | 0 | 941160 | 0 |
| NSUN2 | 1 | 1 | 1 | 1 | 1 | 1 | 1.4 | 86.47 | 767 | 0 | 10.395 | 1.4 | 1.4 | 0 | 645000 | 0 |
| HSPA6 | 5 | 1 | 5 | 5 | 1 | 1 | 10.6 | 71.027 | 643 | 0 | 10.016 | 10.6 | 10.6 | 0 | 7283700 | 0 |
| NOLC1 | 1 | 1 | 1 | 1 | 1 | 1 | 21.7 | 5.0538 | 46 | 0 | 9.9855 | 21.7 | 21.7 | 0 | 1633200 | 0 |
| UBA52 | 3 | 1 | 2 | 1 | 0 | 1 | 27.3 | 14.728 | 128 | 0 | 9.718 | 19.5 | 7.8 | 0 | 114310 | 0 |
| MRPS9 | 1 | 1 | 1 | 1 | 1 | 1 | 3 | 45.834 | 396 | 0 | 9.2056 | 3 | 3 | 0 | 426210 | 0 |
| RBPMS2 | 1 | 1 | 0 | 1 | 0 | 1 | 14.4 | 22.496 | 209 | 0 | 9.1805 | 0 | 14.4 | 0 | 563620 | 0 |
| RC3H2 | 1 | 1 | 0 | 1 | 0 | 1 | 2.4 | 56.823 | 506 | 0 | 9.1752 | 0 | 2.4 | 0 | 250860 | 0 |
| MRPS17 | 1 | 1 | 1 | 1 | 1 | 1 | 22.5 | 14.374 | 129 | 0 | 9.1652 | 22.5 | 22.5 | 0 | 2820300 | 0 |
| BUB3 | 1 | 1 | 0 | 1 | 0 | 1 | 9 | 16.28 | 145 | 0 | 9.1228 | 0 | 9 | 0 | 526790 | 0 |
| RAP1B | 1 | 1 | 0 | 1 | 0 | 1 | 11.7 | 11.916 | 103 | 0 | 8.7407 | 0 | 11.7 | 0 | 221960 | 0 |
| TRIM25 | 1 | 1 | 1 | 1 | 1 | 1 | 2.1 | 70.973 | 630 | 0 | 8.7137 | 2.1 | 2.1 | 0 | 9383800 | 0 |
| RPL38 | 1 | 1 | 1 | 1 | 1 | 1 | 61.9 | 2.559 | 21 | 0 | 8.6767 | 61.9 | 61.9 | 0 | 3387900 | 0 |
| IGHV1OR15 | 1 | 1 | 1 | 1 | 1 | 1 | 9.4 | 13.012 | 117 | 0 | 8.56 | 9.4 | 9.4 | 0 | 527970 | 0 |
| GAPDH | 1 | 1 | 0 | 1 | 0 | 1 | 5.4 | 27.87 | 260 | 0 | 8.5582 | 0 | 5.4 | 0 | 462180 | 0 |
| SETD2 | 1 | 1 | 0 | 1 | 0 | 1 | 0.9 | 145.46 | 1290 | 0 | 8.527 | 0 | 0.9 | 0 | 327580 | 0 |
| RPLP2 | 1 | 1 | 1 | 1 | 1 | 1 | 13 | 8.9857 | 92 | 0 | 8.4537 | 13 | 13 | 0 | 1986400 | 0 |
| SMPDL3B | 1 | 1 | 0 | 1 | 0 | 1 | 2.5 | 45.314 | 407 | 0 | 8.041 | 0 | 2.5 | 0 | 1209400 | 0 |
| AIFM1 | 1 | 1 | 1 | 1 | 1 | 1 | 4 | 29.938 | 274 | 0 | 8.001 | 4 | 4 | 0 | 595960 | 0 |
| MAZ | 1 | 1 | 1 | 1 | 1 | 1 | 8.4 | 22.187 | 202 | 0 | 7.775 | 8.4 | 8.4 | 0 | 1207300 | 0 |
| SNRPE | 1 | 1 | 1 | 1 | 1 | 1 | 12 | 10.803 | 92 | 0 | 7.7649 | 12 | 12 | 0 | 540090 | 0 |
| NUDT16L1 | 1 | 1 | 0 | 1 | 0 | 1 | 5.8 | 18.944 | 171 | 0 | 7.6917 | 0 | 5.8 | 0 | 404090 | 0 |
| AHSA1 | 1 | 1 | 1 | 1 | 1 | 1 | 24.4 | 10.031 | 90 | 0 | 7.6321 | 24.4 | 24.4 | 0 | 479700 | 0 |
| NXPE4 | 1 | 1 | 0 | 1 | 0 | 1 | 2 | 62.262 | 544 | 0 | 7.403 | 0 | 2 | 0 | 3608600 | 0 |
| TRAJ56 | 1 | 1 | 1 | 1 | 1 | 1 | 38.1 | 2.1104 | 21 | 0 | 7.3424 | 38.1 | 38.1 | 0 | 253080000 | 0 |
| MMTAG2 | 1 | 1 | 0 | 1 | 0 | 1 | 5.3 | 29.411 | 263 | 0 | 7.3241 | 0 | 5.3 | 0 | 292900 | 0 |
| RPL36AL | 4 | 1 | 2 | 4 | 0 | 1 | 26.4 | 12.469 | 106 | 0 | 7.2792 | 8.5 | 26.4 | 0 | 1476700 | 0 |
| FAM120A | 1 | 1 | 1 | 1 | 1 | 1 | 2.6 | 54.06 | 495 | 0 | 7.1787 | 2.6 | 2.6 | 0 | 473530 | 0 |
| F2 | 1 | 1 | 1 | 1 | 1 | 1 | 2.8 | 35.931 | 324 | 0 | 7.0486 | 2.8 | 2.8 | 0 | 854650 | 0 |
| JUP | 1 | 1 | 0 | 1 | 0 | 1 | 2.7 | 81.744 | 745 | 0 | 6.9956 | 0 | 2.7 | 0 | 854640 | 0 |
| TUBAL3 | 4 | 1 | 4 | 3 | 1 | 1 | 9.4 | 49.908 | 446 | 0 | 6.995 | 9.4 | 7.8 | 0 | 2390300 | 0 |
| KRT72 | 1 | 1 | 0 | 1 | 0 | 1 | 6.2 | 21.653 | 192 | 0.0025575 | 6.9213 | 0 | 6.2 | 0 | 719170 | 0 |
| SLC39A7 | 1 | 1 | 1 | 1 | 1 | 1 | 3 | 50.117 | 469 | 0.002551 | 6.9185 | 3 | 3 | 0 | 5283400 | 0 |
| G3BP2 | 1 | 1 | 0 | 1 | 0 | 1 | 3.5 | 54.12 | 482 | 0.0025445 | 6.918 | 0 | 3.5 | 0 | 322160 | 0 |
| IGF2BP2 | 2 | 1 | 1 | 2 | 0 | 1 | 4.1 | 66.785 | 605 | 0.0025381 | 6.8919 | 2.1 | 4.1 | 0 | 1005200 | 0 |
| ZNF346 | 1 | 1 | 0 | 1 | 0 | 1 | 11 | 12.817 | 118 | 0.0025316 | 6.8347 | 0 | 11 | 0 | 224900 | 0 |
| ALYREF | 1 | 1 | 1 | 1 | 1 | 1 | 4.2 | 27.557 | 264 | 0.0025189 | 6.8083 | 4.2 | 4.2 | 0 | 1189000 | 0 |
| UBAP2L | 1 | 1 | 1 | 1 | 1 | 1 | 5.2 | 40.109 | 383 | 0.0025126 | 6.8029 | 5.2 | 5.2 | 0 | 278270 | 0 |
| DDX18 | 1 | 1 | 0 | 1 | 0 | 1 | 1.5 | 75.406 | 670 | 0.0025063 | 6.7924 | 0 | 1.5 | 0 | 874780 | 0 |
| GNG12 | 1 | 1 | 0 | 1 | 0 | 1 | 15.3 | 8.0061 | 72 | 0.0025 | 6.7532 | 0 | 15.3 | 0 | 919090 | 0 |
| LARP4 | 1 | 1 | 1 | 1 | 1 | 1 | 10.4 | 14.143 | 125 | 0.0024938 | 6.7494 | 10.4 | 10.4 | 0 | 323690 | 0 |
| RPLP1 | 1 | 1 | 1 | 1 | 1 | 1 | 14 | 11.514 | 114 | 0.0024876 | 6.6698 | 14 | 14 | 0 | 2918300 | 0 |
| FBL | 1 | 1 | 1 | 1 | 1 | 1 | 5.7 | 16.164 | 157 | 0.002457 | 6.5759 | 5.7 | 5.7 | 0 | 3584100 | 0 |
| COPA | 1 | 1 | 0 | 1 | 0 | 1 | 1.8 | 138.34 | 1224 | 0.002451 | 6.5675 | 0 | 1.8 | 0 | 69394 | 0 |
| RRP1B | 1 | 1 | 0 | 1 | 0 | 1 | 2.8 | 84.427 | 758 | 0.002445 | 6.5559 | 0 | 2.8 | 0 | 1381400 | 0 |
| RNMTL1 | 1 | 1 | 0 | 1 | 0 | 1 | 10 | 12.597 | 110 | 0.0024331 | 6.5234 | 0 | 10 | 0 | 209040 | 0 |
| PRPF8 | 1 | 1 | 1 | 1 | 1 | 1 | 2.1 | 119.7 | 1012 | 0.0024272 | 6.5134 | 2.1 | 2.1 | 0 | 304520 | 0 |
| TGM2 | 1 | 1 | 1 | 1 | 1 | 1 | 1.9 | 77.328 | 687 | 0.0024155 | 6.4961 | 1.9 | 1.9 | 0 | 197530 | 0 |
| IGKV4-1 | 1 | 1 | 1 | 1 | 1 | 1 | 7.4 | 13.38 | 121 | 0.0024096 | 6.4647 | 7.4 | 7.4 | 0 | 1154300 | 0 |
| RRP1 | 1 | 1 | 1 | 1 | 1 | 1 | 20 | 5.203 | 45 | 0.0023981 | 6.4428 | 20 | 20 | 0 | 524680 | 0 |
| YTHDF1 | 2 | 1 | 1 | 2 | 0 | 1 | 8.1 | 60.873 | 559 | 0.0023923 | 6.4272 | 1.6 | 8.1 | 0 | 353570 | 0 |
| DARS | 1 | 1 | 0 | 1 | 0 | 1 | 11.4 | 19.868 | 175 | 0.0047393 | 6.2871 | 0 | 11.4 | 0 | 1127000 | 0 |
| GNB2 | 4 | 1 | 0 | 4 | 0 | 1 | 14.5 | 32.408 | 296 | 0.0047281 | 6.2437 | 0 | 14.5 | 0 | 739930 | 0 |

| | | | | | | | | | | | | | | | | |
|---|---|---|---|---|---|---|---|---|---|---|---|---|---|---|---|---|
| RBMXL1 | 7 | 1 | 6 | 5 | 1 | 1 | 16.7 | 42.141 | 390 | 0.0047059 | 6.2076 | 16.7 | 11.8 | 0 | 1192500 | 0 |
| RAN | 1 | 1 | 0 | 1 | 0 | 1 | 10.2 | 14.731 | 128 | 0.0046948 | 6.2011 | 0 | 10.2 | 0 | 678110 | 0 |
| IGLL5 | 1 | 1 | 1 | 1 | 1 | 1 | 3.7 | 23.15 | 215 | 0.0046729 | 6.1897 | 3.7 | 3.7 | 0 | 1794500 | 0 |
| SH2D4A | 1 | 1 | 0 | 1 | 0 | 1 | 6.3 | 22.649 | 189 | 0.0046512 | 6.1668 | 0 | 6.3 | 0 | 784930 | 0 |
| ARFGAP1 | 1 | 1 | 1 | 1 | 1 | 1 | 5.7 | 26.555 | 244 | 0.0046296 | 6.1491 | 5.7 | 5.7 | 0 | 102890 | 0 |
| ERH | 1 | 1 | 0 | 1 | 0 | 1 | 15.5 | 8.2033 | 71 | 0.0046189 | 6.1339 | 0 | 15.5 | 0 | 486480 | 0 |
| PFN2 | 1 | 1 | 0 | 1 | 0 | 1 | 15.4 | 9.7982 | 91 | 0.0045977 | 6.0861 | 0 | 15.4 | 0 | 692560 | 0 |
| GSPT2 | 1 | 1 | 1 | 1 | 1 | 1 | 2.7 | 68.882 | 628 | 0.0068182 | 6.0292 | 2.7 | 2.7 | 0 | 191610 | 0 |
| KCTD19 | 1 | 1 | 0 | 1 | 0 | 1 | 2.7 | 104.94 | 926 | 1 | -2 | 0 | 2.7 | 0 | 145630 | 0 |
| DNAJC13 | 1 | 1 | 1 | 1 | 1 | 1 | 0.8 | 254.41 | 2243 | 1 | -2 | 0.8 | 0.8 | 0 | 789320 | 0 |
| TUBB3 | 13 | 0 | 13 | 13 | 0 | 0 | 15.9 | 88.381 | 797 | 1 | -2 | 15.9 | 15.9 | 0 | 206880 | 0 |

**Supplementary Table S3：**

**The correlation of TRIM31 expression and TP53 status of breast cancer patients (OS)**

| Parameters | Cases | TRIM31 expression | | *P* values |
|---|---|---|---|---|
| | | Low | High | |
| | 327 | 163 | 164 | |
| TP53 status | | | | 0.964 |
| Wide type | 197 | 98 | 99 | |
| Mutant | 130 | 65 | 65 | |

**The correlation of TRIM31 expression and TP53 status of breast cancer patients (RFS)**

| Parameters | Cases | TRIM31 expression | | *P* values |
|---|---|---|---|---|
| | | Low | High | |
| | 461 | 234 | 227 | |
| TP53 status | | | | 0.626 |
| Wide type | 273 | 136 | 137 | |
| Mutant | 188 | 98 | 90 | |
