# Supplementary material for: Loss of TRIM31 promotes breast cancer progression through regulating K48- and K63-linked ubiquitination of p53
Source: Cell Death Dis. 2021 Oct 14;12(10):945. doi: 10.1038/s41419-021-04208-3 (PMC8516922; doi:10.1038/s41419-021-04208-3)
Supplement: Supplementary file 2 — Supplementary Figure Legends [file 41419_2021_4208_MOESM2_ESM.doc]

**Supplementary Fig. S1:** The elution proteins of GST, GST-P53 and His-TRIM31 were visualized by coomassie blue staining.

**Supplementary Fig. S2:** TRIM31 overexpression increase the expression of p53

(a, b) The MCF7 (a) and ZR-75-30 (b) cells stably overexpress TRIM31 or control plasmid, the expression of TRIM31 and p53 was detected by western blot. (c, d) the mRNA level of TRIM31 and p53 was detected by real-time PCR in MCF7 (c) and ZR-75-30 (d) cells. Data are shown as the mean ± SD of at least three independent experiments, and the significant level was identified by *P < 0.05, **P < 0.01 and ***P < 0.001

**Supplementary Fig. S3:** TRIM31 neither regulates MDM2 expression nor interacts with MDM2

(a) The MCF7 and ZR-75-30 cells stably interfere with TRIM31 or control, the expression of TRIM31 and MDM2 was detected by western blot. (b) The MCF7 and ZR-75-30 cells stably overexpress TRIM31 or control, the expression of TRIM31 and MDM2 was detected by western blot. (c) The HEK293 cells were transfected with HA-TRIM31 plasmids, and the cells were treated with MG132 (20 μM) for 4h before harvest. The cell lysates were immunoprecipitated with HA-tag antibody and immunobloted with Flag-tag and anti-MDM2 antibody.

**Supplementary Fig. S4:** The elution protein of His-TRIM31, GST-P53 and His-MDM2 were visualized by coomassie blue staining.

**Supplementary Fig. S5: TRIM31 competitive inhibiting the interaction of MDM2 and p53**

The ZR-75-30 cells stably overexpress TRIM31 were transfected with pBIFC-VN173-Flag-p53 and pBIFC-VC155-HA-MDM2 plasmids, and the cells were further cultured for 48 h. The cells were imaged by Olympus fluorescence microscope, and the relative fluorescence intensity of cells was measured using a fluorescence microplate reader. Scale bar: 10 μm. Data are shown as the mean ± SD of at least three independent experiments, and the significant level was identified by *P < 0.05, **P < 0.01 and ***P < 0.001.

**Supplementary Fig. S6: Overexpression of MDM2 decreased the interaction of TRIM31 and p53**

(a) The MCF7 cells were transfected with different amounts of Flag-MDM2 plasmids, the cell lysates were immunoprecipitated with anti-P53 antibody and then western blot assay with Flag-tag, TRIM31 and p53 antibody. (b) The MCF7 cells were transfected with different amounts of Flag-MDM2 and HA-TRIM31 plasmids, the cell lysates were immunoprecipitated with anti-HA antibody and then western blot assay with anti-Flag, anti-HA and anti-p53 antibody.

**Supplementary Fig. S7: Knockout of MDM2 rescued the tumor promotion function of TRIM31-deficient in MCF7 cells**

(a) The MCF7 cells stably interfere with TRIM31 were transfected with sg-MDM2 plasmid, and the cells were further cultured for 48 h, the expression of TRIM31, p53, p21 and BAX were detected by western blot. (b) The cell survival of MCF7 at 72h was detected by CCK8 assay. (c) The colony formation was used to detect the cell proliferation of MCF7 cells. Data are shown as the mean ± SD of at least three independent experiments, and the significant level was identified by *P < 0.05, **P < 0.01 and ***P < 0.001.

**Supplementary Fig. S8:** Restoration of p53 expression rescue the tumor promotion function of TRIM31 knockdown in MCF7 cells.

(a) The MCF7 cells stably interfere with TRIM31 were treated with the p53 activator Nutlin3 (10 μM) for 48h. The expression of TRIM31, p53, p21 and BAX were detected by western blot. (b) The cell survival of MCF7 at 72h was detected by CCK8 assay. (c) The colony formation was used to detect the cell proliferation of MCF7 cells. (d) The migration capability of MCF7 were detected by transwell migration assay. Data are shown as the mean ± SD of at least three independent experiments, and the significant level was identified by *P < 0.05, **P < 0.01 and ***P < 0.001

**Supplementary Fig. S9:** Inhibition of p53 expression rescue the tumor suppressor function of TRIM31 overexpression in ZR-75-30 cells.

(a) The ZR-75-30 cells stably overexpress TRIM31 were treated with the p53 inhibitor PFTα (15 μM) for 48h. The expression of TRIM31, p53, p21 and BAX were detected by western blot. (b) The cell survival of ZR-75-30 at 72h was detected by CCK8 assay. (c) The colony formation was used to detect the cell proliferation of ZR-75-30 cells. (d) The transwell migration assay was performed to detect the migration capability of ZR-75-30 cells. Data are shown as the mean ± SD of at least three independent experiments, and the significant level was identified by *P < 0.05, **P < 0.01 and ***P < 0.001.

**Supplementary Fig. S10: Overexpression of MDM2 rescued the tumor suppression function of TRIM31-overexpression in vivo**

(a) About 3 × 10^6^ of ZR-75-30 cells stably express HA-TRIM31, Flag-MDM2 and control (Mock+Vector, TRIM31+Vector, Mock+MDM2, TRIM31+MDM2) were injected into 6-week old female nude mice. The images showed the tumors from these four groups. The growth curve (b), tumor volume (c) and tumor weight (d) of these four groups were statistically analyzed at 40 days after injection.

**Supplementary Table S1:** The correlation of TRIM31 expression and TP53 status of breast cancer patients.

**Supplementary Table S2:** The up and down-regulated proteins in response to TRIM31 knockdown in MCF7 cells.

**Supplementary Table S3:** The proteins interacted with TRIM31 were identified by CO-IP and mass spectrometry (MS)
